# Long-term ecological stability in the Pleistocene central Levantine corridor revealed by enamel isotopes from Lebanon

**DOI:** 10.1038/s43247-026-03925-x

**Published:** 2026-09-14

**Authors:** Gabriele Russo, Faysal Bibi, Chrysa Tsimopoulou, Mathieu Duval, Yamandù H. Hilbert, Maya Haïdar-Boustani, Dorothée G. Drucker, Sireen El Zaatari

**Affiliations:** 1 Paleoanthropology, Senckenberg Centre for Human Evolution and Palaeoenvironment, University of Tübingen, Tübingen, Germany; 2 Museum für Naturkunde, Leibniz Institute for Evolution and Biodiversity Science, Berlin, Germany; 3 Laboratory of Biological Anthropology, Department of Humanities, Democritus University of Thrace, University Campus T.C., Komotini, Greece; 4 Institute of Appied Bioarchaeological Research, Democritus University of Thrace, Komotini, Greece; 5 Geochronology & Geology, Centro Nacional de Investigación sobre la Evolución Humana (CENIEH), Burgos, Spain; 6 Australian Research Centre for Human Evolution (ARCHE), Griffith University, Brisbane, QLD Australia; 7 Palaeoscience Labs, Dept. Archaeology and History, La Trobe University, Melbourne Campus, Bundoora, VIC Australia; 8 HUMAN ORIGINS - Cluster of Excellence for Integrative Human Origins Studies (EXC 3101), University of Tübingen, Tübingen, Germany; 9 Musée de Préhistoire libanaise, Université Saint-Joseph de Beyrouth, Beirut, Lebanon; 10 Senckenberg Centre for Human Evolution and Palaeoenvironment, University of Tübingen, Tübingen, Germany; 11 Biogeology, Department of Geosciences, University of Tübingen, Tübingen, Germany

**Keywords:** Palaeoecology, Archaeology, Palaeoclimate

## Abstract

Throughout the Pleistocene, the Levant served as a key land bridge between continents and witnessed repeated hominin dispersals, including the major expansion of modern humans out of Africa. Yet despite this strategic role, the local ecological conditions encountered by hominin populations along the central Levant remain poorly understood. Here, we analyze sequential and bulk δ^13^C and δ^18^O enamel values from eight herbivore species across three Lebanese archaeological sites, Naame, Nahr Ibrahim, and Ras el-Kelb, spanning ~400,000 to ~100,000 years ago, to reconstruct vegetation structure, hydrology, and seasonal temperature regimes experienced by the hominins who lived in this region. Our results show that the central Levant maintained persistently regular Mediterranean seasonality, with consistent summer-winter offsets and minimal chronological change in amplitude across multiple glacial-interglacial cycles, despite pronounced regional and global climatic oscillations. This reliable hydroclimate supported C3-dominated woodlands and mesic, heterogeneous habitats that maintained reliable resources for herbivore communities over nearly 300,000 years. Together, these findings identify the Lebanese coast as a persistent ecological refugium offering resource predictability that likely facilitated repeated hominin occupations and dispersal events through the eastern Mediterranean.

## Abstract (web summary)

The central Levant maintained stable Mediterranean seasonality and resource-rich C3 woodlands across multiple glacial-interglacial cycles, acting as a persistent ecological refugium for hominins, based on enamel isotopic records from Lebanon.

## Introduction

The successful expansion of multiple hominin species from Africa into Eurasia required not only physiologically and behaviorally adapted populations but also ecologically viable corridors, i.e., landscapes where water, vegetation, and prey resources would support and sustain their movements into and occupations of new territories. The Levantine corridor, representing a long-standing terrestrial land bridge between Africa and Eurasia and positioned at the interface of Mediterranean, Saharan, and Eurasian climatic systems, would have formed one of these viable corridors^1,2^, giving it a central role in human evolutionary studies. The Levant’s exceptionally rich Pleistocene archaeological record documents various hominins occupations and cultural turnovers^3,4^. These are believed to have been influenced by climatic and environmental fluctuations, which seem to have coincided with global glacial/interglacial cycles that shaped ecological conditions, including faunal communities^5–7^. Understanding the environmental and ecological dynamics that allowed hominin populations to thrive in the Levant is therefore essential for interpreting their dispersal and occupation patterns, adaptive strategies, and cultural change.

A wealth of paleoenvironmental proxies, including lake and marine sediment cores, speleothems, and pollen records are available for the Levant, e.g., ref. ^2^. These provide climatic and ecological reconstructions for the region spanning multiple glacial-interglacial cycles, revealing pronounced climatic oscillations, including shifts in moisture availability that seem to be variably expressed regionally (e.g., refs. ^8–15^). These proxies, however, are often not directly associated with archaeological contexts or hominin activity. Moreover, where such direct associations exist, they are primarily in the southern part of the Levant^16,17^, leaving northern regions comparatively underrepresented, with only a limited number of studies from Lebanon beginning to address this gap^18,19^.

Considering that collectively, the environmental proxies also reveal a gradient of increasing aridity from north to south across the Levant, broadly resembling modern patterns, generalizing interpretations of potential influences of southern Levantine patterns on hominin occupations across to the more northern Levantine regions becomes problematic. Although sparse, paleoenvironmental data from the latter indicate that the central and northern coastal zone was more strongly influenced by Mediterranean climatic systems rather than by the African monsoon, which affects parts of the southern Levant^20,21^. This configuration likely resulted in the Lebanese coastal-mountain zone in the central Levant being a relatively buffered ecological setting, where the combination of orographic precipitation and sharp elevational gradients would have potentially continued to sustain local mesic habitats (i.e., potential micro‑refugia) even as the broader eastern Mediterranean hydroclimate fluctuated across glacial-interglacial cycles^21–23^. These differences in water availability and its associated impact on regional paleohabitat underscores that the Levant was never a climatically homogeneous region.

Lebanon occupies a central position along the Levantine corridor; however, no high-resolution palaeoclimate reconstructions linked to human occupation exist from the country. Earlier paleoecological studies based on microfauna, avifauna, and molluscan assemblages, largely confined to the archeological site of Ksar’Akil and focused on the Upper Paleolithic (~50–20 ka), provided important but chronologically restricted insights into local environmental conditions^18,19,24–26^. Additionally, pollen data from key archeological sites, such as Adlun, Ras el-Kelb Cave, Nahr Ibrahim, Naame, and Batroun, have offered valuable but fragmentary glimpses into vegetation dynamics during the Lower and Middle Paleolithic^27,28^ (~500–50 ka). However, these studies are limited by reduced sample sizes and poor chronostratigraphic control, leaving a significant gap in our understanding of the environmental backdrop to hominin occupation in Lebanon.

**Fig. 1:**
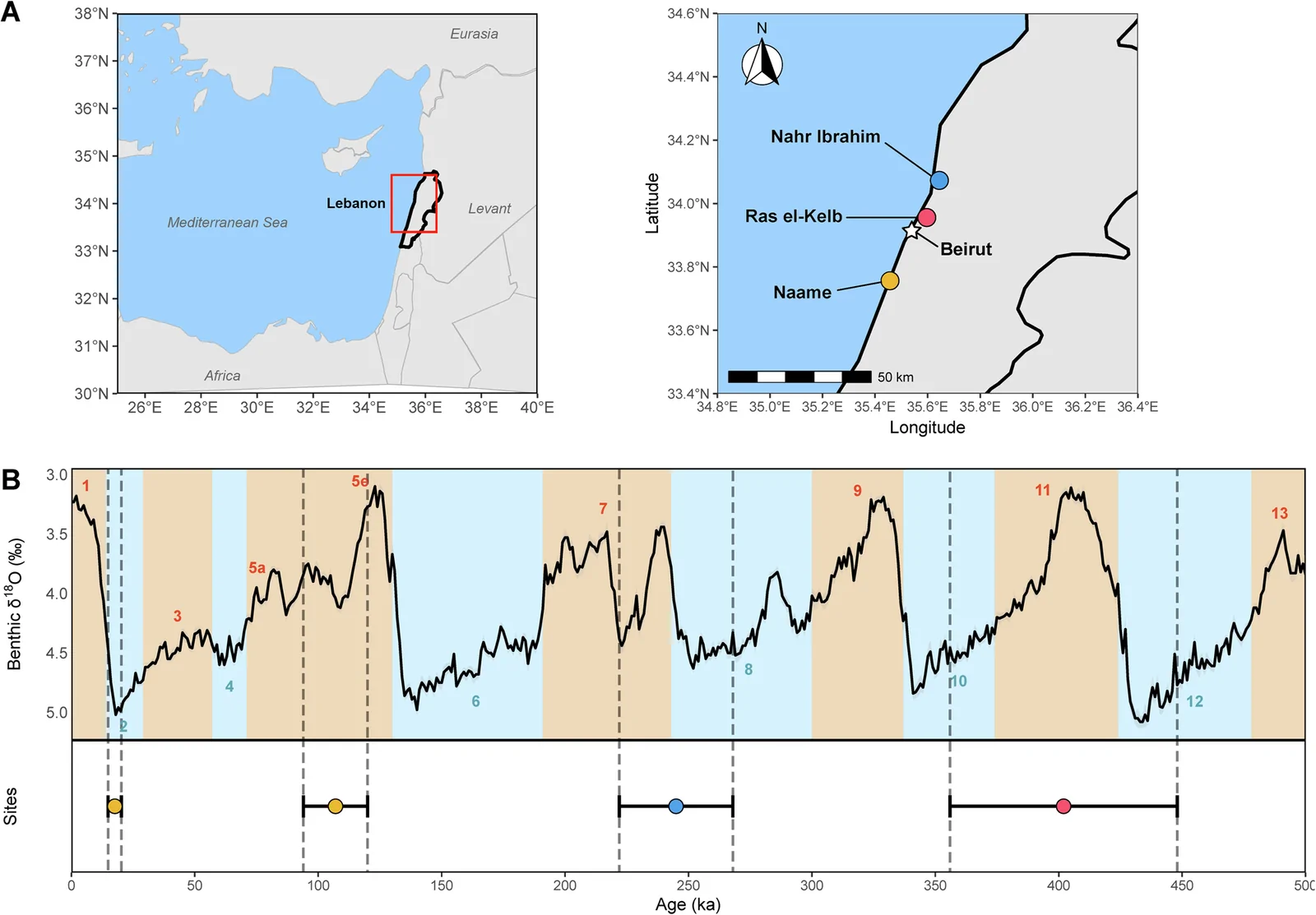
Geographic location and chronological context of the studied sites. **A** Maps showing the position of Naame, Nahr Ibrahim, and Ras el-Kelb along the Lebanese coast of the Eastern Mediterranean. **B** Existing age constraints (1*σ* errors) on the legacy collections^29^, relative to the global benthic δ^18^O stack, illustrating major marine isotope stages (MIS) and associated climatic oscillations. The benthic δ^18^O data are from ref. ^80^.

Recent work on three legacy faunal collections from the Lebanese sites of Naame, Nahr Ibrahim, and Ras el-Kelb has begun to fill this gap. New ESR/U-series dating results place these assemblages to between ~400 and 100 ka, and the associated environmental reconstructions indicate the persistence of Mediterranean woodland ecosystems across multiple interglacials^29^ (Fig. 1). Building on this framework, the present study applies bulk and sequential analysis of enamel carbon- and oxygen-isotope composition (δ^13^C and δ^18^O) to large herbivore teeth from these directly dated assemblages. Enamel carbonate δ^13^C values primarily inform on vegetation structure and thus on dietary niche partitioning in animal species, whereas enamel δ^18^O values reflect drinking water composition and thus offer insights into hydrological conditions and seasonality in the region at the time^30–32^. Bulk samples provide mean annual environmental signals, while sequential intra-tooth sampling captures intra-annual variability and seasonal amplitude. In addition, δ^18^O values were used to estimate paleoclimate variability following established calibration frameworks, e.g., refs. ^33,34^. This approach thus provides new high-resolution insights into vegetation structure, hydrological regimes, seasonal temperature variation, and niche partitioning, directly linked to human populations who inhabited this region during the Paleolithic. Importantly, this study also illustrates how legacy collections can be used to fill gaps in our knowledge of hominin adaptation and dispersal.

## Results

### Preservation

We analysed 30 bulk enamel samples, 295 intra-tooth bands, and 28 dentine samples from aurochs (*Bos primigenius*), wild goat (*Capra aegagrus*), Persian fallow deer (*Dama mesopotamica*), red deer (*Cervus elaphus*), hippopotamus (*Hippopotamus amphibius*), narrow-nosed rhinoceros (*Stephanorinus hemiotoechus*), equids (indet. *Equidae*), and porcupine (*Hystrix* sp.) teeth—some of which were directly dated—recovered from the Lebanese sites of Naame, Nahr Ibrahim, and Ras el-Kelb (Supplementary Tables S1 and S2). Carbonate (CO_3_) contents were found to fall within the expected range for well-preserved enamel across all sites, and no overlap was observed between enamel and dentine carbonate values (Supplementary Table S3) in specimens where both were sampled. Only three bulk samples from Naame showed anomalously high CO_3_ content and inconsistent δ^18^O values and were considered contaminated or altered and thus excluded (Supplementary Result S3.1). Within sequential profiles, identified outliers in the sinusoidal trends (total *n* = 16) based on CO_3_ values or substantial deviation from polynomial δ^18^O curves were excluded from the analysis (Supplementary Result S3.1 and Supplementary Fig. S2).

### Sequential isotopic profiles

Sequential δ^13^C_VPDB_ and δ^18^O_VPDB_ profiles both exhibit sinusoidal cyclicity across taxa and sites (Supplementary Fig. S3.2), reflecting intra-annual environmental and dietary variation. Aurochs in particular consistently display the largest seasonal amplitudes reflecting one to three semiannual cycles of crown growth. At Naame (levels PIII_2_ and PIII_0_), aurochs show the greatest isotopic variability (mean δ^13^C = 2.8‰ ± 1.9 SD and mean δ^18^O = 2.3‰ ± 0.8 SD), while other taxa show slightly smaller intra-tooth ranges (overall, δ^13^C = 0.5–0.9‰; δ^18^O = 1.6–2.3‰) (Supplementary Result S3.2, Supplementary Fig. S2, and Supplementary Table S4). At Naame (mostly constrained to the MIS 5, i.e., 130–71 ka), strong positive δ^13^C-δ^18^O coupling (*r* = 0.75–0.99) with near-synchronous lags (0–5 samples) reflects coherent climatic and dietary responses to marked seasonality. Additionally, the single aurochs specimen from Naame - *Niveau Supérieur,* directly dated to the MIS 2 (~18 ka), similarly preserves pronounced cycles, suggesting persistent seasonality across glacial conditions. At Nahr Ibrahim (245 ± 23 ka, 1*σ*), individual isotopic variability is moderate in comparison with the other two collections. Aurochs show mostly positive correlations between δ^13^C and δ^18^O (*r* > 0.9) but also examples of inverse coupling (*r* ≈ − 0.8), implying seasonal shifts between open and mesic microhabitats. At Ras el-Kelb (402 ± 46 ka, 1*σ*), amplitudes are lower relative to the other sites, and δ^13^C-δ^18^O coupling is weaker (*r* = 0.58–0.91), consistent with locally damped seasonality. Across species, wild goat and fallow deer frequently show negative δ^13^C-δ^18^O correlations (−0.8 to −0.6), reflecting foraging in shaded or moisture-buffered habitats during warm periods. Rhinoceros profiles remain relatively flat with weak coupling.

### Mean δ^13^C isotopic data

**Fig. 2:**
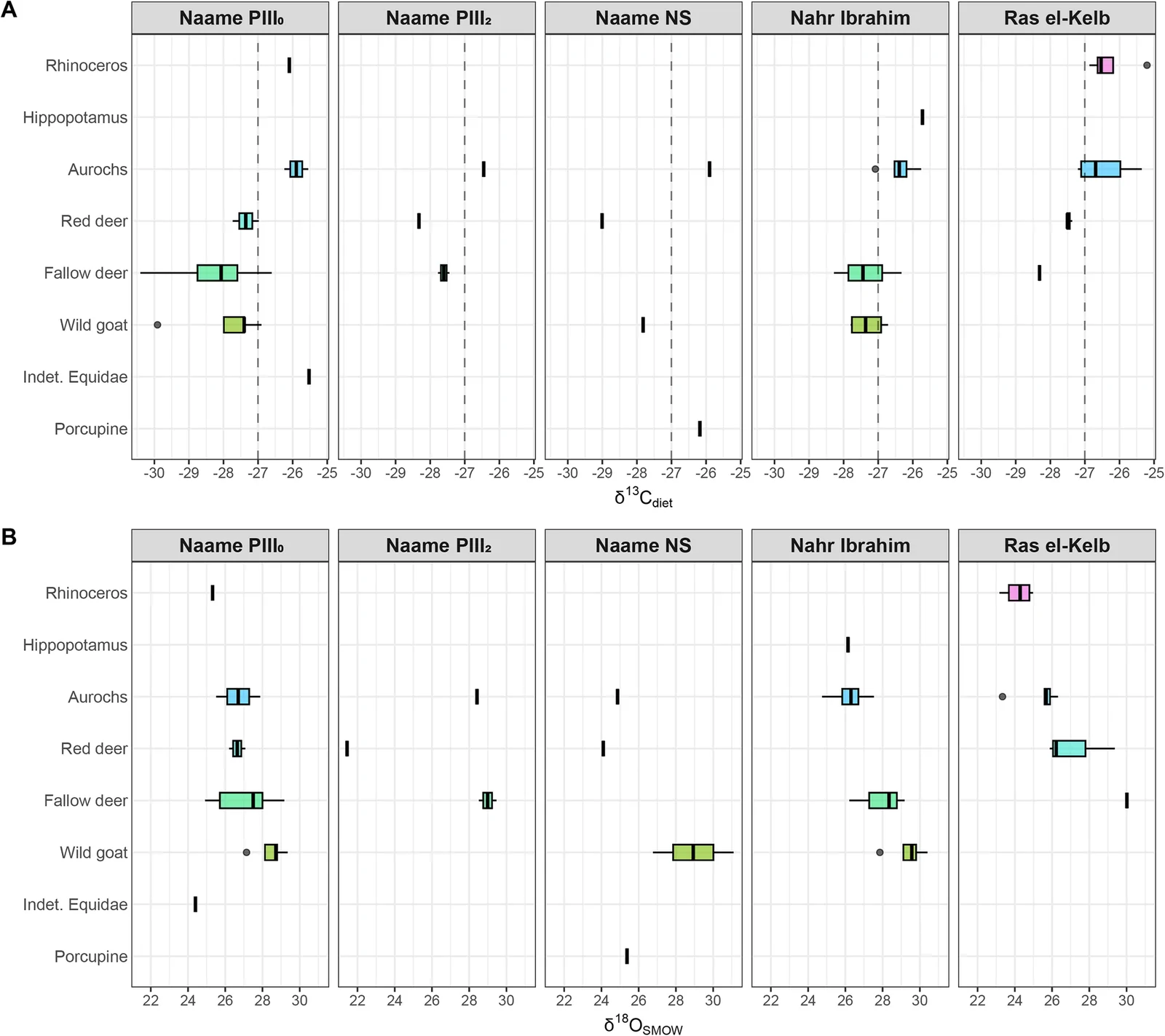
Mean carbon and oxygen isotope compositions of herbivore enamel across collections and species. **A** Mean δ^13^C_diet_ values (‰). The dashed line at −27‰ marks the approximate boundary between denser closed-canopy vegetation (values < – 27‰) and more open environments (values > – 27‰)^37^. **B** Mean δ^18^O_VSMOW_ values (‰). Box plots represent variability within each taxon and stratigraphic level (Naame NS =  *Niveau Supérieur*), illustrating interspecific and inter*-*site differences.

Across all sites, δ^13^C_diet_ values for the studied taxa range from −30.4‰ to −25.2‰ (Supplementary Table S2 and Fig. 2A), consistent with exclusive C3 plant consumption. However, the magnitude and pattern of value variation differ by site and taxon. At Naame (across levels), ungulates exhibit the overall widest δ^13^C_diet_ range (4.9‰), driven by the consistently low values of fallow deer and high values of aurochs relative to the same taxa in the other assemblages. By stratigraphy, herbivores from Naame - PIII_0_ (lower layer) and Naame *Niveau Supérieur* (upper layer) display similarly broad ranges, whereas the intermediate layer, i.e., Naame - PIII_2_, they show relatively narrower amplitudes. At Nahr Ibrahim, δ^13^C_diet_ values show limited amplitude (2.6‰), and ungulates occupy distinct positions with wild goat showing lower values, aurochs and hippopotamus more higher values, and fallow deer intermediate ones. At Ras el-Kelb, herbivore δ^13^C_diet_ values show moderate seasonal amplitude (3.3‰). Here, red deer exhibit the narrowest range in δ^13^C_diet_ values of any taxon at any site, whereas rhinoceros show the broadest niche breadth. Detailed species-site comparisons are provided in Supplementary Result S3.3.

### Mean δ^18^O isotopic data

Mean δ^18^O_VSMOW_ values vary substantially across collections (Supplementary Table S2 and Fig. 2B). The overall herbivore assemblage from Naame (across levels) exhibited the greatest range in δ^18^O enamel values compared to the other sites, spanning from −9.2‰ to +0.2‰, a range of 9.4‰, possibly reflecting, at least partly, its mixed chronology (MIS 5 and MIS 2). Nahr Ibrahim ungulates show the narrowest range (−6.0‰ to −0.5‰), while at Ras el-Kelb, taxa yield intermediate values ranging from −7.5‰ to −0.9‰. However, when restricting the comparison fallow deer and aurochs, which are present across all collections, δ^18^O ranges are comparable at Naame (4.5‰) and Nahr Ibrahim (4.3‰), whereas Ras el-Kelb exhibits the widest taxon-controlled δ^18^O range (6.4‰). Across sites, large-bodied obligate drinker ungulates, such as aurochs, hippopotamus, rhinoceros, and equids, show consistently lower values than wild goat and fallow deer (Supplementary Result S3.4).

Seasonal δ^18^O_VSMOW_ amplitudes inferred via inverse modeling (Supplementary Fig. S3 and Supplementary Table S5) show wild goat with the largest seasonal ranges (9.2–14.7‰, *n* = 4) across taxa, likely reflecting evaporative enrichment, whereas aurochs exhibit more moderate amplitudes (3.8–9.3‰, *n* = 5) compared to wild goat. Equus NME43 shows an anomalously large modeled amplitude (21.3‰), resulting from closely spaced isotopic peaks and troughs superimposed on a noisier profile, and is excluded from climatic interpretations.

### Relative isotopic niches

**Fig. 3:**
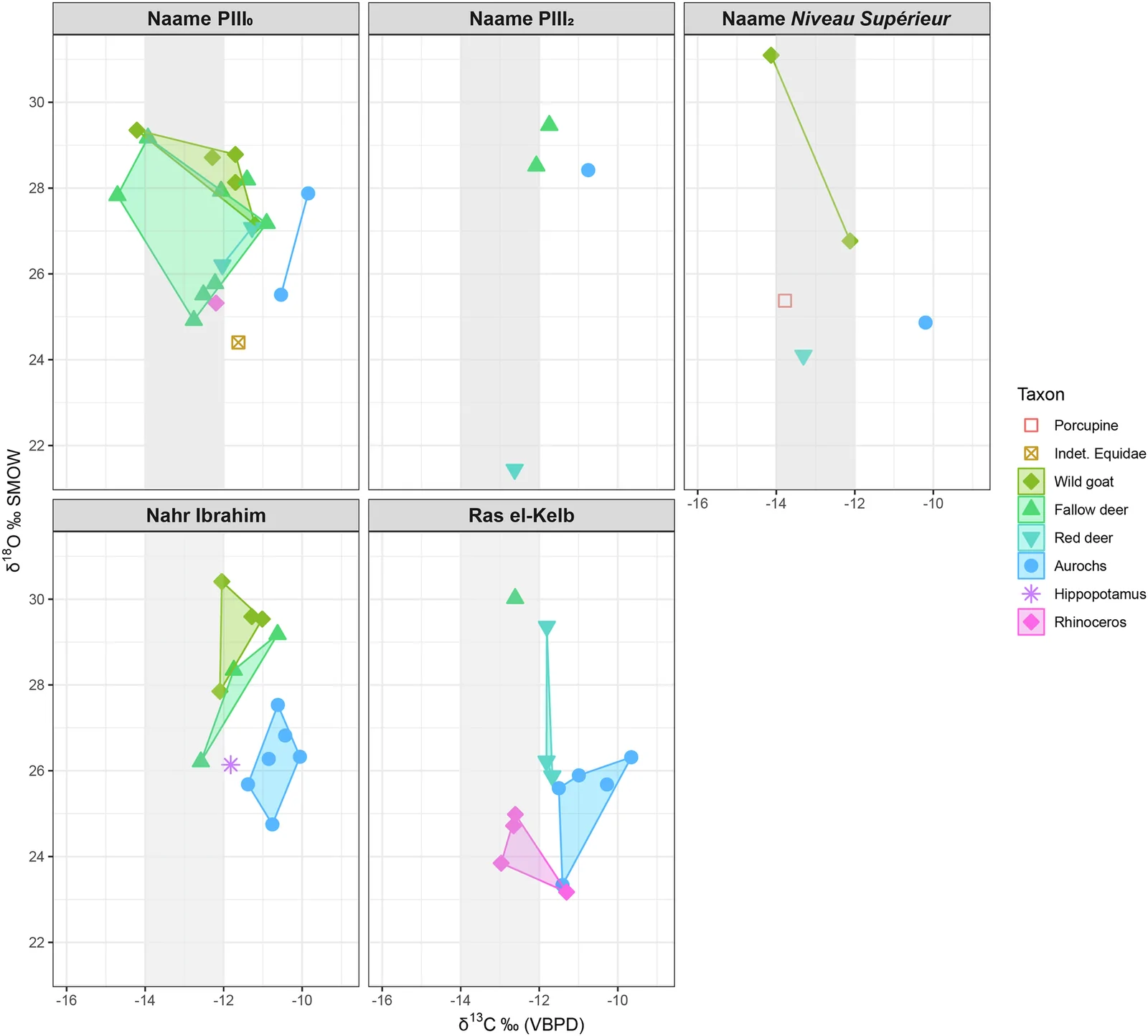
Biplots of mean enamel carbonate δ^13^C_VPDB_ and δ^18^O_VSMOW_ values illustrating the relative isotopic niches of taxa across sites. Each point represents the mean isotopic composition of an individual specimen, grouped by taxon and site. Grey vertical bands highlight the approximate range of δ^13^C values typical of C3-dominated open woodland environments^37^.

Combined δ^13^C_VPDB_-δ^18^O_VSMOW_ of mean ungulate enamel carbonate values illustrates clear differences in isotopic niche breadth across sites and levels (Fig. 3). At Naame - PIII_0_, herbivores display the largest isotopic niche breadth, particularly among fallow deer and wild goat, consistent with pronounced habitat heterogeneity. Aurochs cluster in elevated δ^13^C and intermediate δ^18^O space, indicative of open-habitat foraging and consumption of relatively ^18^O-depleted water. Sample sizes at Naame - PIII_2_ and Naame - *Niveau Supérieur* are too small to define robust niche patterns; however, intrasite comparison may suggests a contraction of the overall isotopic niche space in PIII_2_, followed by a renewed expansion in the *Niveau Supérieur*. Ungulates from Nahr Ibrahim exhibit a moderately constrained isospace. Wild goat, fallow deer, and aurochs form three discrete, non-overlapping convex hulls, with the hippopotamus specimen plotting outside of them, consistent with a well-structured community with clear ecological segregation and niche partitioning. Similarly, Ras el-Kelb ungulates shows distinct isotopic clustering among aurochs, red deer, rhinoceros, and fallow deer, with generally lower δ^18^O values compared to the other collections. Overall, isotopic niches remain distinct across species and sites, demonstrating long-term stability in herbivore niche partitioning over ~300 ka.

### Paleoprecipitation, temperature, and seasonal amplitude

**Fig. 4:**
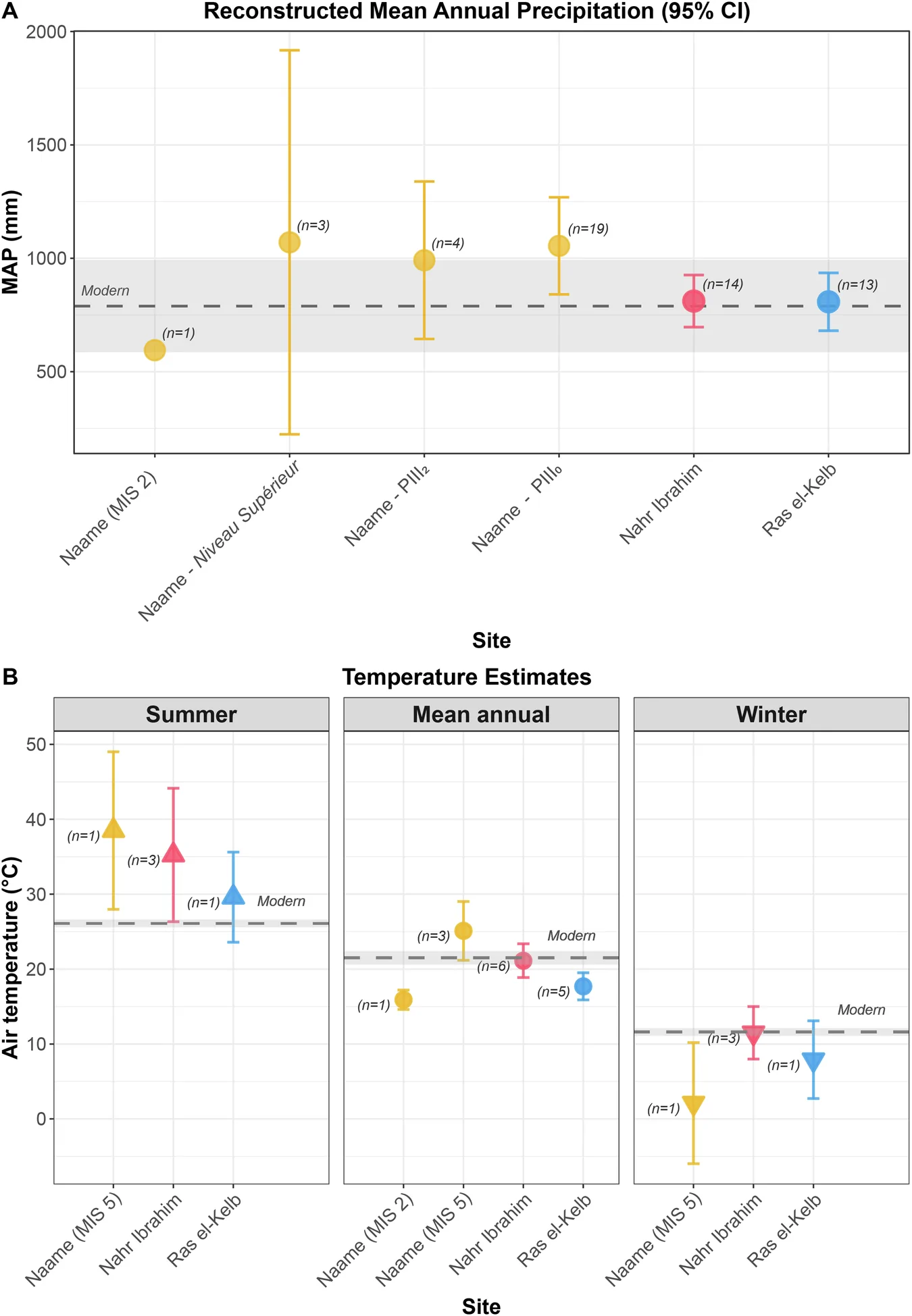
Reconstructed paleoclimatic temperature and precipitation estimates for the studied sites. **A** Mean annual precipitation (MAP, mm) with 95% confidence intervals and **B** reconstructed air temperature (°C) for summer, mean annual, and winter conditions. Dashed lines and shaded grey bands represent modern climatic baselines obtained from www.tutiempo.it (Beirut Airport station), with mean annual values and associated error (±1 SD) averaged from 2000 to 2020.

Mean annual precipitation (MAP) values reconstructed from δ^13^C_VPDB_ show consistently moderate rainfall across sites (Fig. 4A and Supplementary Table S7). Nahr Ibrahim and Ras el-Kelb yield mean MAP estimates of 811 mm (95% CI: ±115 mm) and 808 mm (95% CI: ±128 mm), respectively; values that fall within the range, but are slightly higher than modern mean precipitation at Beirut (789 mm; 95% CI: ±203.5 mm; *n* = 6). In contrast, Naame - PIII_2_ and PIII_0_ (MIS 5) (~991 mm; 95% CI: ±347 mm and ~1055 mm; 95% CI: ±214 mm) show substantially higher MAP values than today’s estimates for Beirut. The single MIS 2 aurochs from Naame - *Niveau Supérieur* yields markedly lower MAP (~595 mm) than the other estimated values and modern mean precipitation for Beirut.

Mean annual temperatures (MAT) estimated from aurochs δ^18^O values (Fig. 4B and Supplementary Table S7) deviate only modestly from modern coastal Lebanon, with broadly comparable seasonal structure across sites. Estimated MAT at Naame (MIS 5) is 25.1 ± 3.9 °C (*n* = 3), higher than the modern air temperature mean (21.5 ± 0.9 °C). Nahr Ibrahim recorded 21.1 ± 2.3 °C (*n* = 6), nearly identical to modern-day conditions, while Ras el-Kelb yielded 17.7 ± 1.8 °C (*n* = 5), about −3.8 °C below the present mean. Interestingly, the younger MIS 2 specimen from Naame -*Niveau Supérieur* produced a mean temperature of 15.9 ± 1.3 °C (*n* = 1), indicating −5.6 °C cooler conditions relative to today. Seasonal temperature reconstructions indicate substantially warmer summers and cooler winters than today, with pronounced chronological variability. Summer temperatures were highest at Naame during MIS 5, exceeding modern means by up to ~12 °C, followed by Nahr Ibrahim and Ras el-Kelb, whereas winter estimates ranged from near-modern conditions at Nahr Ibrahim to moderately cooler conditions at Ras el-Kelb. However, given the small sample sizes and since these estimates reflect inferred annual extrema of single individuals rather than multi-year seasonal averages, the observed differences should be interpreted as indicative patterns rather than statistically robust contrasts.

**Fig. 5:**
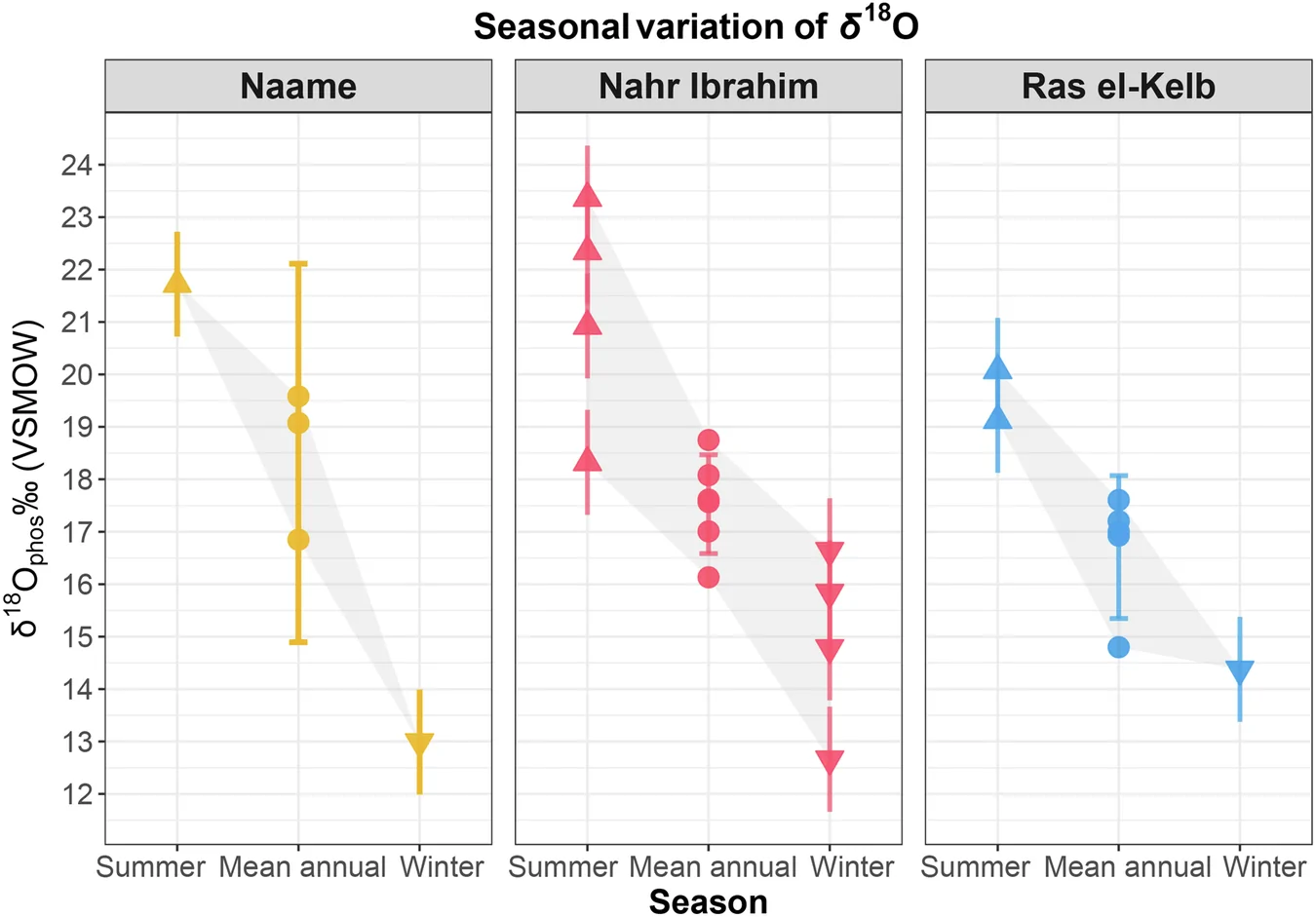
Seasonal variation in enamel phosphate δ^18^O_VSMOW_ across sites. Data points represent δ^18^O values and associated errors corresponding to summer peaks, winter troughs, and mean annual averages derived from sequentially sampled tooth enamel. Shaded areas illustrate the seasonal amplitude of δ^18^O variation within each site.

The corresponding seasonal amplitudes based on the derived phosphate δ^18^O_VSMOW_ values (see Supplementary Note S2.3) indicate broadly consistent seasonal contrasts across the three sites (Fig. 5), with mean summer-winter peak differences of ~8.7‰ at Naame (MIS 5), ~6.3‰ at Nahr Ibrahim, and ~5.2‰ at Ras el-Kelb, reflecting pronounced intra-annual temperature seasonality. Taking note of the small sample size, between-site differences are not statistically significant. Levene’s tests confirm homogeneity of variances (*p* > 0.3), and one-way ANOVAs for both summer (*F* = 0.61, *p* = 0.59) and winter (*F* = 0.54, *p* = 0.63) δ^18^O values reveal no significant chronological effect on seasonal amplitude.

### Relative aridity variation

To assess relative aridity, we compared δ^18^O_VSMOW_ values between evaporation-insensitive (EI) taxa, including aurochs, rhinoceros, hippopotamus, and the equid, and evaporation-sensitive (ES) species, represented here by wild goats. Under relatively arid conditions, greater evaporative enrichment of surface waters is expected to increase the δ^18^O offset between ES and EI species, whereas smaller offsets indicate more humid conditions.

**Fig. 6:**
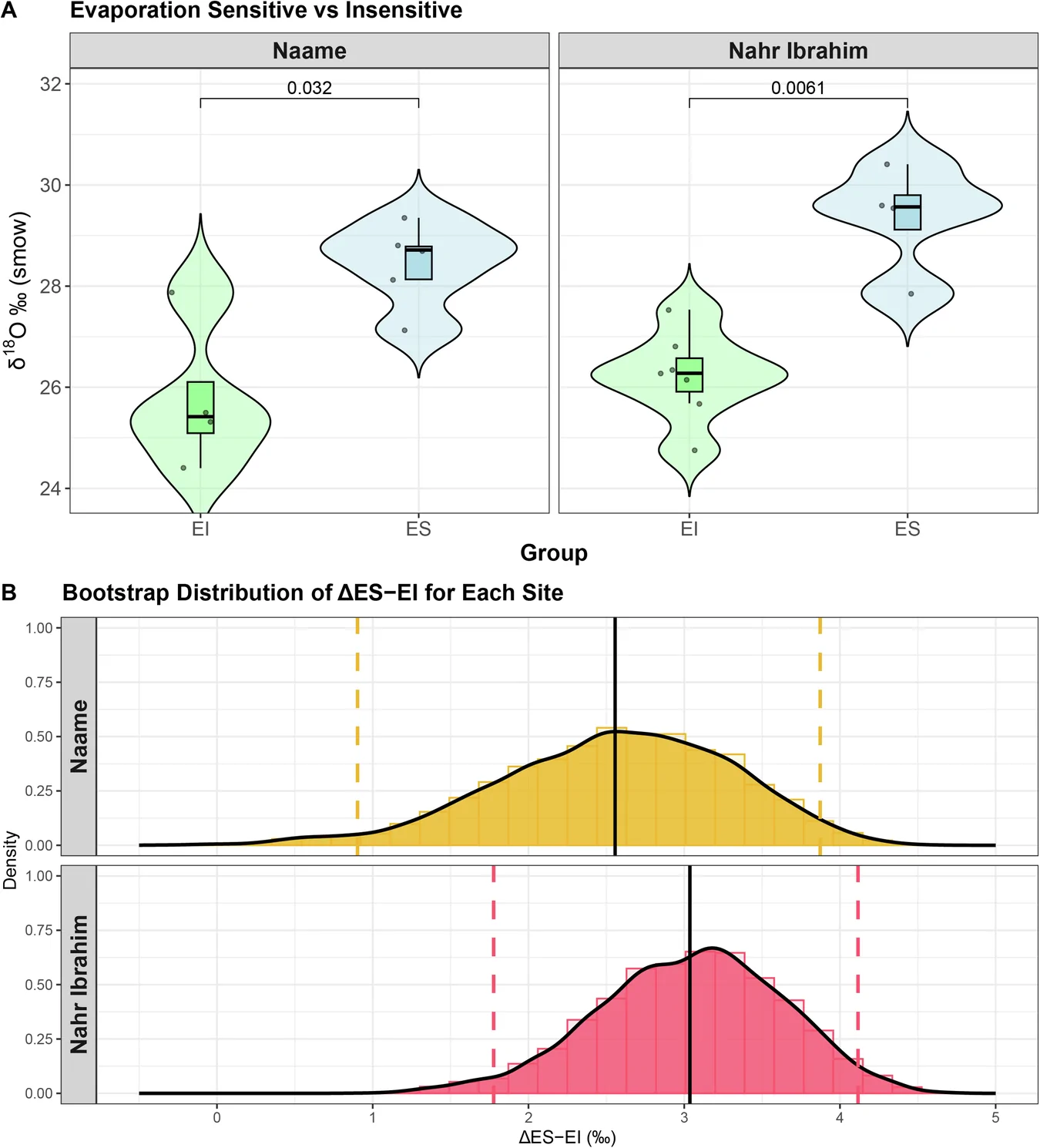
δ^18^O-based aridity index from evaporation-insensitive (EI) and evaporation-sensitive (ES) taxa. **A** δ^18^O_VSMOW_ values for EI and ES species at Naame (MIS 5) and Nahr Ibrahim (MIS 7). EI taxa consistently show lower δ^18^O _VSMOW_ than ES taxa, and differences are significant at both sites (Wilcoxon, *p* < 0.05). **B** Mean EI-ES differences (ΔES-EI) with standard deviations and bootstrap 95% confidence intervals. Larger EI-ES separation at Nahr Ibrahim indicates drier MIS 7 conditions compared to the MIS 5 at Naame.

The aridity index derived from δ^18^O_VSMOW_ differences between EI and ES taxa reveals a difference in relative humidity between Naame (levels PIII_2_ and PIII_0_) and Nahr Ibrahim (Fig. 6). At both sites, EI taxa consistently record lower δ^18^O values than the ES species, and effect sizes are large (Supplementary Result S3.5). The separation between EI and ES species is greatest at Nahr Ibrahim, indicating relatively drier conditions during MIS 7, whereas Naame shows smaller EI-ES separation consistent with more humid MIS 5 environments. Ras el-Kelb was not included in this analysis due to insufficient representation of both EI and ES taxa for a robust comparison. The Naame - *Niveau Supérieur* assemblage was likewise excluded given associated chronological uncertainty relative to the MIS 5 deposits.

## Discussion

Our multispecies isotopic data show that, despite major global climatic fluctuations between ~400 and 100 ka, the Lebanese coastal assemblages repeatedly record—across the sampled intervals—locally buffered ecohydrological settings with moderate hydroclimatic conditions and the persistence of C3‑dominated plant resources. This result does not seem to be due to time-averaging and potential mixing, which is unavoidable given the legacy nature of these collections, but see ref. ^29^. Importantly, the clear isotopic distinction of the MIS 2 specimen at Naame - *Niveau Supérieur* from those of the underlying MIS 5 assemblage, together with the coherent patterns of the isotopic values observed within each of the three sites, indicates that time averaging and chronological mixing do not obscure the primary environmental structure recorded in the data, even if they dampen short-term variability.

Thus, overall, our results show that productive habitats persisted in the central Levant even when neighboring regions experienced stronger aridity or temperature stress. Such sheltered habitats are relevant to questions of hominin dispersals through and occupation of the central Levant as they would have rendered this region a continuously, or at least a recurrently, habitable corridor during multiple climatic phases through sustaining ecological continuity. This, in turn, would have provided opportunities for hominin occupation, connectivity, and demographic processes operating at broader spatial scales.

### Vegetation structure and habitat heterogeneity

Across all three Lebanese assemblages, the δ^13^C_diet_ ranges of herbivores demonstrate consistent reliance on C3 vegetation^35,36^, yet their variability reveals nuanced differences in habitat structure. All Naame deposits shows similarly wide isotopic range, indicating a heterogeneous mosaic of closed-canopy patches, semi-open woodlands, and open scrublands^35,37^. Browsing taxa, such as fallow deer, red deer, and wild goat, frequently exploited shaded understory habitats, whereas aurochs, rhinoceros, and equids fed more intensively in open or mixed settings. Nahr Ibrahim and Ras el-Kelb show slightly narrower δ^13^C ranges, where even woodland-associated species do not show the strongly depleted values observed at Naame, suggesting limited opportunities for under dense canopy foraging at the older sites. These habitat interpretations are consistent with dental-wear analyses from the same assemblages^29^ and align with long-term regional palaeoenvironmental archives^20,22^, which document mixed woodlands and local variation in canopy density in Lebanon during the Middle and Late Pleistocene.

### Regional climate and seasonality

Herbivore δ^18^O profiles in all three collections mostly display regular sinusoidal cycles that closely track modern Mediterranean seasonality, characterized by cool-wet winters and warm-dry summers with roughly ~70% of annual rainfall falling between November and March^23^ (Fig. S5). This trend, visible in both raw and inverse-modeled profiles, suggests stable interannual hydroclimate regimes. Notably, Naame (MIS 5) and Nahr Ibrahim (MIS 7) show strong interspecific δ^18^O amplitudes reflecting strong seasonal contrasts during interglacials, while Ras el-Kelb (~400  ka) exhibits markedly damped amplitudes, implying attenuated seasonality during the formation of the assemblage. Seasonal or altitudinal migration provides an alternative mechanism that could influence intra-tooth δ^18^O variability. In sequential enamel records, migratory behavior is expected to dampen seasonal amplitudes by minimizing exposure to environmental extremes, whereas non-migratory ungulates typically exhibit larger intra-tooth ranges^31,38^. The amplitudes observed at Naame (in all levels) and Nahr Ibrahim (~2 to 5‰ across taxa) result in large ranges, and therefore do not indicate systematic seasonal transhumance during enamel formation. Although Ras el-Kelb shows reduced amplitudes (~1 to 2‰), the consistency of this attenuation across taxa more plausibly reflects locally moderated climatic seasonality rather than taxon-specific mobility.

Reconstructed MAT and MAP values refine the climatic patterns, and especially when employed in conjunction with the direct dating results. Ras el-Kelb shows slightly cooler temperatures to modern-day values but comparable precipitation estimates, more consistent with global conditions at glacial-interglacial transitions (e.g., MIS12/11, or MIS 11/10; Fig. 1) rather than full interglacial optima. Nahr Ibrahim closely matches modern precipitation and temperature patterns and correlates well with the relatively mild climate documented for the central Levant during early MIS 7 (ca. 243–222 ka, i.e., the overlapping time between the 1*σ* age range of 268–222 ka and the MIS7 boundaries 243–191 ka) from regional archives, such as the Yammoûneh δ^18^O and pollen records^20,22^. Interestingly, similar patterns for mild, humid Mediterranean environments during MIS 7 have also been suggested for the southern Levant from the microfaunal record at Misliya^13^, pointing to a broader regional climatic stability during this period. At Naame, the interglacial deposits PIII_0_ and PIII_2_ yield temperatures and precipitation consistently exceeding modern values, and tracking the well-established warm-humid peak of MIS 5 (i.e., around 126–120 ka, which is compatible within uncertainties with the direct dating results) in Lebanon documented in the Kanaan Cave stalagmite record^39^. This relatively uniform hydroclimatic conditions as inferred for this period for the central Levant seems to differ from records at several contemporaneous southern Levantine sites where isotopic and faunal proxies are suggestive of comparatively greater seasonal amplitude or interannual variability. At Nesher Ramla, δ^18^O values of aurochs from Unit III show higher means and larger seasonal amplitudes^40^, indicating greater aridity and interannual variability. By contrast, wild goat δ^18^O values from Qafzeh^16^ appear similar to those from Naame. Additional evidence from micromammal and herpetofaunal assemblages from Qafzeh, Rantis, Tabun B, and Tabun C reveals instead extreme interannual climatic fluctuations during MIS 5^16,41,42^. Although some of these differences may reflect variation in accumulation histories, analytical approaches, or temporal and spatial resolution, they nonetheless suggest stronger hydroclimatic fluctuations at several southern Levantine sites compared with the more buffered signal recorded along the Lebanese coast during MIS 5.

A single aurochs specimen from Naame - *Niveau Supérieur* (MIS 2) records markedly lower reconstructed temperatures and precipitation values relative to modern ones, and shows both the widest intra-tooth δ^18^O range and the most depleted δ^18^O value in our dataset. Although interpretation of a single specimen warrants caution, this isotopic signal aligns closely with regional speleothem and lacustrine archives documenting pronounced cold and dry conditions during the Last Glacial in Lebanon and the broader Levant (e.g., refs. ^22, 43, 44^). Its stark contrast with the underlying MIS 5 deposits, validates both the chronological placement of this specimen and the inferred severity of MIS 2 climatic deterioration in the central Levant^22^.

### Relative aridity and regional contrasts

Evaporation-sensitive vs. evaporation-insensitive taxa reveal clear differences in relative aridity between interglacials, with Naame (MIS 5) showing more humid conditions than Nahr Ibrahim (MIS 7). Comparable humidity reconstructions have been proposed for the southern Levant^16^, but their interpretation of arid-to-mesic habitats across MIS 5 at Qafzeh and a shift to humid MIS 4-3 conditions at Amud is problematic, as it contrasts with the well-established and widely recognized relationship between δ^18^O enrichment and evaporative water loss under arid conditions (e.g., refs. ^16, 31, 45–47^). When compared regionally, Amud wild goats show the highest δ^18^O_VSMOW_ values, indicating more arid conditions, whereas Qafzeh wild goats isotopic signal overlaps with that of Naame and Nahr Ibrahim (Supplementary Fig. S6). This suggests generally more humid MIS 5 conditions at Qafzeh and drier MIS 4-3 conditions at Amud, calling for a revision of prior interpretations and a redefinition of our understanding of the ecological settings encountered by Neanderthals and anatomically modern humans in the southern Levant.

### Isotopic niches and herbivore paleoecology

Across Ras el-Kelb, Nahr Ibrahim, and Naame (in all levels), herbivore communities maintain clearly differentiated isotopic niche partitioning across multiple glacial-interglacial cycles. Cervids remain the most δ^13^C-depleted species, reflecting sustained year-round reliance on shaded understory browse in humid C3 woodlands. Notably, Persian fallow deer and red deer from the central Levant exhibit markedly lower δ^13^C values than Middle Pleistocene European populations (e.g., refs. ^48–50^), reflecting the exceptional density and persistence of the Pleistocene Lebanese coastal woodlands. Wild goats consistently display moderately higher δ^13^C and elevated δ^18^O values compared to cervids, suggesting mixed feeding across patchy woodland and open scrub with reduced reliance on drinking water. The broader δ^13^C range at Naame contrasts with the constrained C3 diets of MIS 4-3 Amud wild goats and the partly C4-influenced diet of wild goat from the MIS 5 lower unit of Qafzeh^16^, indicating access to denser and more heterogeneous woodland habitats in the central Levant. Aurochs consistently show the most enriched δ^13^C values, and wide intra-tooth δ^18^O and δ^13^C amplitudes, indicating flexible and seasonal mixed feeding across open woodland, shrubland, and water-stressed C3 grasses. Their δ^13^C variability exceeds that of MIS 5 aurochs from Nesher Ramla^40^, supporting greater habitat heterogeneity in the central Levant, but they are generally slightly more enriched than MIS 5/7 large bovines from south-eastern France and Central Italy^49,51^, consistent with greater exposure to open environments in the eastern Mediterranean. Rhinoceros (*Stephanorhinus hemitoechus*) at Ras el-Kelb occupies intermediate δ^13^C values spanning from closed to open C3 habitats, and a narrower δ^13^C range than central European populations^48^, aligning more with the closely related *S. kirchbergensis* from central Italian MIS 11-7 sites^50,52^. The hippopotamus from Nahr Ibrahim displays relatively lower δ^18^O values consistent with an aquatic lifestyle but higher δ^13^C values than MIS 7 *Hippopotamus amphibius* from central Italy^51^, reflecting the inclusion of more open C3 vegetation in the diet of the Levantine specimen. The MIS 5 Naame equid shows enriched C3 open environment signatures typical of Middle Pleistocene and Late Pleistocene equids across the central Mediterranean and central Europe (e.g., refs. ^48–50^).

Collectively, these species-specific isotopic patterns suggest that between ~400 and ~100 ka the Lebanese coast more closely resembled a temperate European-Mediterranean mosaic of humid C3 woodlands, riparian corridors, and open scrublands, which supported spatial and dietary partitioning of multiple sympatric herbivores. This picture differs from records of several southern Levantine sites that generally indicate more open environments and larger hydroclimatic fluctuations during the same time intervals, even if these fluctuations might have been of limited amplitude in some locations and thus might have been expressed within a spatial environmental mosaic, e.g., refs. ^53, 54^.

### Implications for hominin dispersal and occupation

The isotopic signatures record environmental conditions experienced by the sampled animals during tooth formation, thus providing intra-annual resolution. Because the isotopic signatures in this case derive directly from zooarchaeological assemblages, they can inform landscape-scale environmental reconstructions associated with episodes of hominin presence^29^. Middle Pleistocene human populations at Ras el-Kelb (~400 ka) and Nahr Ibrahim (~240 ka)—the latter broadly contemporaneous with the early *Homo sapiens* remains from Misliya^55^—inhabited a stable, heterogeneously resource-rich landscape structure. At Naame, foragers occupied similarly buffered environments during the last interglacial (MIS 5), a period characterized by climatic amelioration and increased ecological connectivity also across the Saharo-Arabian Desert belt^7,56^. These conditions likely facilitated major early expansions of anatomically modern human populations out of Africa, as documented at Qafzeh and Skhul^57–59^, while also coinciding with the presence of Neanderthal-like archaic hominins in the Levant, attested at Nesher-Ramla^60^. Comparable patterns of ecological reliability are evident later at Ksar’Akil, where independent isotopic and archaeological evidence demonstrated sustained, year-round coastal resource exploitation during the Upper Paleolithic^19^, another critical period likewise associated with renewed dispersal dynamics and demographic expansions of anatomically modern humans. Taken together, the ecological predictability of the central Levant likely facilitated repeated settlement and mobility for incoming Eurasian populations, while also supporting expansions of *Homo sapiens* dispersing from Africa.

By integrating high-resolution enamel isotope records with independent regional archives, this study suggests that the coastal central Levant formed a biogeographically distinct corridor relative to southern regions, and functioned as a Mediterranean refugium whose long-term environmental stability influenced the timing and geography of Pleistocene hominin adaptation and dispersals between continents.

## Methods

### Materials, sampling, and pre-treatment

This study analyzed stable carbon and oxygen isotope compositions of tooth enamel carbonates of herbivores from three Pleistocene archaeopaleontological deposits in Lebanon chronologically spaced at approximately 150 ka intervals to reconstruct local paleoenvironmental conditions and infer long-term environmental, climatic, and ecological trends in the Central Levant.

A total of 58 teeth were selected from legacy collections originating from three sites across the Lebanese coast: Ras el-Kelb (*n* = 13) and Nahr Ibrahim (*n* = 14) collections, excavated by Jesuit Father Zumoffen nearly 130 years ago^61^, and Naame (across levels, *n* = 31), excavated by Jesuit Father Fleisch approximately 60 years ago^62^. The sites are located within 15–30 km of each other and share similar environmental settings, including comparable elevations and distances from the present-day shoreline, and proximity to an important river system (Fig. 1). As these are all legacy collections lacking chronological control, five of these teeth (3 from Naame and one each from Nahr Ibrahim and Ras el-Kelb) were recently directly dated using combined U-series/ESR methods. The results placed Ras el-Kelb as the oldest collection, dating to 402 ± 46 ka (1*σ*) (Middle Pleistocene); Nahr Ibrahim dates to 245 ± 23 ka (1*σ*); Naame main archaeological layers (PIII_0_ and *Niveau Supérieur*) fall mostly within MIS 5 (~130–71 ka, with a finite age of 107 ± 13 ka − 1*σ*), while another tooth from Naame - *Niveau Supérieur* returned a younger age, dating to MIS 2 (~18 ka)^29^. (Supplementary Table S1).

Species and skeletal element identifications were conducted on these collections^29^ using the comparative zooarchaeological reference collection at the University of Tübingen. Taphonomic analysis was also conducted^29^. At Naame (across levels), cut marks, systematic breakage for marrow extraction, minimal carnivore activity, and skeletal representation all indicate that hominins were the primary accumulators and processors of fauna, meaning the isotopic values reliably reflect prey processed on site. At Nahr Ibrahim, taphonomic signatures likewise show that humans were the dominant accumulating agents, with cut-marked and freshly broken bones and very rare carnivore damage supporting the interpretation that the isotopic data reflect hominin-hunted prey. At Ras el-Kelb, however, the taphonomic agency behind the accumulation is less secure: evidence of human presence (burning, fresh fractures, and associations with lithic tools) co-occurs with occasional carnivore modification, and the limited contextual information further complicates interpretation. Consequently, the isotopic values are best viewed as reflecting environmental conditions during periods of human presence in the landscape, while acknowledging the possibility of non-anthropogenic contributions to the assemblage. See Supplementary S1 for further details on the archaeological background. Details on faunal composition, taphonomic analysis, and chronology of these assemblages are provided in Russo et al.^29^.

The 58 teeth selected represented eight herbivore species: aurochs (*Bos primigenius*, *n* = 15), wild goat (*Capra aegagrus*, *n* = 11), red deer (*Cervus elaphus*, *n* = 9), Persian fallow deer (*Dama mesopotamica*, *n* = 15), narrow-nosed rhinoceros (*Stephanorhinus hemitoechus*, *n* = 5), hippopotamus (*Hippopotamus amphibius*, *n* = 1), equids (*Equus* sp., *n* = 1), and porcupine (*Hystrix* sp., *n* = 1). All sampling was conducted at the Senckenberg Centre for Human Evolution and Palaeoenvironment and Department of Geosciences, University of Tübingen. Before sampling, the tooth surface was mechanically cleaned using a Dremel drilling device equipped with a cylindrical diamond bit (3 mm in diameter) to remove adhering sediment, potential contaminants, and the cementum layer overlying the enamel. Whenever possible, enamel was sampled in horizontal bands spaced 2–4 mm apart, starting from the tip of the occlusal cusp (where enamel formation begins) down to the enamel-root junction (ERJ), collecting approximately 12–20 mg of enamel powder per band. This sequential sampling aimed to record variations along the enamel growth axis. When sequential sampling was not possible due to poor preservation of the tooth, enamel was collected in bulk. In these cases, care was taken to sample a similar amount of enamel powder along the longitudinal axis of the tooth surface to obtain a representative mean isotopic value. When preservation allowed, dentine samples ranging from 18 to 20 mg were also taken from the root.

The pretreatment followed the protocol outlined by Bocherens^63^, to eliminate potential diagenetic contamination and isolate biogenic carbonate. The first step involved the removal of organic material by treating each sample with 1.35 ml of a 2.5% sodium hypochlorite (NaOCl) solution. The samples were continuously mixed using a vortex and left in solution for 24 h on a shaker. Due to their higher organic content, dentine samples underwent two consecutive treatments to ensure complete removal. After 24 h, the NaOCl solution was removed, and the samples were rinsed three times with Milli-Q distilled water. Between each rinse, the samples were centrifuged at 3500 rpm for 3 min to separate the liquid and solid fractions. Once organic removal was complete, a secondary carbonate elimination step was performed by treating the samples with 1.35 ml of a 1 M buffered acetic acid (CH_3_COOH) solution^64–66^. The solution was homogenized using a vortex and left on a shaker for other 24 h. Following this treatment, the samples were again rinsed three times with Milli-Q water and centrifuged to ensure complete acid removal^65^. Finally, the pre-treated samples were placed in an oven at 35 °C for 72 h to remove residual moisture before isotope analysis.

The yield of pretreated enamel was approximately 2.5–3 mg. Each sample was then reacted with 99% phosphoric acid (H_3_PO_4_) for 4 h at 70 °C using a MultiFlow-Geo device interfaced with an Elementar IsoPrime 100 isotope ratio mass spectrometer (IRMS). Calibration was performed using two international standards—IAEA-603 (δ^13^C: +2.46‰; δ^18^O: −2.37‰) and NBS-18 (δ^13^C: −5.01‰; δ^18^O: −23.20‰)—and two untreated in-house enamel standards: elephant (δ^13^C: −10.55‰; δ^18^O: +1.80‰) and Hippo (δ^13^C: −3.80‰; δ^18^O: −2.10‰). Analytical precision was better than ±0.1‰ for carbon and ±0.2‰ for oxygen isotopic measurements. Final isotope ratios (^13^C/^12^C and ^18^O/^16^O) are reported relative to the VPDB standard in delta (δ) notation per mil (‰). δ^18^O values were normalized from the VPDB to the VSMOW scale following Coplen et al.^67^. Two in-house reference standards (fossil hippopotamus and elephant enamel from a paleontological site in Chad) included in the analytical dataset were subjected to the same pretreatment protocol as the archaeological samples. These standards yielded carbonate weight percentages ranging from 4.1 to 6.5%, providing an internal reference for comparison. Diagenetic alteration and contamination were assessed by observing the carbonate (CO_3_)^65^ and by examining its co-variation across sites and taxa. For each site, we calculated sample size, mean, standard deviation to assess dispersion and visually identify potential diagenetic outliers. Statistical differences among groups were tested using the non-parametric Kruskal–Wallis test. When significant (*p* < 0.05), post hoc pairwise comparisons were performed with Dunn’s test, applying Bonferroni-adjusted *p*-values to support the interpretation of potential outliers.

### Reconstruction of diet and precipitation

To reconstruct dietary patterns, ecological niches, and paleoenvironments (see Supplementary S2), we corrected δ^13^C values from tooth enamel carbonates (δ^13^C _VPDB_) for the isotopic enrichment between diet and bioapatite (ε*), following species-specific digestive physiologies. Enrichment factors were primarily derived from Cerling et al.^68^, where fixed ε* values for different fermentative groups, thereby minimizing uncertainties related to fossil body-mass reconstructions. Plant δ^13^C values vary predictably according to their photosynthetic pathway: C3 plants (trees, shrubs, forbs, and cool-season grasses) typically range from −36‰ to −22‰, whereas C4 plants (warm-season grasses and sedges) range from −17‰ to −9‰ due to reduced ^13^C discrimination during fixation^69,70^. These distinct isotopic end-members provide a robust baseline for interpreting herbivore δ^13^C values and assessing ecological partitioning, particularly in distinguishing browsing in C3-dominated woodlands from increased use of C4-rich open grasslands. Therefore, after accounting for enamel-diet enrichment, the resulting δ^13^C_diet_ values were used to infer vegetation structure, with values > – 27‰ indicating woodland-mesic, C3 grassland, and parklands, and values < – 27‰ suggesting denser, closed-canopy environments^35,37^.

MAP was estimated from δ^13^C_diet_-derived Δ^13^C_pl_ values following the regression model of Rey et al.^71^ and the approach of Lécuyer et al.^72^, assuming an average atmospheric δ^13^C_atm_ of −6.8‰^73,74^. Because both δ^13^C_atm_ and vegetation-MAP relationships vary through time, our MAP values should be regarded as broad, first-order estimates used mainly to compare relative precipitation between sites and levels rather than as precise absolute reconstructions. Our MAP values were cross-validated against modern precipitation data (Beirut Airport, 2000–2020), but we stress that further validation should come through the comparison with other paleoenvironmental proxies. See Supplementary Note S2.2 for equations and additional methodological details.

### Inverse modeling, seasonal temperature, and aridity

To obtain more precise estimates of seasonal temperature variation, we applied an inverse modeling approach following Passey et al.^75^ to δ^18^O data derived from sequential sampling of bovid and equid tooth enamel, which typically forms sinusoidal patterns along the sequence of tooth growth. These patterns can be affected by time-averaging and amplitude damping caused by enamel mineralization dynamics and sampling resolution, potentially biasing the estimation of seasonal amplitude. To address these issues, we followed the method of Pederzani et al.^33^ to extract summer peak and winter trough values from the inverse-modeled δ^18^O curves, implementing the same modeling procedure as outlined in their open-access repository (https://github.com/scpederzani/Oxygen_Inverse_Model). Peak and trough values were first identified by visual inspection of the unmodeled δ^18^O curves and subsequently processed through inverse modeling to obtain corrected summer and winter δ^18^O values (Supplementary Fig. S3). All model parameters are provided in Supplementary Note S2.3, and the codes and datasets used in this study are available at https://github.com/gabrieleru91-coder/Oxygen-and-Carbon-stable-Isotope-analysis-Lebanon.

Air temperature estimates were derived from the empirical relationships between enamel phosphate (δ^18^O_phos_) to drinking water (δ^18^O_dw_), and precipitation (δ^18^O_precip_) to air temperature following Pryor et al.^76^. Seasonal temperature peaks were estimated from inverse-modeled δ^18^O values of aurochs (*B. primigenius*), while mean annual temperatures were calculated using unmodeled δ^18^O mean values following Pederzani et al.^33^. Aurochs were selected for paleotemperature estimation as large-bodied obligate drinkers with sufficient sample size per site, ensuring reliable isotopic representation. The single equid tooth was excluded from the final interpretation due to (1) incompleteness, lacking the proximal portion necessary to determine the full growth sequence, (2) relatively flat δ^18^O profiles leading to higher uncertainty, and (3) the inverse model’s inability to account for the deceleration of tooth growth and mineralization known in equids^33,34^. Caprine teeth were not included in this analysis because, at the moment of writing, no modern δ^18^O_phos_ datasets for caprine enamel are available for calibration. For aurochs, we used published data on large bovids δ^18^O_phos_ and δ^18^O_dw_^75^ and modern temperature and δ^18^O_precip_ data from meteorological stations in the central Levant. The two-step regression was implemented using the codes provided by Pederzani et al.^33^ (https://github.com/scpederzani/Isotope_Temperature_Calibration). A full description of the calculations and parameters used in this study is presented in Supplementary Note S2.4, and all datasets and codes required to reproduce this analysis are available in the project’s online repository (https://github.com/gabrieleru91-coder/Oxygen-and-Carbon-stable-Isotope-analysis-Lebanon).

The difference in tooth enamel δ^18^O values between obligate drinkers (evaporation-insensitive, EI) and non-obligate drinkers (evaporation-sensitive, ES) reflects evaporation-driven ^18^O enrichment between source and evaporated water, which becomes more pronounced under arid conditions^45^, providing a proxy for local moisture variability over time. Following this principle, we first identified EI and ES taxa in our sample, ensuring that δ^18^O values reliably reflected evaporation and meteoric water signals. Critical sources of uncertainty, including contemporaneity of specimens, consistency of water sources, adequate sample size, and the absence of diagenetic alteration, were evaluated according to the recommendations of Faith et al.^47^. The analysis was implemented following the methodology outlined in Luyt et al.^77^. Details regarding the estimation of uncertainties are provided in Supplementary Note S2.5. All codes and datasets used in this analysis are publicly available in the project repository (https://github.com/gabrieleru91-coder/Oxygen-and-Carbon-stable-Isotope-analysis-Lebanon).

### Cross-correlation, isotopic niche, and statistical analysis

Potential seasonal dietary patterns were examined through the correlation between tooth enamel δ^13^C and δ^18^O time series values within each specimen, using the cross-correlation function (“ccf”) in R Studio. A correlation coefficient (*R*) greater than 0.5 was taken to indicate a strong relationship, whereas values below this threshold were considered weak correlations.

Variation in δ^13^C and δ^18^O values and the corresponding ecological breadth of species were assessed by plotting individual mean values within a two-dimensional isotopic niche space, or isospace^78,79^. Convex hulls were used to illustrate the total isotopic range of each species, representing the overall extent of dietary and environmental variation within the samples. Because isotope ratios in tooth enamel closely reflect diet and drinking water sources, these plots provide a visual framework for comparing relative ecological niches among herbivore communities. By grouping individuals by collection and, in the case of Naame, by levels, this approach allows for the assessment of temporal and spatial changes in faunal dietary and ecological patterns through time. Gray boxes were added for visualization purposes on the δ^13^C axis to highlight suggested boundaries between closed-canopy forests, open woodlands, and open grasslands.

All statistical analyses were performed in RStudio (version 4.3.1) with a significance threshold of *p* = 0.05. Detailed descriptions of all the analyses and modeling procedures are provided in Supplementary Note S2. Associated codes, packages, and data used in this study are available at https://github.com/gabrieleru91-coder/Oxygen-and-Carbon-stable-Isotope-analysis-Lebanon.

### Reporting summary

Further information on research design is available in the Nature Portfolio Reporting Summary linked to this article.

## Supplementary information

Supplementary Information

Reporting Summary

Transparent Peer Review File

## Data Availability

All datasets generated and/or analysed during the current study are publicly available in the public project GitHub repository: https://github.com/gabrieleru91-coder/Oxygen-and-Carbon-stable-Isotope-analysis-Lebanon. The repository includes the oxygen and carbon stable isotope datasets, associated sample metadata, and processed data files used for the analyses presented in this study. No restrictions apply to data access.

## Code availability

All R scripts used to process, analyse and visualize the data are publicly available in the same project repository at https://github.com/gabrieleru91-coder/Oxygen-and-Carbon-stable-Isotope-analysis-Lebanon.
