# Supplementary material for: Long-term ecological stability in the Pleistocene central Levantine corridor revealed by enamel isotopes from Lebanon
Source: Commun Earth Environ. 2026 Sep 14;7(1):705. doi: 10.1038/s43247-026-03925-x (PMC13574713; doi:10.1038/s43247-026-03925-x)
Supplement: Supplementary file 1 [file 43247_2026_3925_MOESM2_ESM.pdf]

nature portfolio

Corresponding author(s): Gabriele Russo

Last updated by author(s): Dec 21, 2025

Reporting Summary

Nature Portfolio wishes to improve the reproducibility of the work that we publish. This form provides structure for consistency and transparency in reporting. For further information on Nature Portfolio policies, see our Editorial Policies and the Editorial Policy Checklist.

Statistics

For all statistical analyses, confirm that the following items are present in the figure legend, table legend, main text, or Methods section.

n/a	Confirmed	
☐	☒	The exact sample size (*n*) for each experimental group/condition, given as a discrete number and unit of measurement
☐	☒	A statement on whether measurements were taken from distinct samples or whether the same sample was measured repeatedly
☐	☒	The statistical test(s) used AND whether they are one- or two-sided<br>*Only common tests should be described solely by name; describe more complex techniques in the Methods section.*
☒	☐	A description of all covariates tested
☐	☒	A description of any assumptions or corrections, such as tests of normality and adjustment for multiple comparisons
☐	☒	A full description of the statistical parameters including central tendency (e.g. means) or other basic estimates (e.g. regression coefficient) AND variation (e.g. standard deviation) or associated estimates of uncertainty (e.g. confidence intervals)
☐	☒	For null hypothesis testing, the test statistic (e.g. F, t, r) with confidence intervals, effect sizes, degrees of freedom and P value noted<br>*Give P values as exact values whenever suitable.*
☒	☐	For Bayesian analysis, information on the choice of priors and Markov chain Monte Carlo settings
☒	☐	For hierarchical and complex designs, identification of the appropriate level for tests and full reporting of outcomes
☐	☒	Estimates of effect sizes (e.g. Cohen's *d*, Pearson's *r*), indicating how they were calculated

Our web collection on statistics for biologists contains articles on many of the points above.

Software and code

Policy information about availability of computer code

Data collection	*Provide a description of all commercial, open source and custom code used to collect the data in this study, specifying the version used OR state that no software was used.*
Data analysis	Data analysis was conducted using R version 4.3.1. All analysis code is available at https://github.com/gabrieleru91-coder/Oxygen-and-Carbon-stable-Isotope-analysis-Lebanon

For manuscripts utilizing custom algorithms or software that are central to the research but not yet described in published literature, software must be made available to editors and reviewers. We strongly encourage code deposition in a community repository (e.g. GitHub). See the Nature Portfolio guidelines for submitting code & software for further information.

Data

Policy information about availability of data

All manuscripts must include a data availability statement. This statement should provide the following information, where applicable:

- Accession codes, unique identifiers, or web links for publicly available datasets
- A description of any restrictions on data availability
- For clinical datasets or third party data, please ensure that the statement adheres to our policy

All data generated for this study, including measurements and data analysis results are available at https://github.com/gabrieleru91-coder/Oxygen-and-Carbon-stable-Isotope-analysis-Lebanon

Research involving human participants, their data, or biological material

Policy information about studies with human participants or human data. See also policy information about sex, gender (identity/presentation), and sexual orientation and race, ethnicity and racism.

Reporting on sex and gender	NA
Reporting on race, ethnicity, or other socially relevant groupings	NA
Population characteristics	NA
Recruitment	NA
Ethics oversight	NA

Note that full information on the approval of the study protocol must also be provided in the manuscript.

Field-specific reporting

Please select the one below that is the best fit for your research. If you are not sure, read the appropriate sections before making your selection.

☐ Life sciences ☐ Behavioural & social sciences ☒ Ecological, evolutionary & environmental sciences

For a reference copy of the document with all sections, see nature.com/documents/nr-reporting-summary-flat.pdf

Ecological, evolutionary & environmental sciences study design

All studies must disclose on these points even when the disclosure is negative.

Study description	Multi-species stable isotope analyses ($\delta^{18}O$, $\delta^{13}C$) of 58 sequentially and bulk-sampled herbivore teeth from three Lebanese Pleistocene sites were used to reconstruct climate, seasonality, aridity, mobility, and feeding ecology between ~400 and 100 ka.
Research sample	The study analyzes enamel and dentine from large mammal herbivores (primarily aurochs, fallow deer, red deer, and rhinoceros) from legacy faunal collections of Naame, Nahr Ibrahim, and Ras el-Kelb stored at the Museum of Lebanese Prehistory in Beirut. These taxa were selected to represent local ungulate communities and regional paleoenvironmental conditions.
Sampling strategy	Sequential intra-tooth sampling targeted seasonal isotopic variation, while bulk enamel samples were used for inter-taxonomic and inter-site comparisons. Sample sizes were constrained by preservation and availability; no formal power analysis was performed, consistent with comparable paleo-isotopic studies.
Data collection	Tooth enamel and dentine were sampled using established protocols. Stable isotope measurements were conducted using a MultiFlow-Geo device interfaced with an Elementar IsoPrime 100 isotope ratio mass spectrometer (IRMS) following standard preparation and quality-control procedures. Analytical methods and instrumentation are detailed in the main article and supplementary Information.
Timing and spatial scale	Data collection was carried out during the first half of 2024. Samples derive from stratified Pleistocene deposits dated by ESR/U-series and associated chronologies, spanning ?400.000 to ?100.000 years. The spatial scale encompasses three coastal Lebanese sites at a short distance from each other (ca. 30 km) within the central Levantine corridor.
Data exclusions	Individual isotopic measurements failing predefined analytical or quality-control criteria were excluded. Outliers in intra-tooth $\delta^{18}O$ series were removed where statistically anomalous relative to fitted sinousoidal trends.
Reproducibility	Replicate isotope measurements were performed on subsets of samples to assess analytical precision. All data, statistical analyses, and code used for modeling and figure generation are provided in the Supplementary Information and associated data repositories.
Randomization	NA
Blinding	NA

Did the study involve field work? ☐ Yes ☒ No

Reporting for specific materials, systems and methods

We require information from authors about some types of materials, experimental systems and methods used in many studies. Here, indicate whether each material, system or method listed is relevant to your study. If you are not sure if a list item applies to your research, read the appropriate section before selecting a response.

Materials & experimental systems

n/a	Involved in the study
☒	☐ Antibodies
☒	☐ Eukaryotic cell lines
☐	☒ Palaeontology and archaeology
☒	☐ Animals and other organisms
☒	☐ Clinical data
☒	☐ Dual use research of concern
☒	☐ Plants

Methods

n/a	Involved in the study
☒	☐ ChIP-seq
☒	☐ Flow cytometry
☒	☐ MRI-based neuroimaging

Palaeontology and Archaeology

Specimen provenance: Research and exportation permits were granted by the Directorate General of Antiquities (DGA) and the Museum of Lebanese Prehistory in Beirut, Lebanon, in accordance with national law. Specimens derive from historical archeological colletions stored at the Museum.

Specimen deposition: Museum of Lebanese Prehistory (Saint Joseph University of Beirut), Lebanon

Dating methods: NA

☐ Tick this box to confirm that the raw and calibrated dates are available in the paper or in Supplementary Information.

Ethics oversight: No ethical approval was required for this study. All analyses were conducted on existing, curated archaeological faunal collections housed at the Museum of Lebanese Prehistory (Saint Joseph University of Beirut). All research followed the museum's policies for handling, sampling, and documenting heritage materials.

Note that full information on the approval of the study protocol must also be provided in the manuscript.

Plants

Seed stocks: NA

Novel plant genotypes: NA

Authentication: NA
