# Supplementary material for: Long-term ecological stability in the Pleistocene central Levantine corridor revealed by enamel isotopes from Lebanon
Source: Commun Earth Environ. 2026 Sep 14;7(1):705. doi: 10.1038/s43247-026-03925-x (PMC13574713; doi:10.1038/s43247-026-03925-x)
Supplement: Supplementary file 2 [file 43247_2026_3925_MOESM3_ESM.pdf]

nature portfolio

Peer Review File

Long-term ecological stability in the Pleistocene central Levantine corridor revealed by enamel isotopes from Lebanon

Corresponding Author: Mr Gabriele Russo

This file contains all editorial decision letters in order by version, followed by all author rebuttals in order by version.

Version 0:

Decision Letter:

** Please ensure you delete the link to your author home page in this e-mail if you wish to forward it to your coauthors **

Dear Mr Russo,

Your manuscript titled "Long-term ecological stability in the Pleistocene central Levantine corridor revealed by enamel isotopes from Lebanon" has now been seen by 2 reviewers, and we include their comments at the end of this message. They find your work of interest, but some important points are raised. We are interested in the possibility of publishing your study in Communications Earth & Environment, but would like to consider your responses to these concerns and assess a revised manuscript before we make a final decision on publication.

We noticed that the manuscript cites a study (your reference [20]) which is still under review, and we would like to inform you that the acceptance of your manuscript would depend on the acceptance of that cited one.

We therefore invite you to revise and resubmit your manuscript, along with a point-by-point response that takes into account the points raised. Please highlight all changes in the manuscript text file.

Please submit your point-by-point responses as a separate file, distinct from your cover letter where you can add responses to the Editors' comments that you do not want to be made available to the reviewers. Word files are preferred. We recommend that any figures, tables or graphs that are included in the response to reviewers are also included in the main article or Supplementary Information.

We are committed to providing a fair and constructive peer-review process. Please don't hesitate to contact us if you wish to discuss the revision in more detail.

Please use the following link to submit your revised manuscript, point-by-point response to the referees' comments (which should be in a separate document to any cover letter), a tracked-changes version of the manuscript (as a PDF file) and the completed checklist:
Link Redacted
** This url links to your confidential home page and associated information about manuscripts you may have submitted or be reviewing for us. If you wish to forward this email to co-authors, please delete the link to your homepage first **

We hope to receive your revised paper within six weeks; please let us know if you aren't able to submit it within this time so that we can discuss how best to proceed. If we don't hear from you, and the revision process takes significantly longer, we may close your file. In this event, we will still be happy to reconsider your paper at a later date, as long as nothing similar has been accepted for publication at Communications Earth & Environment or published elsewhere in the meantime.

Please do not hesitate to contact us if you have any questions or would like to discuss these revisions further. We look forward to seeing the revised manuscript and thank you for the opportunity to review your work.

Best regards,

Yama Dixit
Editorial Board Member
Communications Earth & Environment

Mengjie Wang
Associate Editor, Communications Earth & Environment
Consulting Editor, Communications Sustainability
Bluesky: @commsearth.nature.com; @commssustain.nature.com

EDITORIAL POLICIES AND FORMATTING

We ask that you ensure your manuscript complies with our editorial policies. Please ensure that the following formatting requirements are met, and any checklist relevant to your research is completed and uploaded as a Related Manuscript file type with the revised article.

For Manuscripts that fall into the following fields:
• Behavioural and social science
• Ecological, evolutionary & environmental sciences
• Life sciences
An updated and completed version of our Reporting Summary must be uploaded with the revised manuscript
You can download the form here:
https://www.nature.com/documents/nr-reporting-summary.pdf

Furthermore, please align your manuscript with our format requirements, which are summarized on the following checklist:
Communications Earth & Environment formatting checklist

and also in our style and formatting guide Communications Earth & Environment formatting guide .

*** DATA: Communications Earth & Environment endorses the principles of the Enabling FAIR data project (http://www.copdess.org/enabling-fair-data-project/). We ask authors to make the data that support their conclusions available in permanent, publically accessible data repositories. (Please contact the editor if you are unable to make your data available).

All Communications Earth & Environment manuscripts must include a section titled "Data Availability" at the end of the Methods section or main text (if no Methods). More information on this policy, is available at http://www.nature.com/authors/policies/data/data-availability-statements-data-citations.pdf.

In particular, the Data availability statement should include:
- Unique identifiers (such as DOIs and hyperlinks for datasets in public repositories)
- Accession codes where appropriate
- If applicable, a statement regarding data available with restrictions
- If a dataset has a Digital Object Identifier (DOI) as its unique identifier, we strongly encourage including this in the Reference list and citing the dataset in the Data Availability Statement.

DATA SOURCES: All new data associated with the paper should be placed in a persistent repository where they can be freely and enduringly accessed. We recommend submitting the data to discipline-specific, community-recognized repositories, where possible and a list of recommended repositories is provided at http://www.nature.com/sdata/policies/repositories.

If a community resource is unavailable, data can be submitted to generalist repositories such as figshare or Dryad Digital Repository. Please provide a unique identifier for the data (for example a DOI or a permanent URL) in the data availability statement, if possible. If the repository does not provide identifiers, we encourage authors to supply the search terms that will return the data. For data that have been obtained from publically available sources, please provide a URL and the specific data product name in the data availability statement. Data with a DOI should be further cited in the methods reference section.

Please refer to our data policies at http://www.nature.com/authors/policies/availability.html.

REVIEWER COMMENTS:

Reviewer #1 (Remarks to the Author):

This is an excellent paper that addresses a major lacuna in Levantine Paleolithic archaeology: the paleoclimatology and paleoecology of the Lebanese coast in the Middle to Late Pliocene. It used material from legacy faunal collections, which have remained understudied and thus provide a new wealth of evidence.

There are some minor issues in the paper that need to be fixed before acceptance.
A. Is there any data from modern species in Lebanon, such as wild goat or modern museum specimens of fallow deer, etc., that can be used as a baseline for comparison? If not, explain why not?
B. The authors use the terms Levant, Northern Levant, Central Levant, and Southern Levant as well as Eurasia without clear geographic borders. I would recommend a general amp to delineate these regions.
C. They compare MAT and MAP to modern day beirut. Is there a comparison for the degree of seasonal variation?
D. Line 70 - 73 "This configuration likely made this area a relatively stable refugium for humans and animals during globally cold and arid climatic phases over at least the past 250 ka, underscoring that the Levant was never a climatically homogeneous region. " How is this different from the Southern Levant, which is also not a climatically homogenous region, but each region is stable within itself? If we agree that the southern Levant was unstable and the Northern Levant was unstable, how can we explain the stability of the central Levant?
D. Line 268-269 "This long-term ecological stability underpinned repeated phases of hominin occupation and dispersal, which favored cultural innovation, and population turnover in the region." I disagree. A stable environment does not promote species turnover but rather depresses it, as does cultural invasion, which is often a response to risk and a volatile environment rather than a stable one. This has to be explained further and expanded upon. It may promote risk-prone dispersals as mentioned in lines 387 - 398, but that is only if you assume that dispersals are motivated by a 'pull' and by a need to occupy similar environments as opposed to a 'push' and a need to disperse from adverse conditions. If this is the main conclusion of the paper (lines 390 - 394), it needs to be discussed more thoroughly, as it is not obvious.
E. Line 307-308 "The stable conditions inferred for this period contrast sharply with the pronounced climatic oscillations documented at contemporaneous southern Levantine sites." I disagree. There is ample evidence for stability in MIS 6-2 (e.g., Belmaker and Hovers 2011, Belmaker 2017) within patterns of ossicaltions but more importantly, the different paleoclimatological and paleoecological proxies provide different scales of variability. So, a decrease in rainfall by 100 mm in Beirut does not have the same effect on the flora and biota, and subsequently on humans, as a decrease in rainfall by 100 mm in Beer Sheva. But stable isotopes pick up differences on a different scale than other methods, like pollen spectra. This is also relevant to the sentence in lines 373 - 375, which seems overly generalized and a throwaway.
F. Lines 377-379: Archaeological large mammal fauna may also be affected by human selectivity, large hunting ranges, and bias, and it may be difficult to argue that they are a fine-scale environmental proxy for the site where they were found.
G. Small issues:
1. Lines 22-24 "During the Middle and Late Pleistocene, the Levant served as a key land bridge between continents and witnessed repeated hominin dispersals, including the major expansion of modern humans out of Africa". The Levant was always a key land bridge, before and after the Middle to Late Pleistocene. Please revise.
2. Line 40 replace hominins with modern humans.
3. LInes 43-44 "The Levantine corridor, the only persistent land bridge between Africa and Asia over the past 500 ka (thousand years), positioned at the interface of Mediterranean, Saharan, and Eurasian regional climatic systems, constituted one such viable pathway" -- this is unclear. If the Levantine corridor is the only persistent land bridge how can it also be "one such" viable pathway. Moreover, there is clear evidence for dispersal between Africa and Asis across the rim of the Arabian peninsula and the Indian subcontinent during this time.
4. Line 46 "Its exceptionally rich archaeological record attests to its importance to hominins and gives it a pivotal role in human evolutionary studies. Continuously inhabited throughout the Pleistocene" is a redundant sentence. I would delete.
5. Line 51 "and also" should be "that".
6. Lines 58-59 "arid glacials and humid interglacials (e.g., 8–10)." The jury is still out on the arid vs. humid interglacial debate (See Bar Metthew papers and Frumkin papers). I would rephrase and include additional references.
7. Line 65 "across the northern Levantine regions becomes" should be either 'central Levantine regions' or the 'more northern Levant'.
Line 67 "paleoenvironmental data from the northern Levant (Lebanon, and western Syria) indicate that its coastal zone was more strongly influenced by Mediterranean climatic systems than by the African monsoon, in contrast to the southern Levant" The monsoon has a strong impact on the Negev and southern regions of the Southern Levant but not not on the coastal plain or in the centeral and northern regions of the Southern Levant. (Mauz et al 2012)
Line 73: change ',' to ';' after the word 'corridor'.
Line 91: change 'Human' to 'Homo' or 'Hominins'.
Line '140' 'values' should be 'value'
line 227: delete the repeated word "seasonal"
Line 349: remove the first 'values' in the sentence.

Reviewer #2 (Remarks to the Author):

The paper by Russo and colleagues uses a plethora of stable isotopic and other environmental proxy to shed light on the long-term environmental conditions in the northern Levantine woodland zone. The data provided give a rare insight into regional hydrology, aridity and seasonality in a time of great human mobility in the region. In my opinion, the manuscript will be highly interesting for the readership of Nat Comm Earth and Environment. Before publication however, I recommend some restructuring of the manuscript.

The manuscript rests on a variety of mainly stable isotopic approaches that differ slightly in their sampling, standards and

analytic methodologies. It would be good to highlight the different methods (carbonates, phosphates), sampling strategy (dentine, enamel, bulk, sequential etc.) the standards used, and what the specific analyses are informing us about, e.g., hydrology, vegetation structure, seasonality etc) – in the introduction - maybe even in tabular form. In addition, the manuscript lacks consistency in the notation of used analyses e.g., delta 13 C diet vs per mill (VBPD) and between the text, captions and figures (e.g., fig 2). Please decide on a clear and consistent way of referring to all different analyses (and add those to the table).

Equally, there are some inconsistencies in referring to the sites and/or distinct assemblages from sites e.g., for Naame. See e.g., line 412. This makes the narrative harder to follow. Especially for Naame with its huge chronological span, it is imperative to consistently mention the specific assemblage meant.

With regard on the substantial chronological time-spans, the use of old collections, the inherent risk of mixed assemblages, and the low sample sizes, I would like to see an in-depth discussion on the likely extent of time averaging on the sampled materials, and what effect that could have on the provided results. There are only a few datapoints for ~400.000 years. Can you really with confidence state that there is long-term ecological stability? Please substantiate and perhaps moderate relevant statements in the discussion.

Another issue that may influence the dataset is the potential for migratory activity, especially in given the huge differences in elevation in the landscapes adjacent to the sites. Please discuss in more detail the likelihood of this occurring (which is perhaps different for different taxa) and what the effect would be on the provided results.
To increase clarity, it would be good to exhaustively state which animals are taken to be ES and EI e.g., in the § about relative aridity variations, lines 234-242. Here a slight restructuring of the text may be helpful. Please consider dealing with the issue of (non-)obligatory drinkers in the introduction and clearly state the expectations for the results instead of repeated post-hoc explanations.

The introduction would benefit from some more substantial referencing of relevant literature e.g., Line 55-56. Line 62 but see Bosch et al. 2015, 2018. Line 77 idem and Bosch 2018.

Why is Ras el Kelb omitted from the analyses in figure 6?

Please consider to put Fig S6 in the main text.

Bosch, D. M. (2018). Molluscs in the Levantine Upper Palaeolithic: implications for modern human diets and subsistence behaviour [PhD]. Universiteit Leiden. https://hdl.handle.net/1887/61828

Bosch, M. D., Mannino, M. A., Prendergast, A. L., OConnell, T. C., Demarchi, B., Taylor, S. M., Niven, L., van der Plicht, J., & Hublin, J.-J. (2015). New chronology for Ksâr Akil (Lebanon) supports Levantine route of modern human dispersal into Europe. Proceedings of the National Academy of Sciences, 112(25), 7683-7688. 10.1073/pnas.1501529112

Bosch, M. D., Mannino, M. A., Prendergast, A. L., Wesselingh, F. P., O'Connell, T. C., & Hublin, J.-J. (2018). Year-round shellfish exploitation in the Levant and implications for Upper Palaeolithic hunter-gatherer subsistence. Journal of Archaeological Science: Reports, 21, 1198-1214. 10.1016/j.jasrep.2017.08.014

** Visit Nature Portfolio's author and referees' website at www.nature.com/authors for information about policies, services and author benefits**

Communications Earth & Environment is committed to improving transparency in authorship. As part of our efforts in this direction, we are now requesting that all authors identified as 'corresponding author' create and link their Open Researcher and Contributor Identifier (ORCID) with their account on the Manuscript Tracking System prior to acceptance. ORCID helps the scientific community achieve unambiguous attribution of all scholarly contributions. You can create and link your ORCID from the home page of the Manuscript Tracking System by clicking on 'Modify my Springer Nature account' and following the instructions in the link below. Please also inform all co-authors that they can add their ORCIDs to their accounts and that they must do so prior to acceptance.
https://www.springernature.com/gp/researchers/orcid/orcid-for-nature-research

For more information please visit http://www.springernature.com/orcid

If you experience problems in linking your ORCID, please contact the Platform Support Helpdesk.

Version 1:

Decision Letter:

** Please ensure you delete the link to your author home page in this e-mail if you wish to forward it to your coauthors **

Dear Mr Russo,

Your manuscript titled "Long-term ecological stability in the Pleistocene central Levantine corridor revealed by enamel isotopes from Lebanon" has now been seen by our editors. We are delighted to say that we are happy, in principle, to publish the revised version in Communications Earth & Environment on the condition that the cited paper once mentioned is accepted as well.

At the same time we ask that you edit your manuscript to comply with our format requirements and to maximise the accessibility and therefore the impact of your work.

EDITORIAL REQUESTS:

Please review our specific editorial comments and requests regarding your manuscript in the attached "Editorial Requests Table".

*****Please take care to match our formatting and policy requirements. We will check revised manuscript and return manuscripts that do not comply. Such requests will lead to delays. *****

Please outline your response to each request in the right hand column. Please upload the completed table with your manuscript files as a Related Manuscript file.

If you have any questions or concerns about any of our requests, please do not hesitate to contact me.

SUBMISSION INFORMATION:

In order to accept your paper, we require the files listed at the end of the Editorial Requests Table; the list of required files is also available at https://www.nature.com/documents/commsj-file-checklist.pdf .

OPEN ACCESS:

Communications Earth & Environment is a fully open access journal. Articles are made freely accessible on publication. For further information about article processing charges, open access funding, and advice and support from Nature Portfolio, please visit https://www.nature.com/commsenv/open-access

At acceptance, you will be provided with instructions for completing the open access licence agreement on behalf of all authors. This grants us the necessary permissions to publish your paper. Additionally, you will be asked to declare that all required third party permissions have been obtained, and to provide billing information in order to pay the article-processing charge (APC).

Please use the following link to submit the above items:
Link Redacted
** This url links to your confidential home page and associated information about manuscripts you may have submitted or be reviewing for us. If you wish to forward this email to co-authors, please delete the link to your homepage first **

We hope to hear from you within two weeks; please let us know if you need more time.

Best regards,

Yama Dixit
Editorial Board Member
Communications Earth & Environment

Mengjie Wang
Associate Editor, Communications Earth & Environment
Consulting Editor, Communications Sustainability
Bluesky: @commsearth.nature.com; @commssustain.nature.com

** Visit Nature Portfolio's author and referees' website at www.nature.com/authors for information about policies, services and author benefits**

Open Access This Peer Review File is licensed under a Creative Commons Attribution 4.0 International License, which permits use, sharing, adaptation, distribution and reproduction in any medium or format, as long as you give appropriate credit to the original author(s) and the source, provide a link to the Creative Commons license, and indicate if changes were made.
In cases where reviewers are anonymous, credit should be given to 'Anonymous Referee' and the source.
The images or other third party material in this Peer Review File are included in the article's Creative Commons license, unless indicated otherwise in a credit line to the material. If material is not included in the article's Creative Commons license and your intended use is not permitted by statutory regulation or exceeds the permitted use, you will need to obtain permission directly from the copyright holder.
To view a copy of this license, visit https://creativecommons.org/licenses/by/4.0/

Response to the reviewers

To Reviewer 1

We thank the reviewer for their positive evaluation of our manuscript and for recognizing the contribution of this study to Levantine Paleolithic research. We appreciate the comments and suggestions, which have helped us improve the clarity and quality of the manuscript. Below, we address each point in detail.

Reviewer 1 comment:

A. *Is there any data from modern species in Lebanon, such as wild goat or modern museum specimens of fallow deer, etc., that can be used as a baseline for comparison? If not, explain why not?*

Response:

Thank you for this thoughtful suggestion. We agree that modern faunal enamel isotope data from Lebanon would provide a valuable absolute baseline for comparison. However, to our knowledge, no published stable isotope dataset currently exists for modern wild Lebanese herbivores.

Establishing such a baseline would require a dedicated project with locating appropriate modern specimens (e.g., museum collections or contemporary field collections), acquiring relevant permissions for sampling them, and then sampling and analyzing them. These activities would form a separate study which would fall beyond the scope of the present manuscript and the timeframe for revising it.

Even though having this data would provide interesting comparisons, we would like to note that its absence is not a drawback as here we have taken the approach to interpret enamel $\delta^{13}C$ and $\delta^{18}O$ values following comparisons among taxa and assemblages and using established fractionation frameworks. Additionally, we have used modern precipitation and water isotope datasets for the eastern Mediterranean as contextual baselines for $\delta^{18}O$ variability and elevation effects.

We hope that this clarification addresses the reviewer's concern.

Reviewer 1 comment:

B. *The authors use the terms Levant, Northern Levant, Central Levant, and Southern Levant as well as Eurasia without clear geographic borders. I would recommend a general amp to delineate these regions.*

Response:

We thank the reviewer for this comment. We would like to clarify that our use of general geographic terms follows established conventions in the paleoanthropological and paleoenvironmental literature (e.g. Enzel & Bar-Yosef, 2017), where these designations are typically employed as broad biogeographic, paleoenvironmental, and climatic descriptors rather than as strictly bounded geographic units. Because our discussion concerns Pleistocene environmental gradients and ecological partitioning, and because no detailed paleoenvironmental studies that delineate these gradients and partitions are available for the Levantine region, we intentionally avoid imposing fixed geographic boundaries that might not necessarily reflect past landscape configurations. We therefore prefer to maintain the current usage, which is consistent with the broader literature and more appropriate for the scale of analysis presented here.

Reviewer 1 comment:

C. *They compare MAT and MAP to modern day beirut. Is there a comparison for the degree of seasonal variation?*

Response:

We agree that explicitly quantifying modern seasonality strengthens the interpretation of reconstructed intra-annual $\delta^{18}O$ amplitudes. We would like to draw the reviewer's attention to Supplementary Figure S5 which was already included in the original submission. This figure presents modern seasonal temperature and precipitation patterns for coastal Lebanon with data extracted from the WorldClim 2.1 database (1980–2000). It illustrates mean monthly temperature and precipitation values in coastal Lebanon extent (35-36°E; 33-34.7°N), showing the characteristic Mediterranean climatic cycle of warm, dry summers and cool, wet winters.

This figure was referenced in the main text's discussion section as follows:

"Herbivore $\delta^{18}O$ profiles in all three collections mostly display regular sinusoidal cycles that closely track modern Mediterranean seasonality, characterized by cool-wet winters and warm-dry summers (Figure S5)."

To further address the reviewer's comment and provide a clearer quantitative descriptor of seasonality, we have now added the following phrase to the sentence above:

"with ~70% of annual rainfall falling between November and March,"

This phrase explicitly quantifies the strong seasonal precipitation concentration during the wet/winter season.

Reviewer 1 comment:

D. *Line 70 - 73 "This configuration likely made this area a relatively stable refugium for humans and animals during globally cold and arid climatic phases over at least the past 250 ka, underscoring that the Levant was never a climatically homogeneous region. " How is this different from the Southern Levant, which is also not a climatically homogenous region, but each region is stable within itself? If we agree that the southern Levant was unstable and the Northern Levant was unstable, how can we explain the stability of the central Levant?*

Response:

We realize that our use of the term "stable refugium" was inappropriate for the argument we intended to make. We have revised the text to clarify that we discuss that the steep topographic gradients and orographic precipitation would have led to relative buffering of local ecohydrology in the Lebanese coastal-mountain ecotone. This buffering could have maintained mesic habitats locally even with a trend for broader regional drying. We also add supporting citations on Levantine hydroclimate gradients and on proxy evidence for spatially heterogeneous moisture level changes across the eastern Mediterranean (e.g., Kushnir et al. 2017) to support our argument (Lines 69-76).

Reviewer 1 comment:

E. *Line 268-269 "This long-term ecological stability underpinned repeated phases of hominin occupation and dispersal, which favored cultural innovation, and population turnover in the region." I disagree. A stable environment does not promote species*

turnover but rather depresses it, as does cultural invasion, which is often a response to risk and a volatile environment rather than a stable one. This has to be explained further and expanded upon. It may promote risk-prone dispersals as mentioned in lines 387 - 398, but that is only if you assume that dispersals are motivated by a 'pull' and by a need to occupy similar environments as opposed to a 'push' and a need to disperse from adverse conditions. If this is the main conclusion of the paper (lines 390 - 394), it needs to be discussed more thoroughly, as it is not obvious.

Response:

We appreciate this comment. Similar to what we stated above, we realized that we had not correctly articulated our intended argument in the previous version of the manuscript. We have now revised the part of the Discussion relevant to this comment. We have removed reference to the term "stability" but instead discuss ecological buffering (a property of local habitats) that our results actually imply. The latter would have allowed the central Levantine corridor to remain habitable during a range of climatic phases, potentially affecting hominin occupations and movements across the landscape.

Reviewer 1 comment:

F. *Line 307-308 "The stable conditions inferred for this period contrast sharply with the pronounced climatic oscillations documented at contemporaneous southern Levantine sites." I disagree. There is ample evidence for stability in MIS 6-2 (e.g., Belmaker and Hovers 2011, Belmaker 2017) within patterns of ossicaltions but more importantly, the different paleoclimatological and paleoecological proxies provide different scales of variability. So, a decrease in rainfall by 100 mm in Beirut does not have the same effect on the flora and biota, and subsequently on humans, as a decrease in rainfall by 100 mm in Beer Sheva. But stable isotopes pick up differences on a different scale than other methods, like pollen spectra. This is also relevant to the sentence in lines 373 - 375, which seems overly generalized and a throwaway.*

Response:

We appreciate this note. We agree that different paleoenvironmental proxies capture variability at different spatial and temporal scales. Our intention was not to imply uniform instability across all southern Levantine sites, but rather to highlight that several isotopes, faunal, and palaeoecological records from specific sites and specific time periods document greater hydroclimatic amplitude or interannual variability relative to the more nuanced signal recorded in the Lebanese coastal assemblages.

To address this note, we have now revised the text to avoid overly generalized contrasts, acknowledge the mosaic nature of southern Levantine environments, and clarify that the comparison concerns relative hydroclimatic amplitude and proxy scale rather than stability versus instability in absolute terms (Lines 414-418).

Reviewer 1 comment:

G. *Lines 377-379: Archaeological large mammal fauna may also be affected by human selectivity, large hunting ranges, and bias, and it may be difficult to argue that they are a fine-scale environmental proxy for the site where they were found.*

Response:

We agree that archaeological large mammal assemblages may be influenced by several factors and might not form a fine-scale environmental proxy for the site where they were found. We have thus revised the relevant section of the text to clarify that the isotopic signal reflects environments used by the sampled animals rather than a narrowly defined site-specific microhabitat.

With respect to the isotopic analyses presented here, enamel $\delta^{13}C$ and $\delta^{18}O$ values reflect the environmental conditions experienced by the sampled animals during tooth formation, which may span different years and localities within their foraging ranges. Even if this does not equate to a strictly site-specific proxy, it still provides fine-scale (intra-annual) environmental information that is averaged across individuals and time, allowing broader landscape-level patterns to emerge.

We would also like to add that the structure and diversity patterns of these same legacy collections studied here have been examined in detail in a related manuscript Russo et al. (in review, Communications Earth & Environment). There it has been demonstrated that abundance-weighted diversity patterns remain stable when standardized by coverage, supporting the reliability of long-term ecological inferences at a landscape scale despite inherent biases.

<u>Small issues:</u>

Reviewer 1 comment:

1. *Lines 22-24 "During the Middle and Late Pleistocene, the Levant served as a key land bridge between continents and witnessed repeated hominin dispersals, including the major expansion of modern humans out of Africa". The Levant was always a key land bridge, before and after the Middle to Late Pleistocene. Please revise.*

Response:

We have rephrased the sentence to emphasize the Levant's long-standing geographic role as a land corridor throughout the Pleistocene.

2. *Line 40 replace hominins with modern humans.*

Response:

We have revised the wording to "multiple hominin species," as this sentence refers to repeated dispersal events involving different hominin taxa throughout the Pleistocene rather than exclusively to modern humans.

3. *LInes 43-44 "The Levantine corridor, the only persistent land bridge between Africa and Asia over the past 500 ka (thousand years), positioned at the interface of Mediterranean, Saharan, and Eurasian regional climatic systems, constituted one such viable pathway" -- this is unclear. If the Levantine corridor is the only persistent land bridge how can it also be "one such" viable pathway. Moreover, there is clear evidence for dispersal between Africa and Asis across the rim of the Arabian peninsula and the Indian subcontinent during this time.*

Response:

We thank the reviewer for pointing out this possible source of confusion in our phrasing. The phrase "one such viable corridor" referred to the previous sentence where we talked about

“ecologically viable corridors” and did not refer to “land bridges”. We reworded slightly for clarity.

4. *Line 46 "Its exceptionally rich archaeological record attests to its importance to hominins and gives it a pivotal role in human evolutionary studies. Continuously inhabited throughout the Pleistocene" is a redundant sentence. I would delete.*

Response:

We have revised this passage to remove redundancy and improve clarity.

5. *Line 51 "and also" should be "that".*

Response:

We have corrected this as suggested.

6. *Lines 58-59 "arid glacials and humid interglacials (e.g., 8–10)." The jury is still out on the arid vs. humid interglacial debate (See Bar Metthew papers and Frumkin papers). I would rephrase and include additional references.*

Response:

We thank the reviewer for highlighting this important nuance. We agree that the characterization of Levantine glacials as uniformly arid and interglacials as uniformly humid oversimplifies a complex and ongoing debate. Multiple proxies, including speleothems, lake levels, pollen, and marine records, indicate that interglacial conditions were spatially and temporally variable, with some records suggesting enhanced moisture availability and others indicating relatively arid conditions (e.g., Kushnir et al. 2024; Frumkin et al. 2019; Weissbrod and Weinstein-Evron 2022; Frumkin and Comay 2022).

We have revised the sentence to avoid a binary framing and to better reflect the complexity of Levantine paleoclimate reconstructions.

7. *Line 65 "across the northern Levantine regions becomes" should be either 'central Levantine regions' or the 'more northern Levant'.*

Response:

We have revised the phrasing to “more northern Levantine regions,” as recommended.

Line 67 "paleoenvironmental data from the northern Levant (Lebanon, and western Syria) indicate that its coastal zone was more strongly influenced by Mediterranean climatic systems than by the African monsoon, in contrast to the southern Levant" The monsoon has a strong impact on the Negev and southern regions of the Southern Levant but not not on the coastal plain or in the centeral and northern regions of the Southern Levant. (Mauz et al 2012)

Response:

We thank the reviewer for this clarification. We have revised the sentence to avoid overgeneralizing the southern Levant and to acknowledge that monsoonal influence was spatially variable within the region.

Line 73: change ',' to ';' after the word 'corridor'.

Response:

The punctuation has been corrected.

Line 91: change 'Human' to 'Homo' or 'Hominins'.

Response:

“Early humans” has been replaced with “hominin.”

Line '140' 'values' should be 'value'

Response:

Corrected in the revised manuscript.

Response:

line 227: delete the repeated word "seasonal"

Response:

The duplicated word has been removed.

Line 349: remove the first 'values' in the sentence.

Response:

The sentence has been revised accordingly.

To Reviewer 2:

We are grateful to Reviewer 2 for their thoughtful and detailed comments. Their suggestions have greatly strengthened the organization, clarity, and interpretative framing of the manuscript. Below there is our point-by-point responses.

Reviewer 2 comment:

1. *It would be good to highlight the different methods (carbonates, phosphates), sampling strategy (dentine, enamel, bulk, sequential etc.) the standards used, and what the specific analyses are informing us about, e.g., hydrology, vegetation structure, seasonality etc) – in the introduction - maybe even in tabular form*.

Response:

We thank the reviewer for this helpful suggestion. Accordingly, we have added a concise summary in the Introduction outlining what the different analyses inform about (Lines 94-100).

Reviewer 2 comment:

2. *In addition, the manuscript lacks consistency in the notation of used analyses e.g., delta 13 C diet vs per mill (VBPD) and between the text, captions and figures (e.g., fig 2). Please decide on a clear and consistent way of referring to all different analyses (and add those to the table).*

Response:

We have carefully revised the manuscript to ensure consistent reporting of $\delta^{13}C$ and $\delta^{18}O$ values, clearly distinguishing between carbonate and phosphate measurements and explicitly stating the reference standards (VPDB or VSMOW) throughout the text, figures, and supplementary materials.

Reviewer 2 comment:

3. *Equally, there are some inconsistencies in referring to the sites and/or distinct assemblages from sites e.g., for Naame. See e.g., line 412. This makes the narrative harder to follow. Especially for Naame with its huge chronological span, it is imperative to consistently mention the specific assemblage meant.*

Response:

Thank you for highlighting this issue. We have carefully revised the manuscript to ensure consistent and explicit reference to the specific stratigraphic levels at Naame throughout the text. Where interpretations concern individual layers ($PIII_0$, $PIII_2$, *Niveau Supérieur*), these are now clearly specified. When discussing the assemblage as a whole, this is explicitly stated. We also now consistently distinguish between the MIS 5 deposits and the directly dated MIS 2 specimen to avoid chronological ambiguity.

Reviewer 2 comment:

4. *With regard on the substantial chronological time-spans, the use of old collections, the inherent risk of mixed assemblages, and the low sample sizes, I would like to see an in-depth discussion on the likely extent of time averaging on the sampled materials, and what effect that could have on the provided results. There are only a few datapoints for ~400.000 years. Can you really with confidence state that there is*

long-term ecological stability? Please substantiate and perhaps moderate relevant statements in the discussion.

Response:

We acknowledge that legacy collections spanning substantial chronological intervals are inherently subject to time-averaging and potential mixing. The extent of recovery, curation, and sampling bias in these assemblages is examined in detail in Russo et al. (under review, Communications Earth & Environment), where coverage-based standardization and diversity analyses demonstrate that, in the assemblages studied here, the overall taxonomic structure and abundance-weighted diversity patterns are robust despite inherited biases.

With respect to the isotopic analyses presented here, several lines of within sample results' consistency support the reliability of the environmental signal. The directly dated MIS 2 specimen from Naame yields isotopic values that are clearly distinct from the MIS 5 assemblage at the same site, indicating that chronological differences are detectable within the dataset. Likewise, assemblages from Naame and Nahr Ibrahim both, as expected, show coherent interglacial signals, whereas Ras el-Kelb displays systematically dampened values, consistent with site-specific environmental conditions rather than random mixing.

While time-averaging may smooth short-term variability, it is unlikely to generate on its own the coherent and repeated site-specific isotopic patterns observed here. We have now clarified this point at the beginning of the Discussion.

Reviewer 2 comment:

5. *Another issue that may influence the dataset is the potential for migratory activity, especially in given the huge differences in elevation in the landscapes adjacent to the sites. Please discuss in more detail the likelihood of this occurring (which is perhaps different for different taxa) and what the effect would be on the provided results.*

Response:

We thank the reviewer for this important point. In sequential enamel $\delta^{18}O$ profiles, seasonal or altitudinal migration is expected to dampen intra-tooth isotopic amplitudes by reducing exposure to seasonal environmental extremes (e.g., Pilaar Birch et al., 2016; Pederzani & Britton, 2019). In our dataset, most taxa from Naame and Nahr Ibrahim exhibit intra-tooth $\delta^{18}O$ amplitudes between ~2 to 5‰, which fall within the range documented for non-migratory ungulates. Only Ras el-Kelb shows lower amplitudes (~1 to 2‰); however, this reduction is consistent across taxa and therefore more parsimonious with reflections of a locally attenuated seasonality rather than taxon-specific mobility patterns.

Besides potential migratory behavior, potential elevation-dependent effects were explicitly considered in our interpretive framework. As discussed in both the main text and Supplementary Information, we account for altitudinal variation in precipitation $\delta^{18}O$ and compare taxa to evaluate differences in habitat use and water acquisition strategies.

The Discussion has been revised to clarify these points.

Reviewer 2 comment:

6. *To increase clarity, it would be good to exhaustively state which animals are taken to be ES and EI e.g., in the § about relative aridity variations, lines 234-242. Here a slight restructuring of the text may be helpful. Please consider dealing with the issue of (non-)obligatory drinkers in the introduction and clearly state the expectations for the results instead of repeated post-hoc explanations*

Response:

We agree that clearer upfront definition of evaporation-sensitive (ES) and evaporation-insensitive (EI) taxa improves readability. We have revised the relevant section to explicitly define which taxa are considered ES and EI in our study, and to clarify the expected isotopic pattern under relatively humid versus arid conditions prior to presenting the results.

Reviewer 2 comment:

7. *The introduction would benefit from some more substantial referencing of relevant literature e.g., Line 55-56. Line 62 □ but see Bosch et al. 2015, 2018. Line 77 idem and Bosch 2018.*

Response:

We have expanded the Introduction and Discussion to incorporate additional relevant literature, including Bosch et al. (2015, 2018), at the points as suggested.

Reviewer 2 comment:

8. *Why is Ras el Kelb omitted from the analyses in figure 6?*

Response:

Ras el-Kelb was excluded from the EI-ES aridity index analysis because the assemblage does not contain sufficient paired EI and ES taxa to allow a robust comparison. Similarly, the Naame - *Niveau Supérieur* specimens were excluded due to associated chronological uncertainty relative to the MIS 5 assemblage. We have now clarified these exclusions in the main text.
