# Supplementary material for: Long-term ecological stability in the Pleistocene central Levantine corridor revealed by enamel isotopes from Lebanon
Source: Commun Earth Environ. 2026 Sep 14;7(1):705. doi: 10.1038/s43247-026-03925-x (PMC13574713; doi:10.1038/s43247-026-03925-x)
Supplement: Supplementary file 3 [file 43247_2026_3925_MOESM1_ESM.docx]

Supplementary Materials for

**Long-term ecological stability in the Pleistocene central Levantine corridor revealed by enamel isotopes from Lebanon**

Gabriele Russo *et al.*

*Corresponding author. Email: [gabriele.russo@uni-tuebingen.de](mailto:gabriele.russo@uni-tuebingen.de)

**This PDF includes:**

[**Supplementary Notes**	2](#_Toc216512563)

[**S1. Archaeological Background of the studied samples**	2](#_Toc216512564)

[*Naame*	2](#_Toc216512565)

[*Nahr Ibrahim*	2](#_Toc216512566)

[*Ras el-Kelb*	3](#_Toc216512567)

[**S2. Stable isotopes extended methodology**	4](#_Toc216512568)

[S2.1 Stable isotopes of enamel carbonates as palaeoecological proxies	4](#_Toc216512569)

[S2.2 *δ*^13^C as proxy for diet and precipitation	5](#_Toc216512570)

[S2.3 Inverse modelling applied to intratooth profiles	6](#_Toc216512571)

[S.2.4 *δ*^18^O as a proxy for seasonal temperature estimation	7](#_Toc216512572)

[S2.5 Aridity Index	8](#_Toc216512573)

[S2.6. Cross-correlation analysis	9](#_Toc216512574)

[**S3. Stable isotopes extended results**	9](#_Toc216512575)

[S3.1. Preservation screening and data-quality assessment	9](#_Toc216512576)

[S3.2. Sequential isotope profiles	10](#_Toc216512577)

[S3.3. Mean *δ*^13^C results	10](#_Toc216512578)

[S3.4. Mean *δ*^18^O results and drinking-water reconstruction	11](#_Toc216512579)

[S3.5. Relative aridity index	12](#_Toc216512580)

[**S4. Supplementary Figures**	13](#_Toc216512581)

[**S5. Supplementary Tables**	19](#_Toc216512582)

[**S6. Supplementary References**	32](#_Toc216512583)

# **Supplementary Notes**

## **S1. Archaeological Background of the studied samples**

### *Naame*

The Naame archaeological site, was located about 15 km south of Beirut along the coast and approximately 12 m above sea level, discovered by H. Fleisch and P. Sanlaville in late 1966 ^1^. Excavations focused on three areas in front of exposed limestone blocks (PI, PII, PIII), with PIII identified as the most archaeologically rich ^1^; the faunal remains analyzed in this study come from this area.

Within PIII, three soundings of the exposed bone breccia profiles were made (referred to by superscripts, i.e., P^1^III, P^2^III, P^3^III). Based on lithic density, three stratigraphic sections were identified within each of the soundings (from top to bottom further labeled by subscripts, e.g., P^1^III_3_-P^1^III_0_. These sections were more or less uniform across the three profiles with sections III_2_ and III_0_ containing the highest concentration of artifacts. The lowest section III_0_, rested directly atop a shell-rich conglomerate composed of *Strombus bubonius* and vermetids, indicative of Pleistocene sea oscillations. U-series dating of these shells yielded ages of 90 ± 10 ka and 90 ± 20 ka ^2^. However, they should probably be regarded as purely indicative rather than proper chronological constraints. Indeed, the reliability of these age results cannot be properly assessed in the absence of any information regarding the analytical procedure employed, or the data obtained.

At the eastern edge of P^1^III, Fleisch also excavated softer sediments above the hard breccia deposit, defining a *Niveau Superieur* (upper level) divided into ground soil and decalcified breccia. In 1975, prior to the site’s destruction for highway construction, additional trenches confirmed the previous stratigraphic observations ^3^. All lithic material from Naame has been attributed to the Middle Paleolithic ^1,3^.

Recently, new chronological U-series/ESR analyses was conducted on three faunal dental remains from Naame ^4^. Two teeth, one recovered from the *Niveau Supérieur* (sample #860) returned a combined U-series/ESR age of 107 ± 13 ka (1*σ*) providing a reliable finite estimate, and one (sample #859) from the lowermost stratigraphic unit (PIII_0_), provided an age range within MIS 5 (130-71 ka). These dates validated the previous age inferences available for the site. In contrast, a third tooth (sample #862), also from the *Niveau Supérieur*, was found to be much younger, i.e., dated to MIS 2 (29-14 ka), with U-series indicating a minimum age of 6.9 ka and a combined U-series/ESR age of 17.6 ± 2.7 ka (1*σ*). Therefore, these data suggest that the *Niveau Supérieur* may contained a mixture of Middle Paleolithic and much younger material, although the proportion of the latter is currently unknown. It should be noted that these same dated specimens were also analyzed in this study for their stable isotope composition (sample IDs: #859 = NME2, #860 = NME20, #862 = NME51).

### *Nahr Ibrahim*

Nahr Ibrahim Cave (also known as *Mogharat al Asfourieh*) is located approximately 30 km north of Beirut, roughly 70 m from the current shoreline, at an elevation of about 15 m above sea level. The cave takes its name from the nearby Ibrahim River, situated around 800 m south of the karst formation. Known since the 19th century, it was first studied by the Jesuit father G. Zumoffen, towards the end of that century ^5^. The cave’s interior, comprising a total area of ca. 200 m^2^, is divided into three main galleries (north, central, and main gallery) and one smaller one (south gallery) ^6^. It should be noted that the cave has been repeatedly modified through time for the use of its space, with sediment removal and alterations occurring from antiquity up until recent times, resulting in varying degrees of disturbance to the original deposits.

In 1890, Zumoffen recovered a collection of lithics and osseous remains from under a collapsed roof accumulation in the central gallery, which he identified as preserving the least disturbed Pleistocene layers ^5,7^. The faunal material analyzed in this current study originates from that collection. Subsequent excavations at Nahr Ibrahim Cave, carried out between 1969 and 1973 by R. Solecki ^6,8^, provided new insights into the site’s formation processes and stratigraphic sequence. These investigations, of the stratigraphy and also the lithics, attributed the deposits to the Middle Paleolithic.

Later studies of the lithic assemblages recovered from Solecki’s excavations attempted to refine the position of the Nahr Ibrahim material and deposits. These suggest that the earliest deposits are found in the north gallery and possibly date back to late MIS 7/early MIS 6, whereas the central gallery presented younger material, with that from layer 4 consistent with MIS 5 typologies, and that from layer 3 typologically consistent with post-MIS 5 ^9,10^. These estimates would fit well with a preliminary U-series age of ~100 ka from a capping flowstone, which could potentially provide a minimum age for the underlying deposits ^11^. However, this age should be treated with caution given the absence of any minimum information regarding the methodology employed and the dating results, while the exact location of the sample has not been reported. More recently, Russo et al. ^4^ obtained new U-series/ESR dating results on a tooth from Zumoffen’s collection. Sensitivity tests, accounting for fluctuations in sedimentary heterogeneity and water content, consistently yielded ages of 200–300 ka for this tooth (with a minimum U-series age of 108.5 ka and a finite US-ESR age of 245 ± 23 ka - 1*σ*), securely placing these specimens in the Middle Pleistocene. These dates broadly coincide with the typology of the oldest lithic material from the site. We also conducted stable isotope analysis on this dated tooth (sample ID: #863 = NIM2).

### *Ras el-Kelb*

The Ras el-Kelb promontory is a limestone formation descending from the east into the Mediterranean Sea. Located approximately 15 km north of Beirut atop the delta of the el-Kalb River, from which it takes its name. The site was a crucial crossroads in antiquity. The steep eastern landscape made it a natural transit point along the coast, where numerous military expeditions left carvings and inscriptions that remain visible today.

Bone breccia deposits at Ras el-Kelb were first noted by Tristram in 1863 ^12^. But, when, in 1874, railroad construction cut through the lower side of the promontory, it exposed further deposits prompting Zumoffen to conduct the first archaeological investigations there. During these early explorations, Zumoffen documented several localities containing Paleolithic lithic artifacts and Pleistocene faunal remains ^5^. The faunal assemblage analyzed in the present study originates from one of these localities, situated a few meters above the better-known Ras el-Kelb Cave, which was excavated several decades later.

The entrance to Ras el-Kelb Cave itself was exposed and destroyed in 1942, during the construction of the Haifa–Tripoli railway, but which exposed further brecciated cave deposits. The exposure of these additional deposits prompted further investigations by the Jesuit father H. Fleisch, who recovered additional faunal material, including rhinoceros remains ^13^. By 1959, the promontory was perforated for constructing two road tunnels, with the westernmost cutting through the rear of the cave. A subsequent rescue excavation, directed by D. Garrod and D. Henri-Martin, included a small test trench along the former railway and further trench excavations within the tunnel interior ^14^.

The Ras el-Kelb Cave deposits have traditionally been attributed to MIS 5 ^10^; however, this estimate is based on comparisons of techno-typological lithic assemblages and of geomorphological features of ancient shorelines rather than on numerical dating results ^2,15,16^. Importantly, the early explorations of the promontory by Zumoffen and Fleisch also recovered lithic material attributed to the Acheulian in the area surrounding the cave, suggesting the presence of Lower Paleolithic human occupations. More recently, Russo et al. ^4^ analyzed a tooth from Zumoffen’s collection of Ras el-Kelb (sample #861), obtaining a minimum U-series age of 135.8 ka for the dentine and a combined U-series/ESR age of 402 ± 46 ka (1*σ*) for the enamel, providing a reliable finite estimate and firmly situating the specimen within the Middle Pleistocene. The dated tooth was included in the present study for stable isotope analysis (sample ID: #861 = REK46). Finally, this new numerical age results should not be regarded as a constraint for the assemblage from Ras el-Kelb Cave, as its stratigraphic correlation with the Zumoffen’s collection remains currently unknown and requires further investigation.

## **S2.** **Stable isotopes extended methodology**

### S2.1 Stable isotopes of enamel carbonates as palaeoecological proxies

The stable carbon isotope composition (*δ*^13^C) of terrestrial herbivore tooth enamel provides valuable insights into past dietary sources as well as environmental conditions. In regions dominated by C3 plants, such as Mediterranean ecosystems, *δ*^13^C values primarily reflect variations in climate, with precipitation playing a more significant role than temperature in shaping isotopic signatures ^17–19^. Additionally, they also reflect canopy cover as enclosed forest environments tend to produce lower *δ*^13^C values due to limited light penetration, increased humidity, and the recycling of plant-derived CO_2_ ^18,20^. In contrast, open and arid landscapes result in elevated *δ*^13^C values in plants and subsequently in the tissues of herbivores consuming them ^21^.

Since plant *δ*^13^C is influenced by rainfall ^22–24^, herbivore enamel *δ*^13^C can be used a proxy for the estimation of mean annual precipitation (MAP) (See S2.3). However, it is important to note that these estimates remain broad approximations, as enamel values are also affected by dietary preferences and ecological factors beyond precipitation alone ^25^.

Oxygen isotope values (*δ*^18^O) in enamel carbonate and phosphate fractions also reflect climatic factors as they are influenced by the sources of ingested water which in turn are affected by factors such as temperature and aridity ^26–28^. In homeothermic terrestrial mammals, the local hydrological cycle result in variations in *δ*^18^O values of body water. At middle and high latitudes, seasonal shifts in temperature and moisture lead to variations in their *δ*^18^O of drinking water ^29,30^. These variations are exhibited as sinusoidal seasonal patterns in sequential intra-tooth enamel *δ*^18^O values of mammals, with peaks corresponding to warmer summer conditions and troughs reflecting colder winter temperatures.

However, the extent to which an animal’s *δ*^18^O signature reflects climatic fluctuations in the source of its dietary water depends on the species’ drinking behavior, with that of obligate drinkers closely mirroring local precipitation patterns ^31^, which in turn tracks climatic temperature, whereas that of non-obligate drinkers generally exhibit greater variability resulting from differences in water intake in the plants they consume. Therefore, the combination of the strong correlation between *δ*^18^O in meteoric waters (*δ*^18^O_mw_) and mean air temperature, and the transfer of *δ*^18^O from drinking water into the bioapatite of obligate-drinking mammals, such as horses and bovids, allows for the latter to be used for the reconstruction of past air temperatures ^32–35^ (see S2.4). Underlying assumptions of this method are that animals consumed mainly precipitation-fed waters and that modern *δ*^18^O-temperature relationships are broadly applicable to Pleistocene conditions, if supported by circulation models and groundwater records ^32,36,37^. Despite inherited uncertainties due to error propagations, enamel *δ*^18^Oof obligate drinker taxa still provides a reliable proxy for estimating mean annual and seasonal palaeotemperatures ^35^.

To establish a robust seasonal framework for isotopic analysis, mineralization sequences for the examined taxa were compiled from previous studies that used radiographic imaging and eruption sequence analyses. Among cervids, red deer exhibits M3 mineralization between 13 and 18 months, while M2 forms between 3 and 9 months ^38^. The closely related European fallow deer (*Dama dama*) follows a similar pattern, with P4 forming between 8 and 16 months, M2 between 3.5 and 10 months, and M3 between 10 and 18 months ^39,40^. Equids, such as wild horse (*Equus ferus caballus*), display a more prolonged mineralization period, with M3 developing between the second and fourth years of life, capturing up to three summers and two winters within its enamel ^29^. In bovids, the domestic cattle (*Bos taurus*) P4 initiating development starts at 11 months and is completed by 24 months. The M1 forms in utero and is complete within 2–3 months, while M2 spans 1-13 months, and M3 between 9 and 24 months ^41,42^. The closely related bison (*Bison bison*) follows a comparable structured formation ^43^. Caprines, such as domestic goat (*Capra hircus)* and sheep (*Ovis aries*), exhibit variable but generally consistent patterns, with M2 forming between 1 and 12 months and M3 between 9 and 24 months ^44–46^. Among rhinocerotids, mineralization is a more prolonged process, with M1 and M2 initiating in utero and requiring up to two years for completion, while M3 forms postnatally over three years ^47,48^. The P3 mineralizes between 9 and 24 months, after erupting at approximately two years of age ^49^. In mammals, early-mineralizing teeth, such as M1 and portions of M2, may retain isotopic signatures influenced by nursing, as milk exhibits higher *δ*^18^O values than environmental water and lower *δ*^13^C values than plant-based diets. Whenever possible, these teeth were not included in the sample to avoid the possible influence of the suckling effect.

### S2.2 *δ*^13^C as proxy for diet and precipitation

To assess dietary patterns and compare the ecological niches of different species in standardized terms, it is necessary to account for the enrichment factor (*ε**), which represents the isotopic offset between the *δ*^13^C values recorded in the enamel (*δ*^13^C_VPDB_) and those derived from the diet (*δ*^13^C_diet_) ^50–52^. Since this offset varies depending on an animal’s digestive physiology and body mass, a species-specific approach is required. To achieve this, we first applied the body-mass calibrated regression models proposed by ^51^ to estimate *ε** for each taxon. For foregut fermenters such as bovids and cervids, we used the equation *ε** = e^[2.34 + 0.05(BM)], whereas for hindgut fermenters, including equids, rhinocerotids, hippopotami, and rodents we applied *ε** = e^[2.42 + 0.032(BM)]. For body mass values (BM), we used the species-specific values estimated for Levantine taxa in ^53^. The resulting enrichment factors were then inverted (e^x) to obtain the appropriate ‰ values used in the interpretation of stable isotope data. In addition to this body-mass regression approach, we also applied the enrichment factor estimates proposed by ^52^, which were derived from breath-diet isotope measurements across a wide range of mammalian taxa. Unlike body-mass regressions, this method provides fixed *ε** values for more refined digestive physiologies, thereby reducing uncertainties associated with fossil biomass estimates. The enrichment factors used are: ruminants (cervids and bovids, *ε** = 14.51 ‰), non-ruminant foregut fermenters (rhinoceros, hippopotamus, and equids, *ε** = 12.73 ‰), and coprophagous hindgut fermenters (rodents, *ε** = 12.47 ‰). Given the inherent uncertainties in fossil body-mass reconstructions, the enrichment factors proposed by Cerling et al.^52^ were ultimately preferred for data for further analysis and interpretation, while results from both approaches are reported in Table S2 for reference.

This approach allowed for the reconstruction of past vegetation structure by distinguishing between open and closed environments. *δ*^13^C_diet_ values greater than -27‰ suggest open landscapes such as grasslands and parklands, whereas values lower than -27‰ are indicative of denser vegetation cover, including closed-canopy forests ^21,54^.

To further explore environmental conditions, we estimated mean annual precipitation (MAP) using *δ*^13^C_VPDB_-derived dietary signatures. Following Lécuyer et al. ^25^, *δ*^13^C_diet_ values (obtained following Cerling et al ^52^) were used to estimate the plant discrimination factor (Δ^13^C_pl_) that more closely reflect the isotopic composition of local vegetation using the equation Δ^13^C_pl_ = (*δ*^13^C_atm_ - *δ*^13^C_diet_) / [1 + (*δ*^13^C_diet_ / 1000)]. Although variation in atmospheric *δ*^13^C_atm_ between 400 and 100 ka ranges from approximately -7.0 to -6.4‰ ^55,56^, we adopted this intermediate value of -6.8‰ given the existing uncertainties on the numerical age results obtained for the studied assemblages. Using Δ^13^C_pl_ values, we then applied the least-square regression model developed by ^57^ based on the dataset from Kohn ^17^:

Log₁(MAP + 300) = 0.092(±0.004) × Δ^13^C_pl_ + 1.148(±0.074)

This calculation provides a preliminary estimate of precipitation patterns, which must be cross-validated with other environmental proxies due to associated uncertainties of ±100 to 200 mm. These values were then compared to modern estimates were obtained from www.tutiempo.it (location Beirut airport), and mean annual precipitation data averaged from 2000 to 2020.

Although these estimations provide valuable insight into past climate conditions, they should be interpreted as an approximation, and in conjunction with other paleoenvironmental data.

### S2.3 Inverse modelling applied to intratooth profiles

The serial sampling of herbivore enamel yields intratooth *δ*^18^O_VPDB_ profiles that are inherently time-averaged due to the prolonged and multi-phase process of amelogenesis ^58^. Mineralization occurs in overlapping stages of apposition and maturation, meaning that each sample integrates isotopic input over extended periods rather than recording discrete biological increments. As a result, original *δ*^18^O signals are dampened in amplitude and shifted in the position of seasonal peaks and troughs, limiting the accuracy of reconstructed seasonal temperatures ^35,37,58^. To correct for this damping effect, we applied the R implementation of an inverse modelling approach ^58^developed by Pederzani et al. ^35^ (comprehensive guide on the model and how to run it in R can be found in the online repository https://github.com/scpederzani/Oxygen_Inverse_Model), which reconstructs the original *δ*^18^O input by accounting for enamel growth parameters and sampling geometry.

The model requires species-specific values for enamel growth, including initial mineral content, appositional length, and maturation length. Following Pederzani et al. ^35,37^ we used published parameters for large herbivores: *Bos/Bison* (initial mineral content = 25%, appositional length = 5 mm, maturation length = 12 mm) ^58^, *Equus* (initial mineral content = 25%, appositional length = 7 mm, maturation length = 17 mm) ^59^, and caprines (initial mineral content = 25%, appositional length = 3 mm, maturation length = 8 mm) ^44,60,61^. The models also required sampling depth and spacing for each specimen. These were determined by producing positive casts of the sampled teeth using polyvinylsiloxane molding material (Coltene President Microsystem regular body), which were then cut in half and scanned to measure depth and spacing with digitally using the ImageJ software (https://imagej.org). Model error was assessed by adjusting measured (E_meas_) versus predicted variance (E_pred_), yielding damping factors between 0.00001 and 0.0001 for the analyzed specimens.

By anchoring seasonal extrema to the positions of peaks and troughs in the raw *δ*^18^O profiles, this approach minimizes artefacts that may arise from assuming idealized sinusoidal cycles. It also permits inter-tooth and inter-species comparisons, as the relative seasonal shifts are preserved even if absolute amplitudes are partially underestimated ^35^. While originally developed for continuously growing teeth, the model does not fully accommodate the slowing of enamel extension near the enamel-root junction in large ungulates such as horses. Consequently, reconstructed amplitudes for the *Equus* will likely represent minimum estimates ^35,37^.

Modelled summer peaks and winter troughs of *δ*^18^O values were then converted into *δ*^18^O_dw_ (drinking water) estimates using established paleohydrological fractionation equations (e.g., (Kohn et al., 1996)). These *δ*^18^O_dw_ values were subsequently used to reconstruct absolute seasonal temperatures following the empirical relationships between *δ*^18^O_dw_ and ambient temperature for large ungulates (see S2.4.2). For specimens exhibiting non-sinusoidal or incomplete *δ*^18^O profiles, temperature reconstructions were interpreted cautiously, and only well-defined seasonal cycles were used for robust palaeoclimate inference.

### S.2.4 *δ*^18^O as a proxy for seasonal temperature estimation

In this study, intratooth *δ*^18^O_VPDB_ profiles from aurochs, as large-bodied obligate drinkers, were used as the primary source for reconstructing seasonal fluctuations and mean annual temperatures. Since *δ*^18^O in precipitation is inversely correlated with altitude and can vary with water sourcing (e.g., springs and groundwater reservoirs), accounting for both elevation and hydrological effects is essential when interpreting enamel values in regions with complex topography ^34,62^. Enamel-derived *δ*^18^O_dw_ (drinking water) estimates (see 2.4.2) were compared to modern precipitation values at different elevations and spring-water datasets from Lebanon ^63^.

Stable oxygen isotopes in meteoric water, primarily derived from rainfall, show a well-established correlation with mean air temperature in mid- to high latitudes on regional and local scales ^32,33^. Obligate drinkers such as bovines and equids assimilate this water, recording its isotopic composition in tooth enamel with a species-specific offset ^34^. Linking air temperature to precipitation *δ*^18^O, and precipitation *δ*^18^O to enamel *δ*^18^O forms the basis of palaeotemperature reconstructions from faunal remains.

In this study, enamel *δ*^18^O_VSMOW_ values were first converted to the phosphate fraction (*δ*^18^O_phos_, VSMOW), which is more widely used in palaeotemperature calibrations due to its greater resistance to diagenetic alteration and the wider availability of enamel-water calibration datasets ^34,35,37,64^. The conversions followed established equations:

Carbonate → Phosphate = *δ*^18^O_phos_ (VSMOW) = 0.941 × *δ*^18^O_VSMOW_ -7.16 ^65–67^.

Species-specific regressions were then used to estimate meteoric water *δ*^18^O (*δ*^18^O_mw_) from enamel *δ*^18^O_phos_. Following recent compilations ^35,67^, we applied:

(1) Equids: *δ*^18^O_mw_ = (*δ*^18^O_phos_ -22.14) / 0.62
(2) Bovines: *δ*^18^O_mw_ = (*δ*^18^O_phos_ -22.36) / 0.78

Palaeotemperatures were then reconstructed from *δ*^18^O_mw_ using geographically calibrated regression equations relating precipitation *δ*^18^O to mean, summer, and winter air temperatures ^35,37,68^. These regressions were established using available GNIP records from the coastal central Levant. These included the following stations: Yohmor, Tyr, Tripoli, Har Kna'an (Tirat Yael), Tartous, Shaileh, Creen, Bhamdoun, Beyrouth, Bekaa, Bab-Janet, Aramta. The GNIP dataset was complemented with monthly isotopic estimates from the OIPC database (https://wateriso.utah.edu/) and temperature data from WorldClim (1980-2000 baseline; https://www.worldclim.org/). Because GNIP coverage was limited to winter months, OIPC was used to derive monthly *δ*^18^O_precip_ estimates at the same stations, allowing selection of the coldest (typically December-January) and warmest (typically July-August) months for calibration.

We did not apply an explicit correction for glacial-interglacial changes in global seawater *δ*^18^O, because the absolute ages of most assemblages are insufficiently resolved to confidently assign them to specific sea-level states. This introduces an additional uncertainty on the order of ≲1‰ in *δ*^18^O_precip_ (and a few °C in temperature), which is comparable the propagated errors. For this reason, we emphasise relative offsets between sites and between fossil and modern conditions rather than the exact absolute temperature values.

Temperature estimates were calculated following the workflow outlined in Pederzani et al. ^35,37^, using the accompanying R implementation and Excel templates (https://github.com/scpederzani/Isotope_Temperature_Calibration). Compound uncertainties were propagated across both regression steps (enamel-water and water-temperature), following best practice guidelines ^68,69^.

### S2.5 Aridity Index

To evaluate long-term changes in environmental aridity, we compared the *δ*^18^O _VSMOW_ composition of tooth enamel between evaporation-sensitive (ES) and evaporation-insensitive (EI) taxa. ES species are characterized by *δ*^18^O enrichment relative to local meteoric water, often due to consumption of evaporatively enriched leaf water. In contrast, EI species, typically large-bodied, obligate drinkers, record enamel *δ*^18^O values that more closely reflect local meteoric water ^31,70^. Here, we used the stable isotope aridity index developed by Levin et al. ^31^ and refined by Blumenthal et al. ^70^, developed on the principle that species differ in how their enamel *δ*^18^O responds to environmental water, depending on their water-use strategies (EI vs. ES), and their isotopic difference reflect the intensity of the environmental aridity.

In our dataset, aurochs, rhinoceros, hippopotamus, and equids were classified as EI taxa, consistent with their large body size, obligate drinking behavior, and ecological reliance on surface water sources, which minimize evaporative enrichment ^34,70,71^. This classification is supported by the fact that mean *δ*^18^O values of aurochs closely track precipitation values at low altitudes rather than groundwater-fed springs (see Results). By contrast, wild goats displayed *δ*^18^O values enriched relative to EI taxa. Wild and feral goats, including those closely related to *Capra aegagrus*, exhibit strong physiological and behavioral adaptations to water scarcity, often relying on water from forage and tolerating infrequent drinking, especially when moisture-rich forage is available ^72^. Accordingly, we classify wild goats as ES species in our framework, and their elevated *δ*^18^O is interpreted as a consequence of evapotranspiration from plant sources. Given the chronological uncertainties of the Naame - *Niveau Supérieur* specimens, these were excluded from this analysis.

Before calculating the difference between ES and EI species in our dataset, we statistically compared enamel *δ*^18^O values between groups using a Kruskal-Wallis test for each site in order to make sure they were significantly different from each other. We then calculated the difference between mean *δ*^18^O of ES and EI species (ΔES–EI), which quantifies the degree of evaporative enrichment and serves as a proxy for past aridity ^31^. Because our study area covers a geographically restricted region with relatively uniform environmental conditions, we did not calculate enrichment factors against local meteoric water; instead, we interpret ΔES–EI in terms of relative differences between ES and EI taxa (cf.^31,70^).

This analysis was performed using an R script adapted from Luyt et al. ^73^, which incorporated a power analysis to evaluate whether sample size and effect size are sufficient. To assess the robustness of group differences and quantify uncertainty, we applied a bootstrap procedure with 5,000 iterations ^73^. In each iteration, ES and EI datasets were resampled with replacement, and ΔES–EI was recalculated, producing a bootstrap distribution from which 95% confidence intervals were derived.

It is critical to stress that the validity of this approach depends on robust independent evidence of the water-use strategies of the taxa studied and on sufficient sample size ^70,71^. It is equally important to acknowledge its limitations ^71,73^. Therefore, the results should be interpreted as a relative aridity index between the studied sites, and further applications of this analysis are encouraged to build a stronger interpretive framework within the Mediterranean context.

### S2.6. Cross-correlation analysis

To investigate potential seasonal dietary patterns, we analyzed intratooth *δ*^13^C_VBPD_ and *δ*^18^O_VBPD_ profiles across taxa. Because *δ*^13^C reflects dietary inputs and *δ*^18^O primarily records drinking water sources and seasonality, these proxies provide complementary insights ^62^. Examining their relationship allows us to test whether shifts in diet were linked to seasonal changes in water availability or climate. To assess the statistical properties of the data, we first applied the Shapiro-Wilk test to evaluate normality. Where values followed a normal distribution, we used parametric a test as analysis of variance (ANOVA). For non-normally distributed data, we employed non-parametric tests, such as the Kruskal-Wallis test.

## **S3. Stable isotopes extended results**

### S3.1. Preservation screening and data-quality assessment

Carbonate (CO_3_) concentrations across enamel samples from all sites generally fell within the expected range for well-preserved herbivore enamel ^74^ (Table S2), whereas dentine samples consistently exhibited higher carbonate content (Table S3), with no overlap between the two sampled tissues. This confirmed that enamel powders were not contaminated by dentine during sampling.

At Naame, CO_3_ values ranged from 4.6-5.1% in aurochs, 3.8-5.5% in wild goat, 3.2-5.0% in red deer, 4.4-5.7% in fallow deer, 5.1% in equid, 4.1% in porcupine, and 3.6% in rhinoceros (Table S2). Three specimens, two red deer (NME21 and NME38) and one fallow deer (NME6), displayed anomalously high carbonate contents compared to same taxa across collections (6.7%, 6.8%, and 7.4%, respectively) and similarly inconsistent *δ*^18^O values. These were therefore excluded from all subsequent analyses. At Nahr Ibrahim, CO_3_ contents ranged from 4.6-5.2% in aurochs, 4.0-5.8% in wild goat, 3.7-4.2% in fallow deer, and 5.3% in the single hippopotamus specimen. All values fell within the expected enamel range, and no specimens were removed. At Ras el-Kelb, aurochs ranged from 3.2-4.9%, red deer from 4.1-5.0%, the single fallow deer at 5.8%, and rhinoceros from 2.6-4.0%. All samples were retained.

Within sequential intra-tooth series, samples were identified as outliers when they showed pronounced deviation from the fitted polynomial sinusoidal trend of the *δ*^18^O profiles. At Naame, four samples from aurochs NME49 showed exceptionally low carbonate values or failed *δ*^18^O measurements and were removed. Single-sample outliers were also detected and excluded in aurochs NME36 and red deer NME40. At Nahr Ibrahim, one *δ*^18^O outlier was removed from aurochs NIM27. At Ras el-Kelb, individual outlier samples in the intra-tooth series were removed from aurochs REK25, and multiple outlier measurements were removed from aurochs REK45 and rhinoceros REK49 (Supplementary Figure S2 and Table S2), although both specimens were retained with appropriate caution.

Statistical evaluation of inter-species carbonate data across collections in the remaining samples show significant inter-taxon variation (Kruskal–Wallis χ^2^ = 16.20, df = 7, p = 0.0238). Shapiro–Wilk tests detected no deviations from normality –thus, no outliers– except in aurochs REK35 (W = 0.806, p = 0.004). Levene’s test showed no heterogeneity of variances (F(7,47) = 1.022, p = 0.428). Although aurochs REK35 fell marginally outside the 1.5×IQR threshold, its carbonate content remained within the expected enamel range for modern ungulates, and its *δ*^13^C/*δ*^18^O values aligned with other aurochs; the sample was therefore retained.

### S3.2. Sequential isotope profiles

At Naame (levels PIII_2_ and PIII_0_), aurochs (n = 3) exhibited the greatest intra-tooth isotopic variability within the site, with mean *δ*^13^C_VPDB_ range values of 2.8‰ ± 1.4 (±1 SD) and *δ*^18^O_VPDB_ range values of 2.3‰ ± 0.7 (±1 SD). In contrast, wild goat (n = 3) showed more limited variation (*δ*^13^C = 0.7‰ ± 0.3 (±1 SD); *δ*^18^O = 2.3‰ ± 0.9 (±1 SD), while fallow deer (n = 1), red deer (n = 1), and rhinoceros (n = 1) displayed similarly low ranges in both isotopic systems (*δ*^13^C = 0.5 to 0.9‰; *δ*^18^O = 1.6 to 2.2‰).

The aurochs specimen NME-51, from Naame - *Niveau Supérieur* directly dated to MIS 2, exhibited a *δ*^13^C amplitude of 3.4‰ and *δ*^18^O range of 5.5‰, slightly higher than the site mean.

At Nahr Ibrahim, aurochs (n = 5) and wild goat (n = 1) exhibited comparable *δ*^13^C_VPDB_ amplitudes (*δ*^13^C = 1.4 ± 0.6‰ and 1.4‰, respectively), whereas fallow deer (n = 1) showed minimal fluctuation (*δ*^13^C = 0.5‰). Corresponding *δ*^18^O_VPDB_ amplitudes ranged from 2.0‰ in fallow deer to 4.9‰ in wild goat, with aurochs displaying intermediate variability (*δ*^18^O = 2.6 ± 0.9‰). These patterns suggest relatively stable seasonal and dietary conditions for most taxa, with wild goat showing slightly greater oxygen variability, potentially linked to vertical mobility or differing water-use behaviour.

At Ras el-Kelb, intra-tooth isotopic variability was the lowest among the three collections. aurochs (n = 3), red deer (n = 2), and rhinoceros (n = 2) displayed narrow *δ*^13^C_VPDB_ amplitudes (*δ*^13^C = 0.5 to 0.7‰) and limited *δ*^18^O_VPDB_ ranges (*δ*^18^O = 1.1 to 2.0‰), reflecting overall isotopic stability and reduced seasonal fluctuation during enamel formation.

### S3.3. Mean *δ*^13^C results

Mean *δ*^13^C values for all bulk and averaged sequential samples are presented in Table S2. Site-level *δ*^13^C_VPDB_ values at Naame range from –14.7‰ to –9.8‰, representing the broadest variation among the three collections. At Nahr Ibrahim, *δ*^13^C_VPDB_ values range from –12.6‰ to –10.1‰, whereas Ras el-Kelb *δ*^13^C values range from –13.0‰ to –9.7‰.

Within Naame, *δ*^13^C_VPDB_ values vary stratigraphically. Fallow deer from PIII_0_ (n = 8) range from –14.7‰ to –10.9‰ (*δ*^13^C_diet_ = –30.4‰ to –26.6‰), while PIII_2_ specimens (n = 2) range from –12.1‰ to –11.7‰ (–27.8‰ to –27.4‰). Red deer from the *Niveau Supérieur* show values of –13.3‰ (–29.0‰), slightly lower than individuals from PIII_0_ (*δ*^13^C_VPDB_ = –12.0‰ to –11.3‰; *δ*^13^C_diet_ = –27.7‰ to –27.0‰) and PIII_2_ (*δ*^13^C_VPDB_ = –12.6‰; *δ*^13^C_diet_ = -28.3‰). Aurochs from the *Niveau Supérieur* (*δ*^13^C_VPDB_ = –10.2‰; *δ*^13^C_diet_ = –27.8‰) overlapped with PIII_0_ values (*δ*^13^C_VPDB_ = –10.5‰ to -9.8‰; *δ*^13^C_diet_ = –29.9‰ to –26.9‰). The adult wild goat from the *Niveau Supérieur* (*δ*^13^C_VPDB_ = –12.1‰; *δ*^13^C_diet_ = –27.8‰) fell within the PIII_0_ range (*δ*^13^C_VPDB_ = –14.2‰ to –11.2‰; *δ*^13^C_diet_ = –29.9‰ to –26.9‰). The equid (*δ*^13^C_VPDB_ = –11.6‰; *δ*^13^C_diet_ = –25.5‰) and rhinoceros (*δ*^13^C_VPDB_ = –12.2‰; *δ*^13^C_diet_ = –26.1‰) from PIII_0_ displayed intermediate values.

At Nahr Ibrahim, wild goat (n = 4) *δ*^13^C_VPDB_ ranged from –12.1‰ to –11.0 (*δ*^13^C_diet_ = –27.8‰ to -26.7‰), aurochs (n = 6) between –11.4‰ and –10.1‰ (–27.1‰ to –25.8‰), and fallow deer (n = 3) between –12.6‰ and –10.6‰ (–28.3‰ to –26.3‰).

At Ras el-Kelb, aurochs (n = 5) exhibited *δ*^13^C_VPDB_ values from –11.4‰ to –9.7‰ (*δ*^13^C_diet_ = –27.2‰ to –25.4‰), red deer (n = 3) showed a very narrow range (*δ*^13^C_VPDB_ = –11.8‰ to –11.7‰; *δ*^13^C_diet_ = –27.5‰ to –27.4‰), and rhinoceros displayed the broadest variation (*δ*^13^C_VPDB_ = –13.0‰ to –11.3‰; *δ*^13^C_diet_ = –26.9‰ to –25.2‰),

### S3.4. Mean *δ*^18^O results and drinking-water reconstruction

Stratigraphic subdivisions at Naame show intrasite variation. In PIII_2_, red deer the *δ^18^*O = –9.2‰ while fallow deer range from –2.3‰ to –1.4‰. In the *Niveau Supérieur*, red deer, aurochs, and wild goat show values of –6.6‰, -5.9‰, and –4‰, respectively. In PIII_0_, aurochs range from –5.2‰ to –2.9‰, the equid value –6.3‰, fallow deer –5.5‰ to –1.7‰, wild goat –3.7‰ to –1.5‰, and rhinoceros –5.42‰. The highest value in the entire dataset is recorded in a wild goat deciduous tooth (dp4) from the *Niveau Supérieur* (+0.2‰), which is likely a consequence of the enriching weaning effect on this tooth; therefore, this specimen was excluded from further analysis.

At Nahr Ibrahim, aurochs range from –6.0‰ to –3.3‰, hippopotamus show low values of –4.6‰, fallow deer range from –4.6‰ to –1.7‰, and wild goat from –3.0‰ to –0.5‰. At Ras el-Kelb, rhinoceros returned the lowest values (–7.5‰ to –5.7‰), aurochs ranged from –7.3‰ to –4.5‰, red deer from –4.9‰ to –1.5‰, and the single fallow deer specimen measured –0.9‰.

Inverse-modelled *δ^18^*O_VSMOW_ mean values reveal generally strong sinusoidal curves in the bovids. However, the *Equus* specimen (NME43) exhibit an anomalously large range and was excluded from the paleoclimatic reconstructions (Table S5, Fig S3).

As obligate drinkers, aurochs rely heavily on liquid water intake making it a reliable proxy to track local hydrological and climatic conditions. The reconstructed drinking water (*δ^18^*O_dw_) values for aurochs (Table S6) range from –8.0‰ to –4.6‰ at Nahr Ibrahim, between –7.1‰ and –3.6‰ at Naame (PIII_2_ and PIII_0_), and measure –9.4‰ in the MIS 2 *Niveau Supérieur* specimen. Ras el-Kelb aurochs values range from –9.7‰ to –6.1‰.

These values were compared with modern precipitation and spring-water datasets from Lebanon ^63^ to track watering source near each site. Although regional springs, recharged from high-altitude infiltration, display isotopically depleted values, mean aurochs *δ^18^*O aligns more closely with low-altitude precipitation (Fig S4). A distance analysis further supports this interpretation: *δ^18^*O values for all three sites are closer to the modern annual precipitation mean (–5.65‰) than to the spring-water mean (–7.74‰). However, the error on the *δ^18^*O_dw_ calculated from Naame and Ras el-Kelb partially overlap the depleted range of spring water, suggesting occasional or mixed use of these sources. Building on these results, mean *δ^18^*O values of aurochs were further used to estimate Pleistocene meteoric water values (*δ^18^*O_mw_) through species-specific correlations (Table S6). At Nahr Ibrahim, *δ^18^*O_mw_ values ranged from –8.0‰ to –4.6‰, while Naame (levels PIII_2_ and PIII_0_) returned slightly higher values, between –7.1‰ and –3.6‰. In contrast, the specimen from Naame - *Niveau Supérieur*, directly dated to MIS 2, yielded markedly lower values of –9.4‰, indicating very distinct climate conditions. The Ras el-Kelb sample also displayed a relatively low range (–9.7‰ to –6.1‰) compared to Naame and Nahr Ibrahim. Although absolute values differ among the three collections, the intra-site variability consistently averages around 3.5‰ *δ^18^*O_mw_.

### S3.5. Relative aridity index

Sample sizes for the aridity index analysis were sufficient at Naame (levels PIII_2_ and PIII_0_; evaporation-insensitive [EI] = 4, evaporation-sensitive [ES] = 5) and Nahr Ibrahim (EI = 7, ES = 4). Power analyses indicate that, given the observed effect sizes and variability, a minimum of four samples per group is adequate to detect significant isotopic separation with acceptable statistical power. At both sites, variability in *δ*^18^O _VSMOW_ values between EI and ES groups was statistically comparable, as indicated by non-significant Levene’s tests for homogeneity of variance (Naame: F_1,7_ = 0.35, *p* = 0.57; Nahr Ibrahim: F_1,9_ = 0.02, p = 0.88). Non-parametric Wilcoxon rank-sum tests revealed significant differences in *δ*^18^O between EI and ES specimens at both sites (Naame: W = 1, *p* = 0.037; Nahr Ibrahim: W = 0, *p* = 0.011). Effect size estimates (Cohen’s d) were large and negative (Naame = -2.20; Nahr Ibrahim = -3.20), indicating substantially lower *δ*^18^O values in EI relative to ES taxa. The greatest isotopic mean difference between EI and ES species occurred at Nahr Ibrahim (ΔES-EI = 3.04 ‰), where within-group variation was low (ES = 1.04‰; EI = 0.85‰) (Fig. 5B). At Naame, the ΔES-EI mean difference was 2.57‰, with standard deviations of 0.81‰ (ES) and 1.44‰ (EI), suggesting more humid conditions during MIS 5 in the central Levant. Bootstrap-derived 95% confidence intervals confirmed the robustness of these differences (Naame = 0.90 to 3.87‰; Nahr Ibrahim = 1.78 to 4.12‰).

## **S4. Supplementary Figures**

**Figure S1. Example of sequentially sampled tooth.** Samples were collected along the enamel growth axis, starting from the occlusal surface (earliest formed enamel) to the enamel–root junction (latest formed enamel), to reconstruct a time-series record of isotopic variation.

**Figure S2. Intra-tooth carbon and oxygen isotope profiles for individual herbivores from Naame (NME), Nahr Ibrahim (NIM) and Ras el-Kelb (REK).** Sequential enamel samples plotted against distance from the enamel-dentine junction (EDJ) show δ^13^C_VPDB_ (orange), δ^18^O_VPDB_ (blue) values (‰) and detected outliers (grey). Each panel represents one tooth, labeled by specimen ID and taxon. The polynomial trendline illustrate sinusoidal isotopic variation through enamel formation, reflecting seasonal changes in diet and water intake. Element, correlation coefficients (r^2^), and inferred time lags (in months) between δ^13^C and δ^18^O signals are indicated in each plot.

**Figure S3. Inverse-modelled enamel *δ*^18^O_VSMOW_ profiles compared to measured *δ*^18^O values.** Sequential *δ*^18^O_VSMOW_ data (blue points) plotted against the modelled *δ*^18^O input function (most likely solutions shown in the purple line and 95% confidence in the shaded band) for each specimen. Sinusoidal modelled profiles indicate semi-annual isotopic cycles consistent with climatic seasonality.

**Figure S4. Comparison of estimated aurochs drinking-water *δ*^18^O values with modern precipitation and spring-water data from Lebanon.** Mean and SD *δ*^18^O_dw_ values derived from aurochs enamel at Naame (ca. 12 m above the sea level [asl]), Nahr Ibrahim (ca. 15 m asl), and Ras el-Kelb (ca. 20 m asl) are plotted against modern *δ*^18^O_VSMOW_ datasets of precipitation and spring waters at different elevations m asl ^63^.

**Figure S5. Modern seasonal temperature and precipitation patterns for coastal Lebanon.** Mean monthly temperature (top) and precipitation (bottom) values illustrate the modern annual climatic cycle, with warm, dry summers and cool, wet winters typical of a Mediterranean climate. Data was extracted from the WorldClim 2.1 database (1980-2000) using the geodata::worldclim_global function (resolution = 10 arc-minutes) in Rstudio. Climate rasters for mean monthly air temperature (tavg) and precipitation (prec) were cropped to the coastal Lebanon extent (longitude 35-36°E, latitude 33-34.7°N).

**Figure S6. Comparison of δ^18^O values in enamel carbonate between modern domestic goats and Pleistocene wild goats from the Levant.** Mean δ^18^O_VSMOW_ values with 95% confidence intervals (2σ) are shown for modern domesticated goat (Capra hircus) from Carmel Hai Bar (n=4) (left) and for wild goat (Capra aegagrus) from Pleistocene sites including Amud (n=14), Qafzeh L (lower levels, n=2), Qafzeh U (upper levels, n=14), Naame Niveau Supérieur (n=1), Naame PIII_0_ (n=5), and Nahr Ibrahim (n=3). The higher δ^18^O values observed at Amud suggest warmer and/or drier local conditions compared to the other Pleistocene sites, while overall differences between modern domestic and fossil wild goats reflect contrasting drinking behaviors linked to domestication and environmental variability. Data for Carmel Hai Bar, Amud, and Qafzeh are from Hallin et al. ^75^.

## **S5. Supplementary Tables**

**Table S1. Number of teeth sampled by species and assemblages with associated dates.**

| **Site** | Naame - *Niveau Supérieur* | Naame - PIII_2_ | Naame - PIII_0_ | Nahr Ibrahim | Ras el-Kelb |  |
| --- | --- | --- | --- | --- | --- | --- |
| **Age (ka) ^(4)^** | 17.6 ± 2.7; 107 ± 13 | ~ 100 | ~ 100 | 245 ± 23 | 402 ± 46 |  |
| **Species** |  |  |  |  |  | **Total Teeth** |
| *Stephanorhinus hemitoechus* |  |  | 1 |  | 4 | **5** |
| *Hippopotamus amphibius* |  |  |  | 1 |  | **1** |
| *Bos primigenius* | 1 | 1 | 2 | 6 | 5 | **15** |
| *Equus* sp. |  |  | 1 |  |  | **1** |
| *Capra aegagrus* | 2 |  | 5 | 4 |  | **11** |
| *Cervus elaphus* | 2 | 1 | 3 |  | 3 | **9** |
| *Dama mesopotamica* |  | 2 | 9 | 3 | 1 | **15** |
| *Hystrix* sp. | 1 |  |  |  |  | **1** |
| **Total Teeth** | **6** | **4** | **21** | **14** | **13** | **58** |

**Table S2.** **Stable carbon and oxygen isotope results from tooth enamel of mammalian specimens from Naame, Nahr Ibrahim, and Ras el-Kelb.** Minimum, maximum, range, mean, and standard deviation (SD) values for *δ*^13^C_VPDB_ and *δ*^18^O_VSMOW_, and mean values of *δ*^18^O_VPDB_ and carbonate content for each specimen. Dietary *δ*^13^C values were calculated using both the body-mass-calibrated enrichment factors (*ε**) of Tejada-Lara et al. ^51^ and Cerling et al. ^52^. (*) Represent directly dated specimens.

|  |  |  |  |  |  | ***δ*¹³C_VPDB_** | | | | | **Cerling et al. 2021** | **Tejada-Lara et al. 2018** | ***δ*¹⁸O_(VPDB)_** | ***δ¹⁸*O_VSMOW_** | | | | | **CO3 (%)** |
| --- | --- | --- | --- | --- | --- | --- | --- | --- | --- | --- | --- | --- | --- | --- | --- | --- | --- | --- | --- |
| **ID** | **Site** | **Taxon** | **Element** | **Enamel** | **N** | **Min** | **Max** | **Range** | **Mean** | **SD** | ***δ*¹³C_diet_ (‰)** | ***δ*¹³C_diet_ (‰)** | **Mean** | **Min** | **Max** | **Range** | **Mean** | **SD** | **Mean** |
| NIM15 | Nahr Ibrahim | *Capra aegagrus* | M3 | Intra | 8 | -12.0 | -10.4 | 1.6 | -11.3 | 0.6 | -27.0 | -23.8 | -1.3 | 27.1 | 31.3 | 4.3 | 29.6 | 1.6 | 4.3 |
| NIM16 | Nahr Ibrahim | *Capra aegagrus* | M3 | Intra | 11 | -12.9 | -11.5 | 1.4 | -12.1 | 0.5 | -27.8 | -24.6 | -3.0 | 25.5 | 30.5 | 5.0 | 27.9 | 1.7 | 4.5 |
| NIM17 | Nahr Ibrahim | *Capra aegagrus* | M3 | bulk |  |  |  |  | -12.0 |  | -27.7 | -24.6 | -0.5 |  |  |  | 30.4 |  | 5.8 |
| NIM18 | Nahr Ibrahim | *Capra aegagrus* | M1 or 2 | bulk |  |  |  |  | -11.0 |  | -26.7 | -23.5 | -1.3 |  |  |  | 29.5 |  | 4.0 |
| NIM2* | Nahr Ibrahim | *Bos primigenius* | M2 | bulk |  |  |  |  | -10.8 |  | -26.5 | -24.8 | -6.0 |  |  |  | 24.8 |  | 5.0 |
| NIM25 | Nahr Ibrahim | *Bos primigenius* | m2 | Intra | 22 | -11.8 | -10.0 | 1.8 | -10.9 | 0.6 | -26.6 | -24.9 | -4.5 | 25.0 | 27.7 | 2.7 | 26.3 | 0.8 | 5.0 |
| NIM26 | Nahr Ibrahim | *Bos primigenius* | m2 | Intra | 21 | -11.1 | -9.2 | 1.9 | -10.1 | 0.5 | -25.8 | -24.1 | -4.4 | 24.9 | 28.1 | 3.1 | 26.3 | 1.0 | 4.6 |
| NIM27 | Nahr Ibrahim | *Bos primigenius* | m3 | Intra | 12 | -12.3 | -10.9 | 1.4 | -11.4 | 0.5 | -27.1 | -25.4 | -5.1 | 25.0 | 26.3 | 1.3 | 25.7 | 0.4 | 5.2 |
| NIM28 | Nahr Ibrahim | *Bos primigenius* | m3 | Intra | 9 | -10.9 | -10.4 | 0.5 | -10.6 | 0.2 | -26.3 | -24.6 | -3.3 | 26.4 | 28.6 | 2.2 | 27.5 | 0.9 | 4.8 |
| NIM3 | Nahr Ibrahim | *Bos primigenius* | M3 | Intra | 17 | -11.1 | -9.8 | 1.3 | -10.4 | 0.5 | -26.1 | -24.4 | -4.0 | 25.3 | 28.9 | 3.7 | 26.8 | 1.2 | 4.7 |
| NIM36 | Nahr Ibrahim | *Dama mesopotamica* | p4 | Intra | 3 | -12.0 | -11.5 | 0.5 | -11.7 | 0.3 | -27.4 | -24.5 | -2.5 | 27.2 | 29.3 | 2.1 | 28.3 | 1.1 | 3.9 |
| NIM37 | Nahr Ibrahim | *Dama mesopotamica* | m2 | bulk |  |  |  |  | -10.6 |  | -26.3 | -23.3 | -1.7 |  |  |  | 29.2 |  | 3.7 |
| NIM41 | Nahr Ibrahim | *Dama mesopotamica* | m2 | bulk |  |  |  |  | -12.6 |  | -28.3 | -25.3 | -4.6 |  |  |  | 26.2 |  | 4.2 |
| NIM45 | Nahr Ibrahim | *Hippopotamus amphibius* | incisor fragment | bulk |  |  |  |  | -11.8 |  | -25.7 | -25.9 | -4.6 |  |  |  | 26.1 |  | 5.3 |
| NME10 | Naame PIII_0_ | *Capra aegagrus* | m3 | Bulk |  |  |  |  | -11.7 |  | -27.4 | -24.2 | -2.1 |  |  |  | 28.8 |  | 5.0 |
| NME11 | Naame PIII_0_ | *Capra aegagrus* | m3 | Intra | 5 | -11.5 | -11.0 | 0.5 | -11.2 | 0.2 | -26.9 | -23.7 | -3.7 | 26.4 | 27.8 | 1.3 | 27.1 | 0.5 | 5.3 |
| NME15 | Naame PIII_0_ | *Capra aegagrus* | M3 | Intra | 6 | -12.2 | -11.1 | 1.2 | -11.7 | 0.5 | -27.4 | -24.2 | -2.7 | 27.1 | 29.6 | 2.5 | 28.1 | 1.0 | 5.3 |
| NME16 | Naame PIII_0_ | *Capra aegagrus* | M3 | Bulk |  |  |  |  | -12.3 |  | -28.0 | -24.8 | -2.1 |  |  |  | 28.7 |  | 3.8 |
| NME19 | Naame *Niveau Supérieur* | *Cervus elaphus* | M2 | Bulk/buccal |  |  |  |  | -13.3 |  | -29.0 | -26.2 | -6.6 |  |  |  | 24.1 |  | 4.7 |
| NME2* | Naame PIII_0_ | *Cervus elaphus* | p4 | Bulk |  |  |  |  | -12.0 |  | -27.7 | -24.9 | -4.6 |  |  |  | 26.2 |  | 3.2 |
| NME20* | Naame *Niveau Supérieur* | *Capra aegagrus* | M3 | Intra | 8 | -12.4 | -11.8 | 0.5 | -12.1 | 0.2 | -27.8 | -24.6 | -4.0 | 25.1 | 28.3 | 3.2 | 26.8 | 1.2 | 5.3 |
| NME21 | Naame *Niveau Supérieur* | *Cervus elaphus* | M3 | Bulk |  |  |  |  | -11.6 |  |  |  | -1.1 |  |  |  | *30.9* |  | ***6.7*** |
| NME22 | Naame PIII_0_ | *Dama mesopotamica* | m2 | Bulk |  |  |  |  | -12.5 |  | -28.2 | -25.2 | -5.2 |  |  |  | 25.5 |  | 4.9 |
| NME23 | Naame PIII_0_ | *Dama mesopotamica* | M3 | Bulk |  |  |  |  | -12.1 |  | -27.8 | -24.8 | -2.9 |  |  |  | 27.9 |  | 5.2 |
| NME24 | Naame PIII_0_ | *Dama mesopotamica* | M3 | Bulk |  |  |  |  | -11.4 |  | -27.1 | -24.1 | -2.6 |  |  |  | 28.2 |  | 5.7 |
| NME25 | Naame PIII_0_ | *Dama mesopotamica* | m2 | Bulk |  |  |  |  | -14.7 |  | -30.4 | -27.4 | -3.0 |  |  |  | 27.8 |  | 5.4 |
| NME26 | Naame PIII_0_ | *Dama mesopotamica* | M2 | Bulk |  |  |  |  | -10.9 |  | -26.6 | -23.6 | -3.6 |  |  |  | 27.2 |  | 4.4 |
| NME29 | Naame *Niveau Supérieur* | *Hystrix* sp. | P | Bulk |  |  |  |  | -13.8 |  | -26.2 | -25.7 | -5.4 |  |  |  | 25.4 |  | 4.1 |
| NME31 | Naame PIII_0_ | *Capra aegagrus* | M2 | Bulk |  |  |  |  | -14.2 |  | -29.9 | -26.7 | -1.5 |  |  |  | 29.4 |  | 5.0 |
| NME33 | Naame PIII_0_ | *Stephanorhinus hemitoechus* | M2 | Intra | 17 | -12.4 | -11.9 | 0.5 | -12.2 | 0.1 | -26.1 | -26.3 | -5.4 | 24.4 | 26.1 | 1.6 | 25.3 | 0.5 | 3.6 |
| NME35 | Naame PIII_0_ | *Bos primigenius* | M3 | Intra | 14 | -12.2 | -7.3 | 4.9 | -10.5 | 1.7 | -26.2 | -24.5 | -5.2 | 23.9 | 27.3 | 3.4 | 25.5 | 1.0 | 5.3 |
| NME36 | Naame PIII_0_ | *Bos primigenius* | M1 or 2 | Intra | 13 | -11.2 | -9.1 | 2.2 | -9.8 | 0.8 | -25.5 | -23.9 | -2.9 | 26.9 | 28.8 | 1.9 | 27.9 | 0.7 | 5.0 |
| NME38 | Naame PIII_0_ | *Cervus elaphus* | M2 | Bulk |  |  |  |  | -12.8 |  |  |  | -0.1 |  |  |  | 30.8 |  | ***6.8*** |
| NME40 | Naame PIII_0_ | *Cervus elaphus* | m3 | Intra | 6 | -11.6 | -11.1 | 0.5 | -11.3 | 0.2 | -27.0 | -24.2 | -3.7 | 26.1 | 28.3 | 2.2 | 27.1 | 0.9 | 5.0 |
| NME43 | Naame PIII_0_ | *Equus* sp. | m or p | Intra | 19 | -12.2 | -11.1 | 1.1 | -11.6 | 0.4 | -25.5 | -24.8 | -6.3 | 23.2 | 25.4 | 2.2 | 24.4 | 0.6 | 5.1 |
| NME47 | Naame *Niveau Supérieur* | *Capra aegagrus* | dp4 | Bulk |  |  |  |  | -14.1 |  |  |  | 0.2 |  |  |  | 31.1 |  | 5.5 |
| NME48 | Naame PIII_2_ | *Cervus elaphus* | M2 or 3 | Bulk/buccal |  |  |  |  | -12.6 |  | -28.3 | -25.5 | -9.2 |  |  |  | 21.4 |  | 3.9 |
| NME49 | Naame PIII_2_ | *Bos primigenius* | P2 | Intra | 6 | -11.5 | -10.2 | 1.3 | -10.8 | 0.5 | -26.5 | -24.8 | -2.4 | 27.3 | 29.1 | 1.8 | 28.4 | 0.7 | 4.6 |
| NME5 | Naame PIII_0_ | *Dama mesopotamica* | M2 | Bulk |  |  |  |  | -12.6 |  | -28.3 | -25.3 | -5.5 |  |  |  | 25.2 |  | 5.7 |
| NME51* | Naame *Niveau Supérieur* | *Bos primigenius* | M2 or 3 | Intra | 13 | -11.8 | -8.4 | 3.4 | -10.2 | 1.3 | -25.9 | -24.2 | -5.9 | 22.6 | 28.2 | 5.6 | 24.9 | 1.7 | 5.1 |
| NME52 | Naame PIII_2_ | *Dama mesopotamica* | P4 | Bulk |  |  |  |  | -12.1 |  | -27.8 | -24.8 | -2.3 |  |  |  | 28.5 |  | 4.0 |
| NME6 | Naame PIII_0_ | *Dama mesopotamica* | M2 | Bulk |  |  |  |  | -12.1 |  |  |  | -3.6 |  |  |  | 27.2 |  | ***7.4*** |
| NME61 | Naame PIII_2_ | *Dama mesopotamica* | p2 | Bulk |  |  |  |  | -11.7 |  | -27.4 | -24.5 | -1.4 |  |  |  | 29.5 |  | 5.3 |
| NME7 | Naame PIII_0_ | *Dama mesopotamica* | M2 | Bulk |  |  |  |  | -13.9 |  | -29.6 | -26.6 | -1.7 |  |  |  | 29.2 |  | 4.8 |
| NME8 | Naame PIII_0_ | *Dama mesopotamica* | m1 or 2 | Intra | 5 | -12.7 | -11.8 | 0.9 | -12.2 | 0.4 | -27.9 | -24.9 | -5.0 | 25.1 | 26.8 | 1.6 | 25.8 | 0.6 | 4.7 |
| REK16 | Ras el-Kelb | *Cervus elaphus* | M2 or 3 | Intra | 5 | -12.1 | -11.4 | 0.6 | -11.7 | 0.3 | -27.4 | -24.5 | -4.9 | 24.9 | 26.4 | 1.5 | 25.9 | 0.6 | 4.1 |
| REK17 | Ras el-Kelb | *Dama mesopotamica* | M2 | Bulk |  |  |  |  | -12.6 |  | -28.3 | -25.3 | -0.9 |  |  |  | 30.0 |  | 5.8 |
| REK25 | Ras el-Kelb | *Bos primigenius* | m2 | Intra | 11 | -11.6 | -11.2 | 0.4 | -11.4 | 0.1 | -27.1 | -25.4 | -7.3 | 22.7 | 24.0 | 1.4 | 23.3 | 0.4 | 4.5 |
| REK29 | Ras el-Kelb | *Bos primigenius* | m3 | Intra | 14 | -12.1 | -11.1 | 1.0 | -11.5 | 0.3 | -27.2 | -25.5 | -5.2 | 24.6 | 26.3 | 1.7 | 25.6 | 0.6 | 4.9 |
| REK35 | Ras el-Kelb | *Bos primigenius* | P | Bulk |  |  |  |  | -10.3 |  | -26.0 | -24.3 | -5.1 |  |  |  | 25.7 |  | 3.2 |
| REK38 | Ras el-Kelb | *Bos primigenius* | p4 | Bulk |  |  |  |  | -9.7 |  | -25.4 | -23.7 | -4.5 |  |  |  | 26.3 |  | 4.2 |
| REK41 | Ras el-Kelb | *Cervus elaphus* | M3 | Intra | 6 | -12.1 | -11.4 | 0.7 | -11.8 | 0.3 | -27.5 | -24.7 | -4.5 | 25.3 | 28.0 | 2.7 | 26.2 | 1.1 | 4.8 |
| REK42 | Ras el-Kelb | *Cervus elaphus* | M2 | Bulk |  |  |  |  | -11.8 |  | -27.5 | -24.7 | -1.5 |  |  |  | 29.4 |  | 5.0 |
| REK45 | Ras el-Kelb | *Bos primigenius* | M3 | Intra | 13 | -11.5 | -10.8 | 0.7 | -11.0 | 0.2 | -26.7 | -25.0 | -4.9 | 25.0 | 26.5 | 1.5 | 25.9 | 0.4 | 4.5 |
| REK46* | Ras el-Kelb | *Stephanorhinus hemitoechus* | M | bulk |  |  |  |  | -12.7 |  | -26.6 | -26.8 | -6.0 |  |  |  | 24.7 |  | 2.7 |
| REK47.3 | Ras el-Kelb | *Stephanorhinus hemitoechus* | M or P | bulk |  |  |  |  | -11.3 |  | -25.2 | -25.4 | -7.5 |  |  |  | 23.2 |  | 2.6 |
| REK48 | Ras el-Kelb | *Stephanorhinus hemitoechus* | m | Intra | 12 | -13.1 | -12.8 | 0.3 | -13.0 | 0.1 | -26.9 | -27.1 | -6.9 | 23.2 | 24.5 | 1.3 | 23.8 | 0.4 | 4.0 |
| REK49 | Ras el-Kelb | *Stephanorhinus hemitoechus* | m | Intra | 11 | -13.0 | -12.2 | 0.8 | -12.6 | 0.3 | -26.5 | -26.7 | -5.7 | 24.5 | 25.5 | 0.9 | 25.0 | 0.2 | 3.7 |

**Table S3. Stable carbon and oxygen isotope results from dentine of ungulates from Naame, Nahr Ibrahim, and Ras el-Kelb.** Mean values for *δ*^13^C_VPDB_, *δ*^18^O_VPDB_, *δ*^18^O_VSMOW_, and carbonate content for each specimen. (*) Represent directly dated specimens.

| **ID** | **δ¹³C_VPDB_** | **δ¹⁸O_VPDB_** | **δ¹⁸O_VSMOW_** | **CO3 (%)** |
| --- | --- | --- | --- | --- |
| NIM2_den* | -10.6 | -5.8 | 24.9 | 6.5 |
| NIM3_den | -10.3 | -2.9 | 28.0 | 8.5 |
| NIM15_den | -10.1 | -4.1 | 26.7 | 8.1 |
| NIM26_den | -11.1 | -4.3 | 26.5 | 3.6 |
| NIM27_den | -11.2 | -5.3 | 25.4 | 6.9 |
| NIM28_den | -11.3 | -5.2 | 25.5 | 5.6 |
| NIM45_den | -12.7 | -5.1 | 25.7 | 6.6 |
| NME19_den | -12.6 | -3.8 | 27.0 | 6.6 |
| NME2_den* | -11.4 | -3.8 | 27.0 | 5.6 |
| NME20_den* | -11.1 | -5.0 | 25.8 | 7.4 |
| NME21_den | -12.3 | -3.6 | 27.2 | 7.5 |
| NME24_den | -13.0 | -3.9 | 26.9 | 7.3 |
| NME26_den | -10.9 | -5.3 | 25.5 | 8.0 |
| NME29_den | -9.4 | -3.4 | 27.4 | 5.2 |
| NME33_den | -12.5 | -4.4 | 26.4 | 7.1 |
| NME35_den | -9.0 | -4.2 | 26.6 | 6.2 |
| NME36_den | -10.5 | -4.5 | 26.2 | 9.0 |
| NME40_den | -11.9 | -2.9 | 27.9 | 7.6 |
| NME48_den | -12.8 | -3.0 | 27.9 | 7.5 |
| NME51_den* | -10.3 | -6.2 | 24.5 | 6.9 |
| REK16_den | -11.8 | -4.7 | 26.0 | 7.1 |
| REK25_den | -11.3 | -7.4 | 23.3 | 3.9 |
| REK41_den | -11.3 | -4.7 | 26.1 | 7.7 |
| REK45_den | -12.1 | -4.6 | 26.1 | 7.2 |
| REK46_den* | -11.4 | -6.8 | 23.9 | 6.2 |
| REK47_den | -13.4 | -6.4 | 24.3 | 6.4 |
| REK48_den | -13.5 | -8.3 | 22.4 | 2.9 |
| REK49_den | -9.2 | -7.7 | 23.0 | 10.0 |

**Table S4.** **Individual sequential enamel isotope measurements from several ungulate species sampled from Naame, Nahr Ibrahim, and Ras el-Kelb.** Each row represents a sequentially drilled sample along the enamel growth axis from occlusal surface to enamel-root junction (ERJ). Columns include sample mean isotope ratios of carbon (*δ*^13^C_VPDB_), oxygen (*δ*^18^O_VPDB_ and *δ*^13^C_VSMOW_), mean carbonate content (CO_3_ %), and distance from the ERJ (mm). Bolded values represent identified outliers.

| **ID** | **Site** | **Taxon** | **Element** | ***δ*¹³C _VPDB_** | ***δ*¹⁸O _VPDB_** | ***δ*¹⁸O _SMOW_** | **CO_3_ (%)** | **Distance from ERJ (mm)** |
| --- | --- | --- | --- | --- | --- | --- | --- | --- |
| NIM-16a | Nahr Ibrahim | *Capra aegagrus* | M3 | -12.4 | -1.9 | 28.9 | 4.7 | 33.4 |
| NIM-16b | Nahr Ibrahim | *Capra aegagrus* | M3 | -11.5 | -1.5 | 29.4 | 4.4 | 31.4 |
| NIM-16c | Nahr Ibrahim | *Capra aegagrus* | M3 | -11.7 | -0.4 | 30.5 | 4.8 | 28.6 |
| NIM-16d | Nahr Ibrahim | *Capra aegagrus* | M3 | -11.6 | -1.6 | 29.3 | 4.5 | 25.1 |
| NIM-16e | Nahr Ibrahim | *Capra aegagrus* | M3 | -11.6 | -2.2 | 28.6 | 4.5 | 22.2 |
| NIM-16f | Nahr Ibrahim | *Capra aegagrus* | M3 | -11.7 | -3.4 | 27.5 | 4.4 | 19.2 |
| NIM-16g | Nahr Ibrahim | *Capra aegagrus* | M3 | -12.0 | -4.1 | 26.7 | 4.3 | 16.5 |
| NIM-16h | Nahr Ibrahim | *Capra aegagrus* | M3 | -12.4 | -5.3 | 25.5 | 4.6 | 14.3 |
| NIM-16i | Nahr Ibrahim | *Capra aegagrus* | M3 | -12.9 | -5.2 | 25.5 | 4.4 | 11.7 |
| NIM-16j | Nahr Ibrahim | *Capra aegagrus* | M3 | -12.9 | -4.7 | 26.0 | 4.4 | 8.2 |
| NIM-16k | Nahr Ibrahim | *Capra aegagrus* | M3 | -12.3 | -2.3 | 28.5 | 4.3 | 5.8 |
| NIM-25a | Nahr Ibrahim | *Bos primigenius* | m2 | -11.7 | -5.5 | 25.2 | 5.1 | 57.7 |
| NIM-25b | Nahr Ibrahim | *Bos primigenius* | m2 | -11.8 | -4.9 | 25.8 | 5.0 | 55.0 |
| NIM-25c | Nahr Ibrahim | *Bos primigenius* | m2 | -11.6 | -4.3 | 26.5 | 5.3 | 52.3 |
| NIM-25d | Nahr Ibrahim | *Bos primigenius* | m2 | -11.4 | -4.3 | 26.5 | 5.5 | 49.9 |
| NIM-25e | Nahr Ibrahim | *Bos primigenius* | m2 | -11.4 | -3.9 | 26.9 | 5.5 | 47.6 |
| NIM-25f | Nahr Ibrahim | *Bos primigenius* | m2 | -11.3 | -3.7 | 27.1 | 5.3 | 46.0 |
| NIM-25g | Nahr Ibrahim | *Bos primigenius* | m2 | -11.0 | -3.5 | 27.3 | 5.4 | 43.0 |
| NIM-25h | Nahr Ibrahim | *Bos primigenius* | m2 | -10.8 | -3.1 | 27.7 | 5.0 | 41.0 |
| NIM-25i | Nahr Ibrahim | *Bos primigenius* | m2 | -10.6 | -4.0 | 26.8 | 5.0 | 39.1 |
| NIM-25j | Nahr Ibrahim | *Bos primigenius* | m2 | -10.5 | -3.9 | 26.9 | 5.0 | 37.0 |
| NIM-25k | Nahr Ibrahim | *Bos primigenius* | m2 | -10.3 | -3.6 | 27.2 | 5.1 | 34.0 |
| NIM-25l | Nahr Ibrahim | *Bos primigenius* | m2 | -10.2 | -4.1 | 26.7 | 4.9 | 32.1 |
| NIM-25m | Nahr Ibrahim | *Bos primigenius* | m2 | -10.0 | -4.6 | 26.2 | 4.8 | 29.7 |
| NIM-25n | Nahr Ibrahim | *Bos primigenius* | m2 | -10.1 | -4.4 | 26.4 | 4.8 | 27.4 |
| NIM-25o | Nahr Ibrahim | *Bos primigenius* | m2 | -10.2 | -4.5 | 26.2 | 5.0 | 24.7 |
| NIM-25p | Nahr Ibrahim | *Bos primigenius* | m2 | -10.3 | -5.0 | 25.8 | 4.8 | 22.5 |
| NIM-25q | Nahr Ibrahim | *Bos primigenius* | m2 | -10.5 | -5.2 | 25.6 | 4.7 | 20.3 |
| NIM-25r | Nahr Ibrahim | *Bos primigenius* | m2 | -10.5 | -5.2 | 25.5 | 4.4 | 17.8 |
| NIM-25s | Nahr Ibrahim | *Bos primigenius* | m2 | -10.8 | -5.7 | 25.0 | 4.9 | 15.4 |
| NIM-25t | Nahr Ibrahim | *Bos primigenius* | m2 | -11.0 | -5.7 | 25.0 | 4.8 | 12.2 |
| NIM-25u | Nahr Ibrahim | *Bos primigenius* | m2 | -11.2 | -5.0 | 25.8 | 4.7 | 8.9 |
| NIM-25v | Nahr Ibrahim | *Bos primigenius* | m2 | -11.6 | -4.7 | 26.0 | 4.6 | 5.8 |
| NIM-26a | Nahr Ibrahim | *Bos primigenius* | m2 | -10.0 | -5.5 | 25.2 | 4.3 | 60.1 |
| NIM-26b | Nahr Ibrahim | *Bos primigenius* | m2 | -10.1 | -5.8 | 24.9 | 4.4 | 57.5 |
| NIM-26c | Nahr Ibrahim | *Bos primigenius* | m2 | -9.9 | -5.6 | 25.1 | 4.4 | 54.7 |
| NIM-26d | Nahr Ibrahim | *Bos primigenius* | m2 | -10.1 | -5.3 | 25.4 | 4.6 | 52.5 |
| NIM-26e | Nahr Ibrahim | *Bos primigenius* | m2 | -10.3 | -5.4 | 25.4 | 4.8 | 49.8 |
| NIM-26f | Nahr Ibrahim | *Bos primigenius* | m2 | -10.4 | -5.2 | 25.6 | 4.6 | 47.2 |
| **ID** | **Site** | **Taxon** | **Element** | ***δ*¹³C _VPDB_** | ***δ*¹⁸O _VPDB_** | ***δ*¹⁸O _SMOW_** | **CO_3_ (%)** | **Distance from ERJ (mm)** |
| NIM-26g | Nahr Ibrahim | *Bos primigenius* | m2 | -10.2 | -4.7 | 26.0 | 5.0 | 44.6 |
| NIM-26h | Nahr Ibrahim | *Bos primigenius* | m2 | -10.2 | -4.3 | 26.5 | 4.9 | 41.6 |
| NIM-26i | Nahr Ibrahim | *Bos primigenius* | m2 | -10.1 | -3.9 | 26.8 | 4.7 | 39.3 |
| NIM-26j | Nahr Ibrahim | *Bos primigenius* | m2 | -10.0 | -3.2 | 27.6 | 4.6 | 36.3 |
| NIM-26k | Nahr Ibrahim | *Bos primigenius* | m2 | -9.9 | -2.7 | 28.1 | 4.6 | 33.8 |
| NIM-26l | Nahr Ibrahim | *Bos primigenius* | m2 | -9.8 | -3.3 | 27.5 | 4.4 | 31.3 |
| NIM-26m | Nahr Ibrahim | *Bos primigenius* | m2 | -9.5 | -3.3 | 27.5 | 4.5 | 28.8 |
| NIM-26n | Nahr Ibrahim | *Bos primigenius* | m2 | -9.2 | -3.3 | 27.6 | 4.6 | 26.9 |
| NIM-26o | Nahr Ibrahim | *Bos primigenius* | m2 | -9.3 | -3.6 | 27.2 | 4.7 | 24.2 |
| NIM-26p | Nahr Ibrahim | *Bos primigenius* | m2 | -9.5 | -3.7 | 27.1 | 4.8 | 21.2 |
| NIM-26q | Nahr Ibrahim | *Bos primigenius* | m2 | -10.0 | -4.9 | 25.8 | 4.5 | 18.5 |
| NIM-26r | Nahr Ibrahim | *Bos primigenius* | m2 | -10.3 | -4.9 | 25.8 | 4.7 | 16.1 |
| NIM-26s | Nahr Ibrahim | *Bos primigenius* | m2 | -10.5 | -5.0 | 25.8 | 4.7 | 13.2 |
| NIM-26t | Nahr Ibrahim | *Bos primigenius* | m2 | -10.8 | -4.7 | 26.1 | 4.6 | 10.5 |
| NIM-26u | Nahr Ibrahim | *Bos primigenius* | m2 | -11.1 | -4.9 | 25.9 | 4.4 | 7.2 |
| NIM-27a | Nahr Ibrahim | *Bos primigenius* | m3 | -12.3 | -5.6 | 25.1 | 5.1 | 34.4 |
| NIM-27b | Nahr Ibrahim | *Bos primigenius* | m3 | -12.1 | -5.8 | 25.0 | 5.2 | 31.6 |
| NIM-27c | Nahr Ibrahim | *Bos primigenius* | m3 | -11.8 | -5.3 | 25.4 | 4.9 | 28.7 |
| NIM-27d | Nahr Ibrahim | *Bos primigenius* | m3 | -11.6 | -5.4 | 25.4 | 5.1 | 25.9 |
| ***NIM-27e*** | ***Nahr Ibrahim*** | ***Bos primigenius*** | ***m3*** | ***-11.4*** | ***-6.5*** | ***24.2*** | ***5.0*** | ***23.9*** |
| NIM-27f | Nahr Ibrahim | *Bos primigenius* | m3 | -11.1 | -4.7 | 26.0 | 5.1 | 20.6 |
| NIM-27g | Nahr Ibrahim | *Bos primigenius* | m3 | -11.0 | -4.5 | 26.3 | 5.3 | 18.4 |
| NIM-27h | Nahr Ibrahim | *Bos primigenius* | m3 | -11.0 | -4.7 | 26.1 | 4.9 | 15.9 |
| NIM-27i | Nahr Ibrahim | *Bos primigenius* | m3 | -10.9 | -4.8 | 26.0 | 5.2 | 13.6 |
| NIM-27j | Nahr Ibrahim | *Bos primigenius* | m3 | -11.0 | -5.0 | 25.7 | 5.3 | 11.2 |
| NIM-27k | Nahr Ibrahim | *Bos primigenius* | m3 | -11.1 | -5.2 | 25.6 | 5.2 | 8.9 |
| NIM-27l | Nahr Ibrahim | *Bos primigenius* | m3 | -11.2 | -5.0 | 25.7 | 5.7 | 6.5 |
| NIM-27m | Nahr Ibrahim | *Bos primigenius* | m3 | -11.4 | -4.9 | 25.9 | 5.4 | 4.1 |
| NIM-28a | Nahr Ibrahim | *Bos primigenius* | m3 | -10.9 | -2.2 | 28.6 | 4.9 | 23.0 |
| NIM-28b | Nahr Ibrahim | *Bos primigenius* | m3 | -10.8 | -2.6 | 28.3 | 4.9 | 20.6 |
| NIM-28c | Nahr Ibrahim | *Bos primigenius* | m3 | -10.5 | -2.8 | 28.0 | 4.6 | 18.0 |
| NIM-28d | Nahr Ibrahim | *Bos primigenius* | m3 | -10.5 | -2.6 | 28.2 | 4.6 | 15.6 |
| NIM-28e | Nahr Ibrahim | *Bos primigenius* | m3 | -10.4 | -2.8 | 28.1 | 4.7 | 13.3 |
| NIM-28f | Nahr Ibrahim | *Bos primigenius* | m3 | -10.6 | -3.7 | 27.1 | 4.3 | 11.1 |
| NIM-28g | Nahr Ibrahim | *Bos primigenius* | m3 | -10.5 | -4.0 | 26.8 | 4.9 | 8.6 |
| NIM-28h | Nahr Ibrahim | *Bos primigenius* | m3 | -10.6 | -4.4 | 26.4 | 5.0 | 6.1 |
| NIM-28i | Nahr Ibrahim | *Bos primigenius* | m3 | -10.8 | -4.3 | 26.4 | 5.3 | 3.4 |
| NIM-36a | Nahr Ibrahim | *Dama mesopotamica* | p4 | -12.0 | -1.6 | 29.3 | 3.7 | 8.5 |
| NIM-36b | Nahr Ibrahim | *Dama mesopotamica* | p4 | -11.8 | -2.3 | 28.6 | 3.8 | 5.4 |
| NIM-36c | Nahr Ibrahim | *Dama mesopotamica* | p4 | -11.5 | -3.6 | 27.2 | 4.2 | 2.7 |
| NIM-3a | Nahr Ibrahim | *Bos primigenius* | M3 | -10.9 | -4.6 | 26.2 | 5.0 | 43.7 |
| NIM-3b | Nahr Ibrahim | *Bos primigenius* | M3 | -10.9 | -5.2 | 25.6 | 4.6 | 40.9 |
| NIM-3c | Nahr Ibrahim | *Bos primigenius* | M3 | -10.9 | -5.1 | 25.7 | 5.0 | 38.8 |
| **ID** | **Site** | **Taxon** | **Element** | ***δ*¹³C _VPDB_** | ***δ*¹⁸O _VPDB_** | ***δ*¹⁸O _SMOW_** | **CO_3_ (%)** | **Distance from ERJ (mm)** |
| NIM-3d | Nahr Ibrahim | *Bos primigenius* | M3 | -11.0 | -5.5 | 25.3 | 4.8 | 36.3 |
| NIM-3e | Nahr Ibrahim | *Bos primigenius* | M3 | -11.1 | -4.8 | 26.0 | 4.9 | 33.7 |
| NIM-3f | Nahr Ibrahim | *Bos primigenius* | M3 | -11.0 | -4.7 | 26.0 | 4.8 | 31.5 |
| NIM-3g | Nahr Ibrahim | *Bos primigenius* | M3 | -10.8 | -4.5 | 26.2 | 4.5 | 28.6 |
| NIM-3h | Nahr Ibrahim | *Bos primigenius* | M3 | -10.4 | -3.1 | 27.7 | 4.7 | 26.1 |
| NIM-3i | Nahr Ibrahim | *Bos primigenius* | M3 | -10.4 | -2.4 | 28.4 | 4.8 | 24.4 |
| NIM-3j | Nahr Ibrahim | *Bos primigenius* | M3 | -10.2 | -2.4 | 28.5 | 4.8 | 21.7 |
| NIM-3k | Nahr Ibrahim | *Bos primigenius* | M3 | -10.1 | -1.9 | 28.9 | 4.8 | 19.1 |
| NIM-3l | Nahr Ibrahim | *Bos primigenius* | M3 | -10.0 | -2.7 | 28.1 | 4.4 | 17.0 |
| NIM-3m | Nahr Ibrahim | *Bos primigenius* | M3 | -9.8 | -2.7 | 28.1 | 4.8 | 14.5 |
| NIM-3n | Nahr Ibrahim | *Bos primigenius* | M3 | -9.9 | -4.5 | 26.3 | 4.2 | 12.2 |
| NIM-3o | Nahr Ibrahim | *Bos primigenius* | M3 | -9.9 | -5.0 | 25.8 | 4.3 | 9.8 |
| NIM-3p | Nahr Ibrahim | *Bos primigenius* | M3 | -10.1 | -4.3 | 26.4 | 4.4 | 6.7 |
| NIM-3q | Nahr Ibrahim | *Bos primigenius* | M3 | -10.1 | -3.9 | 26.9 | 5.5 | 3.9 |
| NME11a | Naame | *Capra aegagrus* | m3 | -11.5 | -3.4 | 27.4 | 5.2 | 12.9 |
| NME11b | Naame | *Capra aegagrus* | m3 | -11.3 | -3.1 | 27.8 | 5.2 | 10.2 |
| NME11c | Naame | *Capra aegagrus* | m3 | -11.0 | -3.9 | 26.9 | 5.2 | 7.2 |
| NME11d | Naame | *Capra aegagrus* | m3 | -11.0 | -4.4 | 26.4 | 5.5 | 4.5 |
| NME11e | Naame | *Capra aegagrus* | m3 | -11.3 | -3.5 | 27.3 | 5.3 | 2.6 |
| NME15a | Naame | *Capra aegagrus* | M3 | -12.2 | -1.7 | 29.1 | 5.4 | 18.1 |
| NME15b | Naame | *Capra aegagrus* | M3 | -12.2 | -1.3 | 29.6 | 5.2 | 14.8 |
| NME15c | Naame | *Capra aegagrus* | M3 | -12.1 | -2.9 | 27.9 | 5.1 | 12.3 |
| NME15d | Naame | *Capra aegagrus* | M3 | -11.4 | -3.4 | 27.4 | 5.2 | 9.6 |
| NME15e | Naame | *Capra aegagrus* | M3 | -11.1 | -3.7 | 27.1 | 5.7 | 6.5 |
| NME15f | Naame | *Capra aegagrus* | M3 | -11.3 | -3.2 | 27.6 | 5.2 | 3.6 |
| NME-20a | Naame | *Capra aegagrus* | M3 | -12.2 | -3.7 | 27.1 | 5.6 | 25.0 |
| NME-20b | Naame | *Capra aegagrus* | M3 | -12.4 | -2.8 | 28.0 | 5.2 | 22.2 |
| NME-20c | Naame | *Capra aegagrus* | M3 | -12.1 | -3.4 | 27.4 | 5.2 | 18.5 |
| NME-20d | Naame | *Capra aegagrus* | M3 | -12.3 | -4.1 | 26.7 | 5.1 | 15.6 |
| NME-20e | Naame | *Capra aegagrus* | M3 | -12.3 | -4.9 | 25.9 | 5.5 | 13.2 |
| NME-20f | Naame | *Capra aegagrus* | M3 | -11.9 | -5.6 | 25.1 | 5.0 | 9.4 |
| NME-20g | Naame | *Capra aegagrus* | M3 | -11.8 | -5.2 | 25.6 | 5.3 | 5.4 |
| NME-20h | Naame | *Capra aegagrus* | M3 | -11.9 | -2.5 | 28.3 | 5.8 | 3.1 |
| NME33a | Naame | *Stephanorhinus hemitoechus* | P3 | -12.3 | -5.4 | 25.3 | 3.5 | 45.9 |
| NME33b | Naame | *Stephanorhinus hemitoechus* | P3 | -12.2 | -5.2 | 25.5 | 3.5 | 43.9 |
| NME33c | Naame | *Stephanorhinus hemitoechus* | P3 | -11.9 | -5.1 | 25.6 | 3.2 | 41.7 |
| NME33d | Naame | *Stephanorhinus hemitoechus* | P3 | -11.9 | -4.8 | 25.9 | 3.6 | 39.3 |
| NME33e | Naame | *Stephanorhinus hemitoechus* | P3 | -12.2 | -5.2 | 25.6 | 3.4 | 36.7 |
| NME33f | Naame | *Stephanorhinus hemitoechus* | P3 | -12.2 | -5.3 | 25.4 | 3.6 | 33.8 |
| NME33g | Naame | *Stephanorhinus hemitoechus* | P3 | -12.3 | -5.2 | 25.6 | 3.8 | 30.8 |
| NME33h | Naame | *Stephanorhinus hemitoechus* | P3 | -12.3 | -5.2 | 25.6 | 3.5 | 28.3 |
| **ID** | **Site** | **Taxon** | **Element** | ***δ*¹³C _VPDB_** | ***δ*¹⁸O _VPDB_** | ***δ*¹⁸O _SMOW_** | **CO_3_ (%)** | **Distance from ERJ (mm)** |
| NME33i | Naame | *Stephanorhinus hemitoechus* | P3 | -12.2 | -6.0 | 24.7 | 3.5 | 25.6 |
| NME33j | Naame | *Stephanorhinus hemitoechus* | P3 | -12.2 | -6.3 | 24.4 | 3.3 | 23.1 |
| NME33k | Naame | *Stephanorhinus hemitoechus* | P3 | -12.4 | -6.1 | 24.6 | 3.7 | 20.5 |
| NME33l | Naame | *Stephanorhinus hemitoechus* | P3 | -12.3 | -5.8 | 24.9 | 3.8 | 17.9 |
| NME33m | Naame | *Stephanorhinus hemitoechus* | P3 | -12.4 | -5.3 | 25.4 | 3.8 | 16.1 |
| NME33n | Naame | *Stephanorhinus hemitoechus* | P3 | -12.1 | -5.4 | 25.4 | 3.7 | 13.9 |
| NME33o | Naame | *Stephanorhinus hemitoechus* | P3 | -12.0 | -4.7 | 26.1 | 3.9 | 11.7 |
| NME33p | Naame | *Stephanorhinus hemitoechus* | P3 | -12.1 | -5.3 | 25.5 | 3.7 | 9.1 |
| NME33q | Naame | *Stephanorhinus hemitoechus* | P3 | -12.2 | -5.8 | 24.9 | 4.0 | 6.6 |
| NME35a | Naame | *Bos primigenius* | M3 | -11.6 | -4.8 | 26.0 | 5.4 | 40.2 |
| NME35b | Naame | *Bos primigenius* | M3 | -11.9 | -4.4 | 26.3 | 5.7 | 36.7 |
| NME35c | Naame | *Bos primigenius* | M3 | -12.0 | -5.4 | 25.3 | 5.4 | 33.6 |
| NME35d | Naame | *Bos primigenius* | M3 | -12.1 | -5.9 | 24.9 | 5.3 | 30.8 |
| NME35e | Naame | *Bos primigenius* | M3 | -12.2 | -6.2 | 24.5 | 5.4 | 27.9 |
| NME35f | Naame | *Bos primigenius* | M3 | -12.2 | -6.6 | 24.1 | 5.5 | 25.2 |
| NME35g | Naame | *Bos primigenius* | M3 | -11.5 | -6.8 | 23.9 | 5.0 | 22.4 |
| NME35h | Naame | *Bos primigenius* | M3 | -11.2 | -6.0 | 24.7 | 4.9 | 19.7 |
| NME35i | Naame | *Bos primigenius* | M3 | -10.5 | -5.5 | 25.3 | 5.4 | 16.8 |
| NME35j | Naame | *Bos primigenius* | M3 | -9.6 | -5.1 | 25.6 | 5.1 | 14.3 |
| NME35k | Naame | *Bos primigenius* | M3 | -9.1 | -4.6 | 26.2 | 5.2 | 11.5 |
| NME35l | Naame | *Bos primigenius* | M3 | -8.4 | -4.5 | 26.3 | 5.0 | 8.5 |
| NME35m | Naame | *Bos primigenius* | M3 | -7.9 | -3.9 | 26.8 | 5.0 | 6.1 |
| NME35n | Naame | *Bos primigenius* | M3 | -7.3 | -3.5 | 27.3 | 5.2 | 3.6 |
| NME36a | Naame | *Bos primigenius* | M1 or M2 | -10.9 | -2.0 | 28.8 | 4.9 | 44.0 |
| NME36b | Naame | *Bos primigenius* | M1 or M2 | -10.1 | -2.3 | 28.5 | 4.8 | 40.7 |
| NME36c | Naame | *Bos primigenius* | M1 or M2 | -9.7 | -2.3 | 28.5 | 4.7 | 37.0 |
| NME36d | Naame | *Bos primigenius* | M1 or M2 | -9.3 | -3.2 | 27.6 | 4.8 | 34.6 |
| NME36e | Naame | *Bos primigenius* | M1 or M2 | -9.1 | -3.3 | 27.5 | 5.2 | 31.5 |
| NME36f | Naame | *Bos primigenius* | M1 or M2 | -9.1 | -3.9 | 26.9 | 4.6 | 28.6 |
| NME36g | Naame | *Bos primigenius* | M1 or M2 | -9.1 | -3.5 | 27.3 | 4.9 | 25.7 |
| NME36h | Naame | *Bos primigenius* | M1 or M2 | -9.2 | -3.6 | 27.2 | 5.2 | 22.9 |
| NME36i | Naame | *Bos primigenius* | M1 or M2 | -9.5 | -3.8 | 27.0 | 4.9 | 20.2 |
| NME36j | Naame | *Bos primigenius* | M1 or M2 | -9.7 | -2.8 | 28.1 | 5.4 | 17.2 |
| NME36k | Naame | *Bos primigenius* | M1 or M2 | -10.3 | -2.5 | 28.3 | 5.2 | 14.8 |
| NME36l | Naame | *Bos primigenius* | M1 or M2 | -10.8 | -2.5 | 28.4 | 5.3 | 11.9 |
| ***NME36m*** | ***Naame*** | ***Bos primigenius*** | ***M1 or M2*** | ***-11.2*** | ***-3.4*** | ***27.4*** | ***4.7*** | ***8.8*** |
| NME36n | Naame | *Bos primigenius* | M1 or M2 | -11.2 | -2.5 | 28.3 | 5.2 | 6.0 |
| NME40a | Naame | *Cervus elaphus* | m3 | -11.3 | -2.5 | 28.3 | 4.9 | 17.7 |
| NME40b | Naame | *Cervus elaphus* | m3 | -11.1 | -2.9 | 27.9 | 5.1 | 15.0 |
| ***NME40c*** | ***Naame*** | ***Cervus elaphus*** | ***m3*** | ***-11.1*** | ***-5.7*** | ***25.0*** | ***4.7*** | ***12.5*** |
| NME40d | Naame | *Cervus elaphus* | m3 | -11.2 | -3.6 | 27.2 | 4.7 | 10.5 |
| **ID** | **Site** | **Taxon** | **Element** | ***δ*¹³C _VPDB_** | ***δ*¹⁸O _VPDB_** | ***δ*¹⁸O _SMOW_** | **CO_3_ (%)** | **Distance from ERJ (mm)** |
| NME40e | Naame | *Cervus elaphus* | m3 | -11.1 | -4.7 | 26.1 | 4.6 | 8.4 |
| NME40f | Naame | *Cervus elaphus* | m3 | -11.3 | -4.3 | 26.5 | 5.5 | 5.9 |
| NME40g | Naame | *Cervus elaphus* | m3 | -11.6 | -4.2 | 26.6 | 5.4 | 3.1 |
| NME43a | Naame | *Equus* sp. | Indet. Molar | -11.2 | -5.4 | 25.4 | 5.2 | 64.8 |
| NME43b | Naame | *Equus* sp. | Indet. Molar | -11.2 | -5.7 | 25.0 | 5.2 | 62.2 |
| NME43c | Naame | *Equus* sp. | Indet. Molar | -11.1 | -5.9 | 24.9 | 5.1 | 59.5 |
| NME43d | Naame | *Equus* sp. | Indet. Molar | -11.1 | -5.9 | 24.9 | 4.8 | 56.9 |
| NME43e | Naame | *Equus* sp. | Indet. Molar | -11.1 | -6.3 | 24.4 | 5.1 | 54.8 |
| NME43f | Naame | *Equus* sp. | Indet. Molar | -11.1 | -6.7 | 24.0 | 4.9 | 52.3 |
| NME43g | Naame | *Equus* sp. | Indet. Molar | -11.3 | -6.4 | 24.3 | 5.3 | 49.5 |
| NME43h | Naame | *Equus* sp. | Indet. Molar | -11.4 | -7.5 | 23.2 | 5.1 | 46.2 |
| NME43i | Naame | *Equus* sp. | Indet. Molar | -11.6 | -6.9 | 23.8 | 5.3 | 44.2 |
| NME43j | Naame | *Equus* sp. | Indet. Molar | -11.6 | -6.7 | 24.0 | 5.3 | 41.4 |
| NME43k | Naame | *Equus* sp. | Indet. Molar | -11.9 | -7.3 | 23.4 | 4.9 | 38.9 |
| NME43l | Naame | *Equus* sp. | Indet. Molar | -12.0 | -6.5 | 24.2 | 5.0 | 35.8 |
| NME43m | Naame | *Equus* sp. | Indet. Molar | -12.0 | -6.5 | 24.3 | 4.9 | 33.5 |
| NME43n | Naame | *Equus* sp. | Indet. Molar | -12.2 | -6.5 | 24.2 | 4.9 | 30.8 |
| NME43o | Naame | *Equus* sp. | Indet. Molar | -12.1 | -6.4 | 24.3 | 5.4 | 27.5 |
| NME43p | Naame | *Equus* sp. | Indet. Molar | -11.9 | -6.3 | 24.4 | 5.4 | 24.3 |
| NME43q | Naame | *Equus* sp. | Indet. Molar | -12.0 | -5.7 | 25.0 | 5.2 | 20.4 |
| NME43r | Naame | *Equus* sp. | Indet. Molar | -12.0 | -5.7 | 25.0 | 4.9 | 16.3 |
| NME43s | Naame | *Equus* sp. | Indet. Molar | -12.1 | -5.9 | 24.9 | 4.4 | 12.2 |
| NME49a | Naame | *Bos primigenius* | P2 | -11.5 | -3.1 | 27.7 | 4.9 | 28.4 |
| NME49b | Naame | *Bos primigenius* | P2 | -11.3 | -3.5 | 27.3 | 4.8 | 25.1 |
| ***NME49c*** | ***Naame*** | ***Bos primigenius*** | ***P2*** | ***-14.3*** | ***-16.6*** | ***13.8*** | ***0.0*** | ***23.0*** |
| ***NME49d*** | ***Naame*** | ***Bos primigenius*** | ***P2*** | ***-11.2*** | ***-3.2*** | ***27.6*** | ***4.6*** | ***20.3*** |
| ***NME49e*** | ***Naame*** | ***Bos primigenius*** | ***P2*** | ***-14.8*** | ***-17.7*** | ***12.6*** | ***0.0*** | ***17.7*** |
| ***NME49f*** | ***Naame*** | ***Bos primigenius*** | ***P2*** | ***-10.9*** | ***-2.0*** | ***28.8*** | ***4.6*** | ***15.5*** |
| NME49g | Naame | *Bos primigenius* | P2 | -10.8 | -2.0 | 28.9 | 4.5 | 13.5 |
| NME49h | Naame | *Bos primigenius* | P2 | -10.5 | -2.0 | 28.9 | 4.6 | 12.0 |
| NME49i | Naame | *Bos primigenius* | P2 | -10.3 | -1.7 | 29.1 | 4.4 | 9.4 |
| NME49j | Naame | *Bos primigenius* | P2 | -10.2 | -3.1 | 27.7 | 4.6 | 6.0 |
| NME-51a | Naame | *Bos primigenius* | M3 | -8.8 | -2.6 | 28.2 | 5.6 | 40.6 |
| NME-51b | Naame | *Bos primigenius* | M3 | -8.4 | -3.8 | 27.0 | 5.4 | 37.8 |
| NME-51c | Naame | *Bos primigenius* | M3 | -8.8 | -4.1 | 26.7 | 5.1 | 35.0 |
| NME-51d | Naame | *Bos primigenius* | M3 | -8.9 | -5.3 | 25.4 | 4.8 | 31.6 |
| NME-51e | Naame | *Bos primigenius* | M3 | -9.7 | -6.6 | 24.2 | 4.7 | 28.8 |
| **ID** | **Site** | **Taxon** | **Element** | ***δ*¹³C _VPDB_** | ***δ*¹⁸O _VPDB_** | ***δ*¹⁸O _SMOW_** | **CO_3_ (%)** | **Distance from ERJ (mm)** |
| NME-51f | Naame | *Bos primigenius* | M3 | -9.9 | -5.7 | 25.0 | 4.7 | 25.7 |
| NME-51g | Naame | *Bos primigenius* | M3 | -10.2 | -6.6 | 24.1 | 4.8 | 22.8 |
| NME-51h | Naame | *Bos primigenius* | M3 | -11.1 | -7.7 | 22.9 | 4.9 | 19.8 |
| NME-51i | Naame | *Bos primigenius* | M3 | -11.8 | -7.4 | 23.3 | 5.0 | 16.8 |
| NME-51j | Naame | *Bos primigenius* | M3 | -11.7 | -8.1 | 22.6 | 5.0 | 13.8 |
| NME-51k | Naame | *Bos primigenius* | M3 | -11.6 | -7.3 | 23.4 | 5.0 | 10.7 |
| NME-51l | Naame | *Bos primigenius* | M3 | -11.4 | -7.4 | 23.3 | 5.4 | 7.5 |
| NME-51m | Naame | *Bos primigenius* | M3 | -11.4 | -6.7 | 24.0 | 5.7 | 4.3 |
| NME8a | Naame | *Dama mesopotamica* | m1 or m2 | -11.8 | -5.4 | 25.4 | 4.7 | 14.7 |
| NME8b | Naame | *Dama mesopotamica* | m1 or m2 | -12.0 | -5.0 | 25.8 | 4.8 | 11.6 |
| NME8c | Naame | *Dama mesopotamica* | m1 or m2 | -12.1 | -5.6 | 25.1 | 4.6 | 9.9 |
| NME8d | Naame | *Dama mesopotamica* | m1 or m2 | -12.5 | -4.9 | 25.8 | 4.6 | 7.5 |
| NME8e | Naame | *Dama mesopotamica* | m1 or m2 | -12.7 | -4.0 | 26.8 | 4.6 | 4.8 |
| REK-16a | Ras el Kelb | *Cervus elaphus* | M3 | -11.4 | -4.8 | 26.0 | 3.9 | 15.0 |
| REK-16b | Ras el Kelb | *Cervus elaphus* | M3 | -11.5 | -4.5 | 26.3 | 4.1 | 12.2 |
| REK-16c | Ras el Kelb | *Cervus elaphus* | M3 | -11.6 | -4.4 | 26.4 | 4.1 | 9.2 |
| REK-16d | Ras el Kelb | *Cervus elaphus* | M3 | -11.8 | -5.0 | 25.8 | 4.2 | 6.0 |
| REK-16e | Ras el Kelb | *Cervus elaphus* | M3 | -12.1 | -5.9 | 24.9 | 4.3 | 2.4 |
| REK-25a | Ras el Kelb | *Bos primigenius* | m2 | -11.3 | -6.7 | 24.0 | 4.6 | 38.3 |
| REK-25b | Ras el Kelb | *Bos primigenius* | m2 | -11.3 | -6.8 | 23.9 | 4.4 | 34.1 |
| REK-25c | Ras el Kelb | *Bos primigenius* | m2 | -11.2 | -7.0 | 23.7 | 4.6 | 30.9 |
| ***REK-25d*** | ***Ras el Kelb*** | ***Bos primigenius*** | ***m2*** | ***-11.3*** | ***-8.2*** | ***22.4*** | ***4.2*** | ***28.6*** |
| REK-25e | Ras el Kelb | *Bos primigenius* | m2 | -11.4 | -7.5 | 23.2 | 4.3 | 26.0 |
| REK-25f | Ras el Kelb | *Bos primigenius* | m2 | -11.6 | -7.6 | 23.1 | 4.4 | 23.3 |
| REK-25g | Ras el Kelb | *Bos primigenius* | m2 | -11.6 | -7.7 | 22.9 | 4.4 | 20.6 |
| REK-25h | Ras el Kelb | *Bos primigenius* | m2 | -11.5 | -8.0 | 22.7 | 4.3 | 18.1 |
| REK-25i | Ras el Kelb | *Bos primigenius* | m2 | -11.4 | -7.6 | 23.0 | 4.3 | 14.4 |
| REK-25j | Ras el Kelb | *Bos primigenius* | m2 | -11.4 | -7.7 | 23.0 | 4.7 | 11.3 |
| REK-25k | Ras el Kelb | *Bos primigenius* | m2 | -11.4 | -7.2 | 23.5 | 4.4 | 8.5 |
| REK-25l | Ras el Kelb | *Bos primigenius* | m2 | -11.4 | -7.0 | 23.7 | 5.0 | 5.5 |
| REK-29a | Ras el Kelb | *Bos primigenius* | m3 | -12.1 | -4.8 | 26.0 | 5.0 | 39.8 |
| REK-29b | Ras el Kelb | *Bos primigenius* | m3 | -11.9 | -4.9 | 25.9 | 5.3 | 37.0 |
| REK-29c | Ras el Kelb | *Bos primigenius* | m3 | -11.7 | -4.9 | 25.9 | 5.0 | 34.3 |
| REK-29d | Ras el Kelb | *Bos primigenius* | m3 | -11.4 | -5.0 | 25.8 | 4.9 | 32.0 |
| REK-29e | Ras el Kelb | *Bos primigenius* | m3 | -11.2 | -4.7 | 26.0 | 4.9 | 29.3 |
| REK-29f | Ras el Kelb | *Bos primigenius* | m3 | -11.1 | -4.5 | 26.3 | 4.9 | 26.8 |
| REK-29g | Ras el Kelb | *Bos primigenius* | m3 | -11.2 | -4.6 | 26.2 | 4.9 | 23.8 |
| REK-29h | Ras el Kelb | *Bos primigenius* | m3 | -11.3 | -5.3 | 25.5 | 5.1 | 21.1 |
| REK-29i | Ras el Kelb | *Bos primigenius* | m3 | -11.2 | -6.1 | 24.6 | 4.7 | 18.1 |
| REK-29j | Ras el Kelb | *Bos primigenius* | m3 | -11.4 | -6.2 | 24.6 | 4.7 | 15.4 |
| REK-29k | Ras el Kelb | *Bos primigenius* | m3 | -11.4 | -5.9 | 24.8 | 4.9 | 12.9 |
| REK-29l | Ras el Kelb | *Bos primigenius* | m3 | -11.6 | -5.8 | 24.9 | 4.8 | 10.3 |
| **ID** | **Site** | **Taxon** | **Element** | ***δ*¹³C _VPDB_** | ***δ*¹⁸O _VPDB_** | ***δ*¹⁸O _SMOW_** | **CO_3_ (%)** | **Distance from ERJ (mm)** |
| REK-29m | Ras el Kelb | *Bos primigenius* | m3 | -11.6 | -4.7 | 26.1 | 5.0 | 8.1 |
| REK-29n | Ras el Kelb | *Bos primigenius* | m3 | -11.8 | -4.8 | 25.9 | 5.1 | 5.3 |
| REK-41a | Ras el Kelb | *Cervus elaphus* | M3 | -12.1 | -5.4 | 25.3 | 4.7 | 15.8 |
| REK-41b | Ras el Kelb | *Cervus elaphus* | M3 | -12.0 | -5.3 | 25.5 | 4.9 | 12.8 |
| REK-41c | Ras el Kelb | *Cervus elaphus* | M3 | -11.9 | -5.5 | 25.3 | 4.8 | 10.4 |
| REK-41d | Ras el Kelb | *Cervus elaphus* | M3 | -11.8 | -4.6 | 26.2 | 4.8 | 8.0 |
| REK-41e | Ras el Kelb | *Cervus elaphus* | M3 | -11.7 | -3.7 | 27.1 | 4.7 | 5.4 |
| REK-41f | Ras el Kelb | *Cervus elaphus* | M3 | -11.4 | -2.9 | 28.0 | 4.8 | 3.1 |
| REK-45a | Ras el Kelb | *Bos primigenius* | M3 | -11.5 | -5.3 | 25.5 | 4.6 | 49.1 |
| ***REK-45b*** | ***Ras el Kelb*** | ***Bos primigenius*** | ***M3*** | ***-11.3*** | ***-4.6*** | ***26.1*** | ***4.6*** | ***46.2*** |
| REK-45c | Ras el Kelb | *Bos primigenius* | M3 | -11.2 | -5.1 | 25.6 | 4.6 | 43.5 |
| REK-45d | Ras el Kelb | *Bos primigenius* | M3 | -11.2 | -5.0 | 25.8 | 4.4 | 41.1 |
| REK-45e | Ras el Kelb | *Bos primigenius* | M3 | -11.0 | -4.4 | 26.4 | 4.7 | 38.6 |
| REK-45f | Ras el Kelb | *Bos primigenius* | M3 | -11.1 | -4.3 | 26.5 | 4.4 | 36.2 |
| REK-45g | Ras el Kelb | *Bos primigenius* | M3 | -10.8 | -4.3 | 26.5 | 4.4 | 33.1 |
| REK-45h | Ras el Kelb | *Bos primigenius* | M3 | -10.8 | -4.5 | 26.3 | 4.3 | 30.7 |
| REK-45i | Ras el Kelb | *Bos primigenius* | M3 | -11.0 | -4.9 | 25.9 | 4.4 | 27.5 |
| ***REK-45j*** | ***Ras el Kelb*** | ***Bos primigenius*** | ***M3*** | ***-11.0*** | ***-4.5*** | ***26.3*** | ***4.7*** | ***25.2*** |
| REK-45k | Ras el Kelb | *Bos primigenius* | M3 | -10.9 | -5.1 | 25.7 | 4.7 | 22.4 |
| ***REK-45l*** | ***Ras el Kelb*** | ***Bos primigenius*** | ***M3*** | ***-11.0*** | ***-4.9*** | ***25.8*** | ***4.6*** | ***19.5*** |
| REK-45m | Ras el Kelb | *Bos primigenius* | M3 | -10.8 | -5.7 | 25.0 | 4.4 | 16.8 |
| REK-45n | Ras el Kelb | *Bos primigenius* | M3 | -10.8 | -5.1 | 25.6 | 4.6 | 13.9 |
| ***REK-45o*** | ***Ras el Kelb*** | ***Bos primigenius*** | ***M3*** | ***-10.9*** | ***-5.6*** | ***25.1*** | ***4.5*** | ***10.9*** |
| REK-45p | Ras el Kelb | *Bos primigenius* | M3 | -10.9 | -4.7 | 26.1 | 4.6 | 8.1 |
| REK-45q | Ras el Kelb | *Bos primigenius* | M3 | -11.0 | -5.0 | 25.7 | 4.9 | 4.8 |
| REK-48a | Ras el Kelb | *Stephanorhinus hemitoechus* | Indet. Molar | -13.1 | -7.2 | 23.5 | 4.1 | 33.9 |
| REK-48b | Ras el Kelb | *Stephanorhinus hemitoechus* | Indet. Molar | -13.1 | -7.5 | 23.2 | 3.5 | 30.5 |
| REK-48c | Ras el Kelb | *Stephanorhinus hemitoechus* | Indet. Molar | -12.8 | -7.2 | 23.5 | 3.9 | 28.2 |
| REK-48d | Ras el Kelb | *Stephanorhinus hemitoechus* | Indet. Molar | -12.8 | -7.2 | 23.5 | 3.8 | 25.1 |
| REK-48e | Ras el Kelb | *Stephanorhinus hemitoechus* | Indet. Molar | -12.9 | -6.6 | 24.1 | 3.8 | 22.1 |
| REK-48f | Ras el Kelb | *Stephanorhinus hemitoechus* | Indet. Molar | -13.0 | -6.6 | 24.1 | 3.8 | 19.4 |
| REK-48g | Ras el Kelb | *Stephanorhinus hemitoechus* | Indet. Molar | -12.9 | -6.8 | 23.9 | 4.1 | 16.3 |
| REK-48h | Ras el Kelb | *Stephanorhinus hemitoechus* | Indet. Molar | -13.0 | -6.9 | 23.8 | 4.0 | 13.8 |
| REK-48i | Ras el Kelb | *Stephanorhinus hemitoechus* | Indet. Molar | -13.0 | -6.8 | 23.9 | 3.9 | 10.8 |
| REK-48j | Ras el Kelb | *Stephanorhinus hemitoechus* | Indet. Molar | -12.9 | -6.9 | 23.8 | 4.0 | 8.0 |
| REK-48k | Ras el Kelb | *Stephanorhinus hemitoechus* | Indet. Molar | -13.0 | -6.5 | 24.2 | 4.4 | 5.6 |
| REK-48l | Ras el Kelb | *Stephanorhinus hemitoechus* | Indet. Molar | -13.0 | -6.2 | 24.5 | 4.5 | 2.8 |
| REK-49a | Ras el Kelb | *Stephanorhinus hemitoechus* | Indet. Molar | -13.0 | -5.8 | 24.9 | 3.6 | 36.5 |
|  |  |  |  |  |  |  |  |  |
| **ID** | **Site** | **Taxon** | **Element** | ***δ*¹³C _VPDB_** | ***δ*¹⁸O _VPDB_** | ***δ*¹⁸O _SMOW_** | **CO_3_ (%)** | **Distance from ERJ (mm)** |
| REK-49b | Ras el Kelb | *Stephanorhinus hemitoechus* | Indet. Molar | -12.9 | -5.6 | 25.1 | 3.5 | 33.9 |
| REK-49c | Ras el Kelb | *Stephanorhinus hemitoechus* | Indet. Molar | -12.9 | -5.3 | 25.5 | 3.7 | 31.3 |
| ***REK-49d*** | ***Ras el Kelb*** | ***Stephanorhinus hemitoechus*** | ***Indet. Molar*** | ***-12.7*** | ***-5.9*** | ***24.9*** | ***3.6*** | ***28.4*** |
| ***REK-49e*** | ***Ras el Kelb*** | ***Stephanorhinus hemitoechus*** | ***Indet. Molar*** | ***-12.6*** | ***-6.1*** | ***24.7*** | ***3.5*** | ***25.7*** |
| REK-49f | Ras el Kelb | *Stephanorhinus hemitoechus* | Indet. Molar | -12.6 | -5.5 | 25.2 | 3.5 | 23.2 |
| REK-49g | Ras el Kelb | *Stephanorhinus hemitoechus* | Indet. Molar | -12.5 | -5.6 | 25.1 | 3.6 | 20.9 |
| REK-49h | Ras el Kelb | *Stephanorhinus hemitoechus* | Indet. Molar | -12.6 | -5.8 | 25.0 | 3.5 | 18.6 |
| REK-49i | Ras el Kelb | *Stephanorhinus hemitoechus* | Indet. Molar | -12.6 | -5.7 | 25.0 | 3.6 | 16.3 |
| ***REK-49j*** | ***Ras el Kelb*** | ***Stephanorhinus hemitoechus*** | ***Indet. Molar*** | ***-12.6*** | ***-6.3*** | ***24.4*** | ***3.5*** | ***14.5*** |
| REK-49k | Ras el Kelb | *Stephanorhinus hemitoechus* | Indet. Molar | -12.7 | -5.8 | 24.9 | 3.7 | 11.5 |
| ***REK-49l*** | ***Ras el Kelb*** | ***Stephanorhinus hemitoechus*** | ***Indet. Molar*** | ***-12.7*** | ***-5.7*** | ***25.1*** | ***3.8*** | ***9.1*** |
| REK-49m | Ras el Kelb | *Stephanorhinus hemitoechus* | Indet. Molar | -12.5 | -6.2 | 24.5 | 3.8 | 6.8 |
| REK-49n | Ras el Kelb | *Stephanorhinus hemitoechus* | Indet. Molar | -12.2 | -6.0 | 24.7 | 4.1 | 4.4 |
| REK-49o | Ras el Kelb | *Stephanorhinus hemitoechus* | Indet. Molar | -12.2 | -5.8 | 24.9 | 4.1 | 1.9 |

**Table S5. Inverse-modelled** ***δ*^18^O values and confidence intervals for sampled teeth.** Inverse-modelled enamel *δ*^18^O_VSMOW_ values derived from sequential *δ*^18^O_VPDB_ data. The table reports mean, lower, and upper 95% confidence interval (CI) estimates for each specimen.

| **Tooth ID** | **CI length** | **Lower CI** | **Upper CI** | **Mean** | **Extremum** |
| --- | --- | --- | --- | --- | --- |
| NIM 16 Wild Goat | 307 | 12.33 | 28.46 | 20.48 | Winter |
| NIM 16 Wild Goat | 253 | 29.19 | 44.04 | 35.16 | Summer |
| NIM 20 Wild Goat | 96 | 14.90 | 30.49 | 22.63 | Winter |
| NIM 20 Wild Goat | 210 | 24.92 | 38.63 | 30.61 | Summer |
| NIM 20 Wild Goat | 305 | 11.30 | 27.58 | 20.15 | Winter |
| NIM 27 Aurochs | 91 | 21.59 | 25.39 | 23.32 | Winter |
| NIM 27 Aurochs | 205 | 25.39 | 28.59 | 27.08 | Summer |
| NIM 28 Aurochs | 252 | 23.28 | 27.28 | 25.29 | Winter |
| NIM 28 Aurochs | 151 | 29.75 | 33.02 | 31.36 | Summer |
| NIM 3 Aurochs | 50.2 | 28.20 | 31.68 | 29.85 | Summer |
| NIM 3 Aurochs | 128 | 18.87 | 23.16 | 21.06 | Winter |
| NIM 3 Aurochs | 364.2 | 30.91 | 34.27 | 32.44 | Summer |
| NIM 3 Aurochs | 418.8 | 23.02 | 26.24 | 24.44 | Winter |
| NME 11 Wild Goat | 162 | 11.49 | 27.45 | 22.30 | Winter |
| NME 11 Wild Goat | 120 | 28.18 | 35.29 | 30.93 | Summer |
| NME 15 Wild Goat | 162.4 | 7.55 | 29.29 | 20.68 | Winter |
| NME 15 Wild Goat | 106.7 | 22.49 | 45.60 | 34.89 | Summer |
| NME 35 Aurochs | 229.8 | 19.95 | 23.11 | 21.42 | Winter |
| NME 35 Aurochs | 83.3 | 28.78 | 32.28 | 30.69 | Summer |
| REK 29 Aurochs | 211.6 | 27.30 | 30.67 | 28.95 | Summer |
| REK 29 Aurochs | 339.8 | 21.39 | 24.77 | 22.88 | Winter |
| REK 29 Aurochs | 370.2 | 26.44 | 29.59 | 27.93 | Summer |
| NME 43 Equus | 304 | 3.35 | 22.18 | 13.50 | Winter |
| NME 43 Equus | 326 | 25.69 | 47.40 | 34.86 | Summer |

**Table S6.** **Derived oxygen *δ*^18^O values from aurochs (*Bos primigenius*) for paleoenviromental reconstructions.** The table reports measured enamel phosphate values (*δ*^18^O_phos_, VSMOW) and derived estimates of drinking water (*δ*^18^O_dw_, VSMOW) and meteoric water (*δ*^18^O_mw_, VSMOW), with associated calculation errors (1 *σ*). from Nahr Ibrahim, Naame, and Ras el-Kelb. Estimated *δ*^18^O_dw_ values were calculated from enamel phosphate using species-specific conversion equations, with associated error margins. (*) Represent directly dated specimens.

| **Site** | **Sample ID** | **Species** | **Element** | ***δ*^18^O_phos_**  **(‰, VSMOW)** | **Estimated *δ*^18^O_dw_ (‰, VSMOW)** | **Estimated *δ*^18^O_dw_ error** | ***δ*^18^O_mw_**  **(‰, VSMOW)** |
| --- | --- | --- | --- | --- | --- | --- | --- |
| Nahr Ibrahim | NIM2* | *Bos primigenius* | M2 | 16.13 | -7 | 2.7 | -8.0 |
| Nahr Ibrahim | NIM25 | *Bos primigenius* | m2 | 17.57 | -4.9 | 2.7 | -6.1 |
| Nahr Ibrahim | NIM26 | *Bos primigenius* | m2 | 17.61 | -4.8 | 2.7 | -6.1 |
| Nahr Ibrahim | NIM27 | *Bos primigenius* | m3 | 17.01 | -5.7 | 2.7 | -6.9 |
| Nahr Ibrahim | NIM28 | *Bos primigenius* | m3 | 18.75 | -3.2 | 2.8 | -4.6 |
| Nahr Ibrahim | NIM3 | *Bos primigenius* | M3 | 18.08 | -4.2 | 2.7 | -5.5 |
| Naame - PIII_0_ | NME35 | *Bos primigenius* | M3 | 16.85 | -6 | 2.7 | -7.1 |
| Naame - PIII_0_ | NME36 | *Bos primigenius* | M1 or 2 | 19.07 | -2.7 | 2.8 | -4.2 |
| Naame - PIII_2_ | NME49 | *Bos primigenius* | P2 | 19.58 | -2 | 2.8 | -3.6 |
| Naame - NS | NME51* | *Bos primigenius* | M2-3 | 16.24 | -6.8 | 2.7 | -9.4 |
| Ras el-Kelb | REK25 | *Bos primigenius* | m2 | 14.80 | -8.9 | 2.6 | -9.7 |
| Ras el-Kelb | REK29 | *Bos primigenius* | m3 | 16.92 | -5.9 | 2.7 | -7.0 |
| Ras el-Kelb | REK35 | *Bos primigenius* | P | 17.01 | -5.7 | 2.7 | -6.9 |
| Ras el-Kelb | REK38 | *Bos primigenius* | p4 | 17.60 | -4.9 | 2.7 | -6.1 |
| Ras el-Kelb | REK45 | *Bos primigenius* | M3 | 17.21 | -5.4 | 2.7 | -6.6 |

**Table S7. Reconstructed mean annual and seasonal temperature and precipitation estimates for the studied sites.** Mean air temperature (MAT, °C) and mean annual precipitation (MAP, mm) values are shown, with reported errors representing propagated analytical and model uncertainties. Offsets from modern climatic conditions indicate deviations relative to present-day values. Modern estimates were obtained from [www.tutiempo.it](http://www.tutiempo.it) (Beirut Airport station), and mean annual precipitation values represent averages from 2000 to 2020.

| **Site** | **Season** | **N MAT** | **Mean T (C°)** | **Error T (C°)** | **Offset Modern T (C°)** | **N MAP** | **MAP (mm)** | **MAP mm (95% CI)** | **Offset modern MAP (mm)** |
| --- | --- | --- | --- | --- | --- | --- | --- | --- | --- |
| Naame - *Niveau Supérieur* | Mean annual | 1 | 15.9 | 1.3 | -5.6 | 1 | 595 |  | -195 |
| Naame - PIII_2_ | Mean annual |  |  |  |  | 4 | 991 | 347 | 265 |
| Naame - PIII_0_ | Mean annual |  |  |  |  | 19 | 1055 | 214 | 202 |
| Naame | Summer | 1 | 38.5 | 10.5 | 12.4 |  |  |  |  |
|  | Mean annual | 3 | 25.1 | 3.9 | 3.6 |  |  |  |  |
|  | Winter | 1 | 2.1 | 8.1 | -9.5 |  |  |  |  |
| Nahr Ibrahim | Summer | 3 | 35.2 | 8.9 | 9.1 |  |  |  |  |
|  | Mean annual | 6 | 21.1 | 2.3 | -0.4 | 14 | 811 | 114.8 | 21.7 |
|  | Winter | 3 | 11.5 | 3.5 | -0.1 |  |  |  |  |
| Ras el-Kelb | Summer | 1 | 29.6 | 6 | 3.5 |  |  |  |  |
|  | Mean annual | 5 | 17.7 | 1.8 | -3.8 | 13 | 807.9 | 127.6 | 18.6 |
|  | Winter | 1 | 7.9 | 5.2 | -3.7 |  |  |  |  |

## **S6. Supplementary References**

1.	Fleisch, H. Habitats du Paléolithique Moyen a Naamé. *Bulletin du Musée de Beyrouth* XIII, 25–39 (1970).

2.	Sanlaville, P. Etude Géomorphologique de la région littorale du Liban. (Université Libanaise, 1977).

3.	Fleisch, H. La destruction des habitats du Paléolithique moyen à Naamé (Liban). *Ann Georgr* 27–38 (1980).

4.	Russo, G. *et al.* Lebanese legacy collections reveal nearly 400,000 years of human adaptation to Mediterranean woodlands. *under review*.

5.	Zumoffen, G. *La Phénicie Avant Les Phéniciens: L’age de La Pierre*. (Beyrouth: Imprimerie Catholique, Beirut, 1900).

6.	Solecki, Ralph. S. The Middle Paleolithic site of Nahr Ibrahim (Asfourieh Cave) in Lebanon. in *Problems in Prehistory: North Africa and Levant* (eds. Wendorf, F. & Marks, A. E.) 283–295 (Southern Methodist University Press, Dallas, 1975).

7.	Zumoffen, G. L’âge de la pierre en Phénicie . *Anthropos* 3, 431–455 (1908).

8.	Solecki, R. S. Summary report of the Columbia University prehistoric investigations in Lebanon, season 1969. *Bulletin du Musée de Beyrouth* 23, 95–128 (1970).

9.	Porat, N. & Schwarcz, H. P. Use of signal subtraction methods in ESR dating of burned flint. *Int J Rad Appl Instrum D* 18, 203–212 (1991).

10.	Shaw, A. Lower and Middle Palaeolithic of Lebanon. in *Quaternary of the Levant* 555–566 (Cambridge University Press, 2017).

11.	Schwarcz, H. P. Uranium-Series Dating and the Origin of Modern Man. *Philosophical Transactions: Biological Sciences* 337, 131–137 (1992).

12.	Tristram, H. B. *The Land of Israel; a Journal of Travels in Palestine*. (Society for Promoting Christian Knowledge, 1866).

13.	Fleisch, H. Le Rhinoceros de Merck dans la grotte levalloiso-mousterienne de Ras-el-Kelbe. *Anthropologie* 59, 168–170 (1955).

14.	Garrod, D. & Henri-Martin, G. Rapport préliminaire sur la fouille d’une grotte à Ras el Kelb, Liban, 1959. *Bulletin du Musée de Beyrouth* XVI, 61–67 (1961).

15.	Copeland, L., The Middle Palaeolithic of Adlun and Ras el Kelb (Lebanon) : First results from a study of the flint industries. *Paléorient* 4, 33–57 (1978).

16.	Copeland, L. & Moloney, N. *The Mousterian Site of Ras El-Kelb, Lebanon*. vol. 706 (BAR International Series, 1998).

17.	Kohn, M. J. Carbon isotope compositions of terrestrial C3 plants as indicators of (paleo)ecology and (paleo)climate. *Proc Natl Acad Sci U S A* 107, 19691–19695 (2010).

18.	Heaton, T. H. E. Spatial, Species, and Temporal Variations in the^13^C/^12^C Ratios of C3Plants: Implications for Palaeodiet Studies. *J Archaeol Sci* 26, 637–649 (1999).

19.	Diefendorf, A. F., Mueller, K. E., Wing, S. L., Koch, P. L. & Freeman, K. H. Global patterns in leaf ^13^C discrimination and implications for studies of past and future climate. *Proc Natl Acad Sci U S A* 107, 5738–5743 (2010).

20.	Kohn, M. J. Carbon isotope discrimination in C3 land plants is independent of natural variations in pCO_2_. *Geochem Perspect Lett* 2, 35–43 (2016).

21.	Drucker, D. G., Bridault, A., Hobson, K. A., Szuma, E. & Bocherens, H. Can carbon-13 in large herbivores reflect the canopy effect in temperate and boreal ecosystems? Evidence from modern and ancient ungulates. *Palaeogeogr Palaeoclimatol Palaeoecol* 266, 69–82 (2008).

22.	Ambrose, S. H. & Norr, L. Experimental Evidence for the Relationship of the Carbon Isotope Ratios of Whole Diet and Dietary Protein to Those of Bone Collagen and Carbonate. *Prehistoric Human Bone* 1–37 (1993).

23.	Balasse, M., Bocherens, H. & Mariotti, A. Intra-bone Variability of Collagen and Apatite Isotopic Composition Used as Evidence of a Change of Diet. *J Archaeol Sci* 26, 593–598 (1999).

24.	Szostek, K. Chemical signals and reconstruction of life strategies from ancient human bones and teeth – problems and perspectives. *Anthropological Review* 72, 3–30 (2009).

25.	Lécuyer, C., Hillaire-Marcel, C., Burke, A., Julien, M. A. & Hélie, J. F. Temperature and precipitation regime in LGM human refugia of southwestern Europe inferred from *δ*^13^C and *δ*^18^O of large mammal remains. *Quat Sci Rev* 255, 106796 (2021).

26.	Kohn, M. J. & Cerling, T. E. Stable Isotope Compositions of Biological Apatite. *Rev Mineral Geochem* 48, 455–488 (2002).

27.	Longinelli, A. Oxygen isotopes in mammal bone phosphate: A new tool for paleohydrological and paleoclimatological research? *Geochim Cosmochim Acta* 48, 385–390 (1984).

28.	Kohn, M. J., Schoeninger, M. J. & Valley, J. W. Herbivore tooth oxygen isotope compositions: Effects of diet and physiology. *Geochim Cosmochim Acta* 60, 3889–3896 (1996).

29.	Hoppe, K. A., Stover, S. M., Pascoe, J. R. & Amundson, R. Tooth enamel biomineralization in extant horses: implications for isotopic microsampling. *Palaeogeogr Palaeoclimatol Palaeoecol* 206, 355–365 (2004).

30.	Fricke, H. C., Clyde, W. C. & O’Neil, J. R. Intra-tooth variations in δ18O (PO4) of mammalian tooth enamel as a record of seasonal variations in continental climate variables. *Geochim Cosmochim Acta* 62, 1839–1850 (1998).

31.	Levin, N. E., Cerling, T. E., Passey, B. H., Harris, J. M. & Ehleringer, J. R. A stable isotope aridity index for terrestrial environments. *Proc Natl Acad Sci U S A* 103, 11201–11205 (2006).

32.	Rozanski, K., Araguás-Araguás, L. & Gonfiantini, R. Relation Between Long-Term Trends of Oxygen-18 Isotope Composition of Precipitation and Climate. *Science (1979)* 258, 981–985 (1992).

33.	Dansgaard, W. Stable isotopes in precipitation. *Tellus* 16, 436–468 (1964).

34.	Pederzani, S. & Britton, K. Oxygen isotopes in bioarchaeology: Principles and applications, challenges and opportunities. *Earth Sci Rev* 188, 77–107 (2019).

35.	Pederzani, S. *et al.* Subarctic climate for the earliest *Homo sapiens* in Europe. *Sci Adv* 7, 4642–4664 (2021).

36.	Fernández-García, M. *et al.* Palaeoecology of ungulates in northern Iberia during the Late Pleistocene through isotopic analysis of teeth. *Biogeosciences* 21, 4413–4437 (2024).

37.	Pederzani, S. *et al.* Stable isotopes show Homo sapiens dispersed into cold steppes ~45,000 years ago at Ilsenhöhle in Ranis, Germany. *Nature Ecology & Evolution 2024* 1–11 (2024).

38.	Brown, W. A. B. & Chapman, N. G. The dentition of fallow deer (*Dama dama*): a scoring scheme to assess age from wear of the permanent molariform teeth. *J Zool* 221, 659–682 (1990).

39.	Brown, W. A. B. & Chapman, N. G. The dentition of red deer (*Cervus elaphus*): a scoring scheme to assess age from wear of the permanent molariform teeth. *J Zool* 224, 519–536 (1991).

40.	Lister, A. M. & Chapman, N. G. Growth and Variation in Fallow Deer (*Dama dama* L.) From Two Contrasting Habitats in Southern Britain. *Acta Zoologica* 0, 1–24 (2025).

41.	Beasley, M. J., Brown, W. A. B. & Legge, A. J. Incremental banding in dental cementum: Methods of preparation for teeth from archaeological sites and for modern comparative specimens. *Int J Osteoarchaeol* 2, 37–50 (1992).

42.	Brown, W. A. B., Christofferson, D. V. M., Massler, M. & Weiss, M. B. Postnatal tooth development in cattle. *Am J Vet Res* 21, 7–34 (1960).

43.	Hoppe, K. A. Correlation between the oxygen isotope ratio of North American bison teeth and local waters: Implication for paleoclimatic reconstructions. *Earth Planet Sci Lett* 244, 408–417 (2006).

44.	Zazzo, A. *et al.* The isotope record of short- and long-term dietary changes in sheep tooth enamel: Implications for quantitative reconstruction of paleodiets. *Geochim Cosmochim Acta* 74, 3571–3586 (2010).

45.	Milhaud, G. & Nezit, J. Molar development in sheep: morphology, radiography, microhardness; Développement des molaires chez le mouton. Études morphologique, radiographique et microdurométrique. *Rec Med Vet Ec Alfort* 167, (1991).

46.	Moran, N. C. & O’Connor, T. P. Age attribution in domestic sheep by skeletal and dental maturation: A pilot study of available sources. *Int J Osteoarchaeol* 4, 267–285 (1994).

47.	Martin, C., Bentaleb, I., Kaandorp, R., Iacumin, P. & Chatri, K. Intra-tooth study of modern rhinoceros enamel *δ*^18^O: Is the difference between phosphate and carbonate *δ*^18^O a sound diagenetic test? https://doi.org/10.1016/j.palaeo.2008.03.039 (2008).

48.	Tafforeau, P., Bentaleb, I., Jaeger, J. J. & Martin, C. Nature of laminations and mineralization in rhinoceros enamel using histology and X-ray synchrotron microtomography: Potential implications for palaeoenvironmental isotopic studies. *Palaeogeogr Palaeoclimatol Palaeoecol* 246, 206–227 (2007).

49.	Anderson, J. L. Tooth replacement and dentition of the black rhinoceros (*Diceros bicornis* Linn.). *Lammergeyer* 6, 41–46 (1966).

50.	Cerling, T. E., Harris, J. M. & Leakey, M. G. Browsing and grazing in elephants: The isotope record of modern and fossil proboscideans. *Oecologia* 120, 364–374 (1999).

51.	Tejada-Lara, J. V. *et al.* Body mass predicts isotope enrichment in herbivorous mammals. *Proceedings of the Royal Society B* 285, (2018).

52.	Cerling, T. E. *et al.* CH_4_/CO_2_ Ratios and Carbon Isotope Enrichment Between Diet and Breath in Herbivorous Mammals. *Front Ecol Evol* 9, 638568 (2021).

53.	Garrard, A. N. Man-Animal-Plant Relationships during the Upper Pleistocene and Early Holocene. (University of Cambridge, Cambridge, 1980).

54.	Bocherens, H. Isotopic biogeochemistry and the paleoecology of the mammoth steppe fauna. *Deinsea* 9, 57–76 (2003).

55.	Bereiter, B. *et al.* Revision of the EPICA Dome C CO_2_ record from 800 to 600 kyr before present. *Geophys Res Lett* 42, 542–549 (2015).

56.	Eggleston, S., Schmitt, J., Bereiter, B., Schneider, R. & Fischer, H. Evolution of the stable carbon isotope composition of atmospheric CO2 over the last glacial cycle. *Paleoceanography* 31, 434–452 (2016).

57.	Rey, K. *et al.* Late Miocene climatic and environmental variations in northern Greece inferred from stable isotope compositions (*δ*^18^O, *δ*^13^C) of equid teeth apatite. *Palaeogeogr Palaeoclimatol Palaeoecol* 388, 48–57 (2013).

58.	Passey, B. H. *et al.* Inverse methods for estimating primary input signals from time-averaged isotope profiles. *Geochim Cosmochim Acta* 69, 4101–4116 (2005).

59.	Kohn, M. J. Comment: Tooth Enamel Mineralization in Ungulates: Implications for Recovering a Primary Isotopic Time-Series, by B. H. Passey and T. E. Cerling (2002). *Geochim Cosmochim Acta* 68, 403–405 (2004).

60.	Kierdorf, H., Witzel, C., Upex, B., Dobney, K. & Kierdorf, U. Enamel hypoplasia in molars of sheep and goats, and its relationship to the pattern of tooth crown growth. *J Anat* 220, 484–495 (2012).

61.	Fraser, D., Kim, S. L., Welker, J. M. & Clementz, M. T. Pronghorn (*Antilocapra americana*) enamel phosphate δ18O values reflect climate seasonality: Implications for paleoclimate reconstruction. *Ecol Evol* 11, 17005–17021 (2021).

62.	Stevens, R. E., Pederzani, S., Britton, K. & Wexler, S. K. Bones and teeth isotopes as archives for palaeoclimatic, palaeoenvironmental and palaeoecological data. *Quat Sci Rev* 357, (2025).

63.	Koeniger, P., Toll, M. & Himmelsbach, T. Stable isotopes of precipitation and spring waters reveal an altitude effect in the Anti-Lebanon Mountains, Syria. *Hydrol Process* 30, 2851–2860 (2016).

64.	Bryant, J. D., Koch, P. L., Froelich, P. N., Showers, W. J. & Genna, B. J. Oxygen isotope partitioning between phosphate and carbonate in mammalian apatite. *Geochim Cosmochim Acta* 60, 5145–5148 (1996).

65.	Iacumin, P., Bocherens, H., Mariotti, A. & Longinelli, A. Oxygen isotope analyses of co-existing carbonate and phosphate in biogenic apatite: a way to monitor diagenetic alteration of bone phosphate? *Earth Planet Sci Lett* 142, 1–6 (1996).

66.	Pellegrini, M., Lee-Thorp, J. A. & Donahue, R. E. Exploring the variation of the *δ*^18^O_p_ and *δ*^18^O_c_ relationship in enamel increments. *Palaeogeogr Palaeoclimatol Palaeoecol* 310, 71–83 (2011).

67.	Pederzani, S. *et al.* Late Pleistocene Neanderthal exploitation of stable and mosaic ecosystems in northern Iberia shown by multi-isotope evidence. *Quat Res* 1–25 (2023).

68.	Pryor, A. J. E., Stevens, R. E., O’Connell, T. C. & Lister, J. R. Quantification and propagation of errors when converting vertebrate biomineral oxygen isotope data to temperature for palaeoclimate reconstruction. *Palaeogeogr Palaeoclimatol Palaeoecol* 412, 99–107 (2014).

69.	Skrzypek, G., Winiewski, A. & Grierson, P. F. How cold was it for Neanderthals moving to Central Europe during warm phases of the last glaciation? *Quat Sci Rev* 30, 481–487 (2011).

70.	Blumenthal, S. A. *et al.* Aridity and hominin environments. *Proc Natl Acad Sci U S A* 114, 7331–7336 (2017).

71.	Faith, J. T. Paleodietary change and its implications for aridity indices derived from δ18O of herbivore tooth enamel. *Palaeogeogr Palaeoclimatol Palaeoecol* 490, 571–578 (2018).

72.	Silanikove, N. The physiological basis of adaptation in goats to harsh environments. *Small Ruminant Research* 35, 181–193 (2000).

73.	Luyt, J., Faith, J. T. & Sealy, J. Large herbivore *δ*^18^O as a proxy for aridity in the South African winter and year-round rainfall zone. *Quat Res* 1–14 (2024) doi:10.1017/QUA.2024.21.

74.	Hallin, K. A., Schoeninger, M. J. & Schwarcz, H. P. Paleoclimate during Neandertal and anatomically modern human occupation at Amud and Qafzeh, Israel: the stable isotope data. *J Hum Evol* 62, 59–73 (2012).
